# Effect modifiers of virtual reality in pain management: a systematic review and meta-regression analysis

**DOI:** 10.1097/j.pain.0000000000002883

**Published:** 2023-03-22

**Authors:** Elisabeth J. Lier, Marjan de Vries, Eline M. Steggink, Richard P.G. ten Broek, Harry van Goor

**Affiliations:** Department of Surgery, Radboud University Medical Center, Nijmegen, the Netherlands

**Keywords:** Virtual reality, Pain, Acute pain, Chronic pain, Systematic review, Meta-regression analysis

## Abstract

There is a rapidly growing body of evidence for the application of virtual reality (VR) in pain management, however, with varying effectiveness. Little is known about patient-related and VR-related factors affecting efficacy of VR. A systematic review and meta-analysis was performed including 122 randomised controlled trials (9138 patients), reporting on subjectively reported pain scores comparing an immersive VR intervention to a non-VR control group. Virtual reality significantly reduced pain in the pooled analysis (standardized mean difference = −0.65, 95% CI −0.76 to −0.54, *P* < 0.001). Subgroup analyses showed no significant differences between type of pain, ie, VR effects were similar in acute, chronic, and procedural pain conditions. Univariate and multivariate meta-regression analyses were performed to investigate the effect of intervention, patient, and pain characteristics on VR. Virtual reality effectively reduced pain, especially in patients reporting moderate to severe pain and in younger subjects. Studies comparing VR with a control group receiving no distraction methods were associated with higher effect sizes. The effect of VR was not related to a specific frequency or duration of use. Type of software and interaction level were related to VR effects in the univariable, but not in the multivariable, meta-regression analysis. Heterogeneity was considerable for all meta-analyses, and risk of bias was moderate to high in most included studies. Studies on mechanisms behind VR analgesia in younger patients and patients reporting moderate to severe pain are recommended to confirm our hypotheses while taking into account risk of bias and the comparator. Optimal application of VR using treatment modules for long-term pain conditions are an important issue for future research.

## Abstract 2

Supplemental Digital Content is Available in the Text.

## 1. Introduction

Virtual reality (VR) is increasingly used in pain management strategies for various pain conditions. Virtual reality holds promise as an attractive tool to reduce chronic analgesics use due to the low frequency and impact of side effects compared with traditional analgesics.^14,15,38,41^ In the context of the worldwide opioid crisis, VR may contribute to improved quality of life and self-efficacy of patients with chronic pain by nonpharmacological means.^2,9,14,15,25,30,38,46^ Early VR applications have mainly made use of distraction methods, ie, by immersing the subject inside a computer-generated world and thus distracting attention away from pain.^32^ Depending on the type of application, VR can also be used as a pain education tool or as an intervention with integrated psychological therapies, with effects that may last beyond the application period.

Virtual reality for pain management seems effective; however, studies vary in design, outcome measures, VR applications, and population. In addition, it remains unclear which patient characteristics, pain conditions, VR treatment aspects, and context factors contribute to the effect of VR. In contrast to drug treatments, for which the mechanism of action is roughly similar between patients, the effect of digital treatments such as VR depends more on individual physical, mental, and cognitive characteristics, and environmental factors.^39^ Previous studies mainly focus on effect sizes in specific patient populations, such as children or adults,^24,30,38^ or specific pain conditions including burn wound care or procedural pain in general.^1,2,5,9,15,18,24,25,30,33,38^ The evidence on potential effect modifiers is limited. Some previous studies have suggested that younger patients might benefit more from VR; however, results are conflicting.^11,23,37^ Another important potential effect modifier is the type of VR software used because different applications can vary substantially in interactivity and content provided.^8,13,22,35,36,45,48^ The relatively small studies available provide only limited insights into potentially modifying factors in a specific patient population. A systematic review of studies including a pooled analysis, with a multifactorial design, focusing on multiple effect modifiers, might contribute to a better understanding of how VR can be personalized and implemented in pain management.

The aim of this systematic review and meta-regression analysis was to identify potential effect modifiers related to intervention, patient, and pain characteristics and detect those patients who are most eligible for use of VR in pain management.

## 2. Methods

The review protocol of this systematic review and meta-(regression) analysis has been registered on PROSPERO (CRD42020178930). We adhered to the Preferred Reporting Items for Systematic Reviews and Meta-Analyses (PRISMA) guidelines in reporting the results.

### 2.1. Search strategy

We searched PubMed, Embase, CENTRAL, the WHO trial registry, the International Clinical Trials Registry Platform (clinicaltrials.gov), and the Science Citation Index for articles up to December 1, 2021. We used search terms, synonyms, MeSH terms, or Embase subject headings (Emtree) for “Virtual Reality” and “Pain” in title, abstract, and keywords. In addition, we searched in reference lists of included studies and previous reviews for studies that were not included in our search. The full search strategy can be found in Appendix 1: Search Strategy (available as supplemental digital content at http://links.lww.com/PAIN/B788). Deduplication was performed in EndNote X9 (Clarivate Analytics, Philadelphia, PA).

### 2.2. Study selection

Study selection was performed by 2 independent reviewers (E.J.L. and E.M.S.), first on title and abstract and in a second round on full text of selected studies. Discrepancies were discussed with a third researcher (M.d.V.) until consensus was reached. Inclusion criteria were randomised controlled trials (RCT) with a parallel group design or cross-over design, reporting on the effect of immersive VR on self-reported pain scores compared with a non-VR control group. Immersive VR was defined as any intervention using a head-mounted device (HMD) that completely blocks the view of the users' real-world surroundings. This is in contrast to nonimmersive hardware such as tablets, screens, and glasses that allow the user to stay aware and keep control of their physical environment. Exclusion criteria were (1) studies using experimental pain stimuli in volunteers; (2) studies with no control group, VR control group, or a “nonrepresentative” control group (eg, patients vs healthy volunteers); (3) studies reporting no pain-related outcome measure or an outcome that is not self-reported by the patient (eg, observational pain scores or physiological measurements on pain) or outcome measure not specified for VR users vs nonusers; (4) studies using an intervention not meeting our definition of immersive VR; (5) publications other than randomised controlled studies; and (6) full text or outcome data not available. In case of different publications by the same group using overlapping cohorts, the most recent study was included. In case of conference abstracts or study registries with complete enrollment, we searched for corresponding full-text articles by searching on title, registration number, and authors separately. When no corresponding study was found, authors were approached by email to verify whether the results of the study were published in a journal. If full text was not available, the study was excluded. No language restrictions were applied.

### 2.3. Data extraction

Two reviewers independently extracted the data from included studies (E.J.L. and E.M.S.), and discrepancies were resolved by re-examination of the study with a third researcher (M.d.V.). We extracted information on first author name, year of publication, study design, number of groups, number of participants (per group), the type of control group, and country. For outcomes, mean or median values, measures of spread (SD, standard error [SE], or interquartile range [IQR]), and type of outcome were extracted. Furthermore, we extracted details on intervention, patient, and pain characteristics for regression analyses (refer below). If data on key variables, that is, outcome measures or number of participants, were missing, the corresponding author was contacted. If the missing information could not be provided, the study was excluded.

### 2.4. Risk of bias

Risk of bias was assessed by 2 independent reviewers (E.J.L. and E.M.S.), using the Cochrane Risk of Bias 2.0 tool for parallel studies and cross-over trials. According to this tool, studies were assessed on bias arising from the randomisation process, period and carry over effects (in cross-over studies), deviation from the intervention, missing outcomes, outcome measurements, and reporting bias. Publication bias was assessed with a funnel plot, and the Egger test was used to test for funnel plot asymmetry.

### 2.5. Data preparation

The primary outcome of interest was the standardized mean difference (SMD) in pain scores in the intervention group compared with the control group. Standardized mean difference was chosen to correct for the differences in pain scales used. If mean and SD could not be retrieved to calculate SMD, studies reporting medians were included as if they reported mean values, and SDs were approximated from reported measures of spread. If no SD could be approximated, the largest SD of existing data was imputed. One study seemed as an extreme outlier compared with other included studies, and reconstruction of the study data suggested incorrect measures of spread that could not be verified with the authors; therefore, we decided to impute the largest SD of existing data.^16^ In studies with more than one comparison, intervention groups not related to VR or control were excluded. Studies comparing 2 VR intervention groups vs a control group were included as 2 separate studies; the control group was included twice. In studies with more than one VR session or outcome measurement, the measurement closest to the end of the VR therapy was extracted. In studies reporting more than one outcome, VAS (visual analogue scale) pain scores were extracted.

### 2.6. Data analysis

A random-effects meta-analysis model was used for the pooled analysis because we expected high heterogeneity between studies. Standardized mean differences of all studies were plotted in forest plots. Heterogeneity was measured by I^2^ tests; an I^2^ value greater than 75% was considered substantial heterogeneity.^20^ Sensitivity analyses were performed to explore the impact of studies of which the control group was included twice, studies with imputed SDs, studies reporting medians instead of means, studies with a high risk of bias, and studies reporting worst pain scores instead of average pain scores. A mixed-effects subgroup analysis (random-effects model within subgroups and fixed-effects [plural] model between subgroups) was performed for study design, separating parallel design and cross-over studies. A second subgroup analysis was performed for type of pain to analyse differences between acute pain, chronic pain (pain >3 months), and procedural pain (short-term pain experience arising from tissue injury associated with diagnostic or treatment procedures).

To explore potential effect modifiers, univariate meta-regression analyses (mixed-effects model) were conducted including prespecified covariates related to VR intervention, patient, and pain characteristics. We also assessed some study characteristics to check whether such factors interacted with VR efficacy. Categories were a priori developed in such a way that each subcategory would contain at least 10 studies.

Regarding features of the VR intervention, we analysed the frequency of intervention (1 session or more than one session), duration of intervention and total time in VR (in minutes), and VR software type. For type of software, the studies were divided into 5 subcategories. First, two-dimensional (2D) videos included, for example, cartoon animations. In 3D videos, the user was guided through the video, whereas in a 3D 360° VR environment, the user was able to choose the directions of the views and movements through the digital environment. Virtual reality games were defined as a digital environment in which a competitive element was incorporated. Virtual reality therapies included applications with some kind of (psychological) therapy integrated in VR, including patient education, hypnosis, and physiotherapy. Applications were marked as “interactive” if the user could interact with the digital environment, eg, by actively changing elements of the virtual world, apart from exploring the digital environment by changing the point of view.

For patient characteristics, we analysed mean age, age range of participants (subcategorised as children [0-12 year], adolescents [12-21 year], and adults [>22 year]), the percentage of male (<40%, 40%-60%, or ≥60%), type of patient (inpatient or outpatient subjects), and diagnosis. For diagnosis, we explored whether different indications for VR analgesia would affect VR efficacy, including dental care procedures (dental extractions and dental scaling), wound care (both dressing changes and physiotherapy), vascular access (venipuncture and intravenous cannulation), and studies not matching with one of these subcategories marked as “other” diagnoses.

Regarding pain characteristics, we analysed the pain score of the control group (<4 or ≥4) and the use of coanalgesia (unknown or no or nonopioids [such as acetaminophen or topical anaesthesia] or opioids [ie, ≥75% of participants using opioids]).

For study characteristics, we analysed year of publication (categorized as 2000-2010, 2011-2015, 2016-2020, or 2021) and number of participants (categorized as <50, 51-100, and >100). To investigate whether sociocultural, geographical, and socioeconomical differences would affect efficacy of VR, we also explored Continent and World bank income class (low-lower middle, upper middle, or high) of the country where the study was conducted. Finally, we categorized the type of control intervention into standard of care or no distraction, other distraction methods, or standard therapy.

Potential effect modifiers in univariate meta-regression analyses (*P* < 0.20) were included into an explorative multivariate model, together with significant variables in subgroup and sensitivity analyses. Analysis was performed using a stepwise backward selection, excluding variables until all variables in the model remained significant (*P* < 0.05).

Analyses were performed in R statistics 4.0.2 (2020-06-22), using R studio 1.3.1093.0 and packages meta, metafor, dmetar, and ggplot2.

## 3. Results

### 3.1. Search results

**Figure 1.**
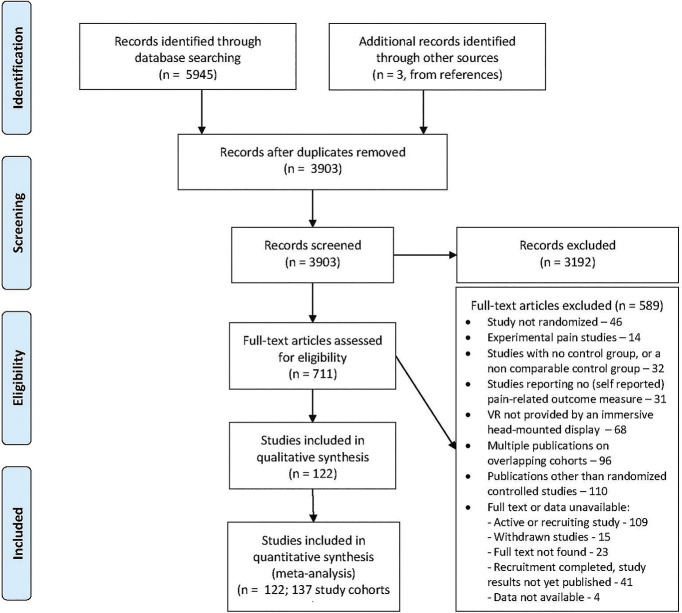
PRISMA flow diagram of included and excluded studies. PRISMA, Preferred Reporting Items for Systematic Reviews and Meta-Analyses; VR, virtual reality.

A total of 5945 citations were retrieved from the search, and 3 records were identified from references. After deduplication and screening for title and abstract, 711 articles were included for full-text screening. After excluding 589 articles that did not meet the eligibility criteria, 122 articles were included, corresponding with 137 study cohorts. Four articles were excluded during data extraction because outcome data were not available for these citations. The results of search and screening are summarized in Figure 1. Search results per database can be found in Appendix 1: Search Strategy (available as supplemental digital content at http://links.lww.com/PAIN/B788).

### 3.2. Study characteristics

Included studies were published between 2000 and 2021. A total of 9138 patients were included, with 4596 patients in the control group and 4542 in the VR intervention group. One hundred cohorts were parallel cohorts and 37 had a cross-over study design. Most study cohorts (n = 100) investigated the effect of VR on procedural pain, 14 studied chronic pain, and 23 studied acute pain conditions. The characteristics of VR interventions varied widely across studies, especially for the type of software used. Twenty-two study cohorts used applications with 2D videos and 26 cohorts used 3D video applications. Thirty study cohorts used a 3D 360° VR environment and 35 cohorts applied VR game applications. Twenty-four studies were classified as VR therapy, using hypnotherapy, physiotherapy, or patient education integrated in VR. The duration of VR interventions varied from 2 to 120 minutes, with a median duration of 10 minutes per session (IQR 5-20 minutes). In 118 study cohorts, a single VR session was used, and in 19 studies, multiple sessions were applied, with a maximum of 64 prescribed sessions. Forty-three study cohorts reported that none of the participants received any pain medication; another 47 study cohorts reported that participants received nonopioids; and in 22 study cohorts, ≥75% of participants received opioids. Twenty-five studies did not specify the use of analgesia. A summary of main study characteristics and references of included studies are provided in Appendix 2 (available as supplemental digital content at http://links.lww.com/PAIN/B788).

### 3.3. Risk of bias within studies

A summary of risk of bias assessments is provided in Appendix 3 (available as supplemental digital content at http://links.lww.com/PAIN/B788). In the overall risk of bias assessment, 12 studies (9.8%) were judged as low risk of bias, 82 (67.2%) as some concerns (moderate risk), and 28 (22.9%) as high risk of bias. There were some concerns about the randomisation process in 59 studies (48.3%), mostly because authors reported a general statement of randomisation, without specifications on randomisation or allocation concealment. Four studies (3.2%) had a high risk of bias in this domain because they did not properly randomise groups. In cross-over studies, 14 of 30 studies (46.7%) had a high risk of bias because of a short wash-out period, ie, both intervention and control were assessed in the same session within minutes after each other (Domain S: Bias arising from period and carry-over effects). Most studies (74.5%) scored “some concerns” in measurement of the outcome because both participants and outcome assessors were not blinded owing to the nature of the intervention.

Both the funnel plot (Appendix 3, available as supplemental digital content at http://links.lww.com/PAIN/B788) and the result of Egger test (*P* < 0.01) indicated that bias existed in the publication of studies; studies reporting larger effect sizes in favour of the control group were lacking.

### 3.4. Effect of virtual reality on pain

The pooled effect of 137 study cohorts showed a significant pain reduction in favour of VR (SMD = −0.65 95% CI −0.76 to −0.54, *P* < 0.001) (Appendix 4, available as supplemental digital content at http://links.lww.com/PAIN/B788), corresponding with a medium to large effect.^6^ The I^2^ value of 84.1% indicated considerable heterogeneity. The results were robust throughout sensitivity analyses (Appendix 5, available as supplemental digital content at http://links.lww.com/PAIN/B788). A subgroup analysis to account for study design did not show a significant difference between the effect of VR in cross-over or parallel design studies (SMD = −0.65 [−0.81; −0.48] and SMD = −0.65 [−0.79; −0.52] *P* = 0.95, respectively). A second subgroup analysis to account for type of pain revealed larger effect sizes for chronic pain conditions (SMD = −0.88 [−1.30; −0.46]) compared with acute pain conditions (SMD = −0.53 [−0.78; −0.78]) and procedural pain conditions (SMD = −0.65 [−0.78; −0.52]). However, the difference between subgroups was not significant, indicating that VR effects were similar in acute, chronic, and procedural pain conditions (*P* = 0.35) (Appendix 6, available as supplemental digital content at http://links.lww.com/PAIN/B788). Because other studies also consider acute and procedural pain as 1 subtype of pain, we repeated the analysis for chronic pain compared with acute (procedural) pain conditions; however, this did not change the results {acute [procedural] pain conditions (SMD = −0.62 [−0.74; −0.51])} vs chronic pain conditions (SMD = −0.88 [−1.30; −0.46], *P* = 0.25).

### 3.5. Potential effect modifiers in virtual reality pain management

Univariate meta-regression analyses revealed 8 potential effect modifiers (*P* < 0.20). Full results and bubble plots of significant variables are shown in Appendix 7, available as supplemental digital content at http://links.lww.com/PAIN/B788.

For effect modifiers related to the intervention, type of software seemed to be a potential effect modifier. The largest effects were observed in studies using 2D and 3D videos, followed by 3D 360° VR environments. Results of studies using VR games or VR therapies suggested no differences in effect sizes compared with control groups. Overall, larger analgesic effects were observed in studies using software without interactivity with the user. No differences in effect modification were shown for frequency and duration of VR sessions and total time in VR.

For patient-related potential effect modifiers, mean age of participants as a continuous variable was observed as a potential effect modification. No potential effect modification was observed for age categories, ie, children, adolescents, and adults. Furthermore, results suggested no differences in effect modification for the percentage of male participants and between type of patients (inpatients and outpatients, *P* < 0.20). Type of diagnosis was also not found to be a significant effect modifier, ie, no differences were observed in VR efficacy between dental care procedures, vascular access procedures, wound care, and other indications for VR analgesia.

For pain characteristics, pain score in the control group was found to be a significant potential effect modifier. No potential effect modification was observed for the use of coanalgesia, including the use of opioids.

Regarding study-related potential effect modifiers, no potential effect modifiers were observed for year of publication and number of participants. We did find significant effect modification for type of control group, continent, and World Bank income class.

After Bonferroni correction for multiple testing (0.05/17; *P* = 0.0029), the variables indicated as potential effect modifiers, except mean age and type of software, remained significant.

In a multivariate meta-regression model, 3 variables remained significant (*P* < 0.01) and accounted for 20.05% of the heterogeneity (residual heterogeneity 83.10%, *P* < 0.01): type of control group, mean age of participants, and pain score of the control group.

Results on pain score in the control group were similar to univariate regression analysis; a larger analgesic effect was found in patients reporting pain scores ≥4. Regarding mean age of participants, the largest VR effects were observed in younger participants. For type of control group, studies comparing VR with control groups receiving standard of care or no distraction, or control groups receiving standard therapy were associated with larger effect sizes; the smallest effect sizes were observed in studies comparing VR with other distraction methods (tablets, monitors, and toys). Type of patient, continent, World Bank income class, type of software, and interactivity level were excluded from the model by backward selection (*P* > 0.05); these variables did not significantly interact with efficacy of VR in our multivariate meta-regression model. Table 1 shows estimates and significance of covariates in univariate and multivariate analyses.

**Table 1:** Variables included in multivariate meta-regression analysis, including estimates and *P* values of univariate and multivariate analyses.

Variable	Univariate analysis		Multivariate analysis	
	Estimates (SE)*	*P*	Estimates (SE)*	*P*
Type of software 2D video 3D video 3D VR environment 3D VR game VR therapy	−0.8773 (0.13) −0.8274 (0.19) −0.6822 (0.18) −0.4723 (0.18) −0.4434 (0.19)	0.04	−0.8534 (0.24) −0.9856 (0.27) −1.1625 (0.30) −1.1660 (0.32) −1.0652 (0.32)	0.72
Interactiveness No Yes	−0.7715 (0.07) −0.4842 (0.11)	0.01	−0.8938 (0.23) −0.7896 (0.25)	0.43
Mean age of participants Per year of age	−0.8328 (0.10) 0.0067 (0.00)	0.02	**0.0095 (0.00)**	**<0.01**
Type of patients Inpatients Outpatients	−0.5521 (0.08) −0.7396 (0.11)	0.09	−0.6267 (0.16) −0.8524 (0.13)	0.07
Pain score in the control group <4 >4	−0.4395 (0.08) −0.8154 (0.11)	<0.01	**−0.8083 (0.13)** **−1.1573 (0.12)**	**<0.01**
Continent Asia Europe North America Oceania	−0.9628 (0.08) −0.2860 (0.17) −0.4382 (0.12) −0.2483 (0.21)	<0.01	−0.8616 (0.24) −0.6807 (0.41) −0.8585 (0.41) −0.6765 (0.45)	0.74
World Bank income class Low-lower middle Upper middle High	−0.9952 (0.16) −0.9911 (0.19) −0.3710 (0.18)	<0.01	−0.8757 (0.23) −0.7575 (0.17) −0.3935 (0.16)	0.29
Type of control group Standard of care or no distraction Distraction and standard of care Standard therapy	−0.7580 (0.06) −0.2433 (0.15) −0.5404 (0.20)	<0.01	**−0.8083 (0.13)** **−0.2526 (0.16)** **−0.8259 (0.24)**	**<0.01**

* Values representing regression estimates corrected for intercept. Bold indicates significant values and p-values.

SE, standard error; VR, virtual reality

## 4. Discussion

In this comprehensive evaluation of literature on VR interventions in pain management, we included evidence of 122 RCTs with 9138 patients. Virtual reality is an effective therapy to reduce pain in acute, chronic, and procedural pain conditions, with a medium to large benefit of VR with a SMD of −0.65. We found considerable heterogeneity and risk of bias in the analyses. Effect sizes were small in studies using other distraction methods as an active comparator. There is an additional gain of VR in patients reporting higher pain scores and, to a smaller extent, in younger patients.

To the best of our knowledge, previous systematic reviews on VR have addressed only a small number of factors as potential modifiers. As mentioned before, most available literature is limited in scope and addressed only efficacy for specific settings for type of pain (ie, acute, chronic, or procedural pain), specific procedures and diagnoses (eg, burn wound care or dental procedures), or specific patient populations (ie, paediatric or adult patients). In accordance with our finding of an overall positive effect of VR on pain, previous reviews with a more narrow scope also demonstrated beneficial effects for the different conditions and populations included.^1,2,5,9,15,25,29,33,37,38^

We found a marginal effect of age on the efficacy of VR. Mean age did independently affect results, showing a declining efficacy of VR with rising age. The effect per year of age was small, and categorizing age groups (child, adolescent, and adult), which is generally seen as a more reliable reflection of age distribution, did not significantly affect VR efficacy in our meta-regression analyses. Three reviews have assessed the impact of age on efficacy of VR in more depth; however, closer examination of these studies does not provide an explanation for this difference, eg, both mean age and categorized age groups have been reported as significant factors.^11,23,37^ One review with a narrow scope of paediatric patients aged 4 to 12 undergoing vascular access procedures did not observe an effect of age in their meta-regressions.^37^ A second review including both children and adults using immersive and nonimmersive VR for chronic and acute pain conditions reported larger effect sizes in juvenile patients (<18 years) compared with adults in a subgroup analysis.^23^ The third review including paediatric patients of 21 years or younger undergoing medical procedures while using a HMD found a significantly larger effect size in studies with a lower mean age of participants.^11^ In general, VR is believed to be more engaging for children, eg, due to higher levels of imagination and magical thinking in children. In the elderly patient groups, different mechanisms might affect efficacy of VR. On the one hand, prolonged wonder at the virtual environment might increase effects of VR, as supported by some experimental data.^26,27^ On the other hand, lower digital literacy among older patients can interfere with the correct application and subsequent user experience.^19^ These issues can partly be resolved by good instructions and user support, as well as applying more simple to use or passive applications in short treatments. Finally, the low number of studies in elderly populations and the relatively small number of older subjects participating in studies may have obscured older age as a modifier.

Results of the meta-analyses revealed that baseline pain score and type of comparator both had a relatively large impact on the efficacy of VR. The impact of baseline pain score is partly grounded in methodological reasons. A proportionally similar reduction in the pain score in a patient with a higher baseline pain score will result in a larger absolute pain score reduction compared with the pain score reduction in a patient with lower baseline pain score. We found a pain reduction of 25.3% (median, IQR [7.7%-44.7%] of VR compared with the control group or baseline pain in studies reporting a pain score in the control group below 4, and a clinically relevant pain reduction of 30.5% [17.6%-51.2%]) in studies reporting a pain score in the control group of 4 and above.^10,12^ Previous reviews have not investigated such differences; however, some individual studies have also observed that VR was especially effective in patients reporting severe baseline (pain score ≥7), including similar pain reductions of approximately 30% in these patients.^29,42^ In this review, the number of studies reporting severe pain scores on baseline or in the control group was too small to create a separate subgroup for severe pain to investigate whether pain reduction would further increase with more intense pain. Although the mechanism behind this larger benefit of VR is still unclear, it is encouraging to observe that VR is effective in moderate to severe pain as well.

In the analysis of type of software, we observed modest to no effects for different factors relating to the type of VR intervention applied, including type of software and interactivity. This finding was somewhat unexpected given our hypotheses and results of laboratory studies. In VR, we expected larger efficacy in studies using software with high levels of immersion and interactivity, which has previously been reported in several laboratory studies using an experimentally induced pain stimulus.^8,22,27,45^ In clinical studies, however, evidence is contradicting, with some studies reporting larger effects in interactive VR compared with passive VR, or virtual environments compared with videos, but other studies found similar effects in different types of software.^13,35,36,48^ A potential explanation for the limited added value of interactive computer-generated environments as opposed to passive VR software applications in this review might be found in the relatively large number of studies on periprocedural pain using an intervention providing video content by a HMD. Although the type of pain was also included in the multivariate regression analysis, the number of studies investigating chronic pain conditions was small, as was the number of studies investigating therapeutic modules integrated in VR. This review did not demonstrate a major impact of these applications; however, this subgroup included a heterogeneous collection of therapies and the therapies have been used for a variety of pain conditions, complicating further analyses. It should be mentioned that the small number of studies in other subgroups also limited our ability to perform additional analyses, for example, on interaction factors that might explain some of the results. Although we expected the model to correct for most baseline differences, an interaction factor between variables affecting the results cannot be completely ruled out.

In the analysis of type of comparator we found that, although VR was effective compared with all types of comparators, the effect was much smaller when VR was compared with control groups exposed to distractive methods in addition to standard of care. We hypothesize that during short periods of pain, such as periprocedural pain, distraction might be the major component in pain relief related to VR. Based on our findings, we suggest that any VR application that provides good distraction seems effective to reduce such pain, whenever using an immersive VR device. However, mechanisms of distraction conceptually seem insufficient to achieve long-lasting results.^44^

Virtual reality is a rapidly developing technology and some of its features are still in early development. Over the past few years, many improvements have been made for hardware specifications including display quality or field of view, and more recent developments include the possibility of monitoring and interacting with heartbeat and eye tracking. These features may increase the level of immersion and interactivity and, therefore, also contribute to increased efficacy of VR.^3,21,40^ In addition, new VR software modules have been developed that may specifically address needs of patients with chronic pain. Such treatment modules can be deployed as part of pain education, providing patients with tools to cope with pain outside the periods that they are actually using the VR. Furthermore, modules can contain interactive elements that are part of pain reprocessing therapies, including aspects of cognitive behavioral therapy, EMDR therapy (Eye Movement Desensitization and Reprocessing), and mindfulness. Another aspect includes the integration of embodiment in VR therapies, through which the illusion of ownership of a virtual body is used to change body perception. Virtual embodiment combines the feeling of presence with high levels of immersion and interactivity, thereby creating a strong perceptual illusion that goes beyond distraction analgesia.^44^ Virtual embodiment using avatars has already been reported to reduce pain in experimental studies in healthy volunteers and patients with chronic neuropathic pain. Although conceptually promising, the efficacy of such modules is yet to be evaluated in studies.^31,43,44^

Some limitations and features of this meta-analysis should be discussed as they affect the interpretation of the evidence. This review provides important insights into the efficacy of VR and which characteristics might be used to select individual patients who are most eligible for use of VR in pain management. However, it should be noted that results of this review can mostly be used to generate hypotheses. An individual patient data (IPD) meta-analysis would have provided stronger evidence, especially in analysing potential effect modifiers; however, obtaining individual patient data was not feasible. Second, to generate these hypotheses on potential effect modifiers, we included a large diversity of studies, which might explain the high level of heterogeneity observed. However, the analyses performed could explain heterogeneity only partly; heterogeneity in subgroup analyses and meta-regression analyses was still of substantial or considerable level. Risk of bias was also substantial with less than 1 in 10 studies scoring as low risk of bias. Major aspects affecting the risk of bias were lack of blinding and carry-over effects that could not be ruled out. Because of the nature of the intervention, the results of this review include the effects of a nonblinded intervention. Together with self-reported outcomes, this might have introduced ascertainment bias.^4,47^ Placebo effects related to group assignment cannot be ruled out because VR cannot be provided in a blinded fashion. A placebo effect could significantly modulate treatment effects for conditions in which VR is increasingly used, including pain and anxiety.^2,5,7,17,33,38^ Nevertheless, we performed many subgroup and sensitivity analyses and the beneficial effects of VR were consistent throughout all these different analyses. Regarding carry-over effects, VR effects are believed to act shortly; however, carry-over effects could not be ruled out. In many cross-over studies, a wash-out period was only a few minutes between sessions. The lack of wash-out periods is expected to result in an underestimation of the effects of VR, which is considered a less dangerous bias than overestimation. Furthermore, there was no difference in effect size compared between parallel and cross-over studies, suggesting the impact of this potential source of bias is low.

Finally, for patients with chronic pain, outcomes related to pain intensity scores might not be the most appropriate measure to determine success of treatment because they fluctuate and are also dependent of functioning and participation. Measures of pain disability and social functioning seem to be more relevant outcome measures for these patients.^34^

In conclusion, VR significantly reduced pain in acute, chronic, and procedural pain conditions, in particular, in patients reporting moderate to severe pain and, to a smaller extent, in younger patients. To confirm our hypotheses, studies on mechanisms behind VR analgesia in these patient groups are recommended. Optimal application of VR using new VR treatment modules in the setting of prolonged use and chronic pain conditions is an important topic for future research. Study characteristics with regard to risk of bias and type of control group should be taken into account in designing VR research.

## Conflict of interest statement

The authors declare that they have no competing interests.

## Appendix A. Supplemental digital content

Supplemental digital content associated with this article can be found online at http://links.lww.com/PAIN/B788.
