# Supplementary material for: Effect modifiers of virtual reality in pain management: a systematic review and meta-regression analysis
Source: Pain. 2023 Mar 22;164(8):1658–65. doi: 10.1097/j.pain.0000000000002883 (PMC10348651; doi:10.1097/j.pain.0000000000002883)
Supplement: Supplementary file 1 [file jop-164-1658-s001.pdf]

Appendix 1: Search Strategy

P:

Pain [Tiab] OR Pain*[Tiab] OR chronic pain [Tiab] OR acute pain [Tiab] OR procedural pain [Tiab] OR pain management [Tiab] OR postoperative pain [Tiab] OR pain scores [Tiab] OR pain intensity [Tiab] OR pain measurement [Tiab] OR analgesia [Tiab] OR discomfort [Tiab] OR "Pain"[Mesh] OR "Chronic Pain"[Mesh] OR "Acute Pain"[Mesh] OR "Pain Management"[Mesh] OR "Analgesia"[Mesh] OR "Pain Measurement"[Mesh]

I:

Virtual Realit*[Tiab] OR Virtual reality therap*[Tiab] OR Virtual reality analges*[Tiab] OR Virtual reality distraction[Tiab] OR Virtual reality immersion therap*[Tiab]OR Virtual world[Tiab] OR Virtual reality exposure therap*[Tiab] OR virtuality [Tiab] OR simulated realit*[Tiab] OR head mounted display[Tiab] OR virtual reality goggles[Tiab] OR helmed[Tiab] OR goggles[Tiab] OR helmed display[Tiab] OR "Virtual Reality"[Mesh] OR "Virtual Reality Exposure Therapy"[Mesh]

C: -

O: -

Pubmed:

((Virtual Realit*[Tiab] OR Virtual reality therap*[Tiab] OR Virtual reality analges*[Tiab] OR Virtual reality distraction[Tiab] OR Virtual reality immersion therap*[Tiab]OR Virtual world[Tiab] OR Virtual reality exposure therap*[Tiab] OR virtuality [Tiab] OR simulated realit*[Tiab] OR head mounted display[Tiab] OR virtual reality goggles[Tiab] OR helmed[Tiab] OR goggles[Tiab] OR helmed display[Tiab] OR "Virtual Reality"[Mesh] OR "Virtual Reality Exposure Therapy"[Mesh])) AND (Pain [Tiab] OR Pain*[Tiab] OR chronic pain [Tiab] OR acute pain [Tiab] OR procedural pain [Tiab] OR pain management [Tiab] OR postoperative pain [Tiab] OR pain scores [Tiab] OR pain intensity [Tiab] OR pain measurement [Tiab] OR analgesia [Tiab] OR discomfort [Tiab] OR "Pain"[Mesh] OR "Chronic Pain"[Mesh] OR "Acute Pain"[Mesh] OR "Pain Management"[Mesh] OR " Analgesia"[Mesh] OR "Pain Measurement"[Mesh])

Embase:

((Pain or chronic pain or acute pain or procedural pain or pain management or postoperative pain or pain scores or pain intensity or pain measurement or analgesia or discomfort).ti,ab,kw. or exp pain severity/ or exp pain intensity/ or exp pain measurement/ or exp pain threshold/ or exp pain assessment/ or exp procedural pain/ or exp pain/ or exp chronic pain/) and ((Virtual Realit* or Virtual reality therap* or Virtual reality analges* or Virtual reality distraction or Virtual reality immersion therap* or Virtual world or Virtual reality exposure therap* or virtuality or simulated realit* or head mounted display or helmed or helmed display or goggles or virtual reality goggles).ti,ab,kw. or exp virtual reality/ or exp virtual reality exposure therapy/)

CENTRAL:

(Virtual Reality OR Virtual reality therapy OR Virtual reality analgesia OR Virtual reality distraction OR Virtual reality immersion therapy OR Virtual world OR Virtual reality exposure therapy OR virtuality OR simulated reality OR head mounted display OR helmed OR goggles OR helmed display OR virtual reality goggles) AND (Pain OR chronic pain OR acute pain OR procedural pain OR pain management OR postoperative pain OR pain scores OR pain intensity OR pain measurement OR analgesia OR discomfort)

WHO trial registry:

(Virtual Reality OR Virtual reality therapy OR Virtual reality analgesia OR Virtual reality distraction OR Virtual reality immersion therapy OR Virtual world OR Virtual reality exposure therapy OR head mounted display OR goggles) AND (Pain OR chronic pain OR acute pain OR pain OR procedural pain OR management OR postoperative pain OR pain scores OR pain intensity OR pain measurement OR analgesia OR discomfort)

Clinicaltrials.gov:

Virtual Reality OR Virtual reality therapy OR Virtual reality analgesia OR Virtual reality distraction OR Virtual reality immersion therapy OR Virtual world OR Virtual reality exposure therapy OR virtuality | Pain OR chronic pain OR acute pain OR procedural pain or pain management OR postoperative pain OR pain scores OR pain intensity OR pain measurement OR analgesia OR discomfort

Web of Science:

TS=(Pain OR chronic pain OR acute pain OR pain management OR postoperative pain OR procedural pain OR pain scores OR pain intensity OR pain measurement OR analgesia OR discomfort) AND TS=(Virtual Reality OR Virtual reality therapy OR Virtual reality analgesia OR Virtual reality distraction OR Virtual reality immersion therapy OR Virtual world OR Virtual reality exposure therapy OR virtuality OR simulated reality OR head mounted display OR helmed OR goggles OR helmed display OR virtual reality goggles)

**Suppl. Table: Search ResultsDatabase**	**Results**	**Date**
Pubmed (+Medline)	1054	01-12-2021
Embase	1929	01-12-2021
International Clinical Trials Registry Platform (clinicaltrials.gov)	319	01-12-2021
CENTRAL (The Cochrane Central Register of Controlled Trials)	373	01-12-2021
Science Citation Index, Web of science	2084	01-12-2021
WHO trial registry (apps.who.int/trialsearch/	185	01-12-2021
Total	5945	01-12-2021

Appendix 2: Summary of included articles and study cohorts.
Table 1. Characteristics of included studies

Author	Year	Design	Type of pain	Participants				Control	Intervention		Outcome included in meta-analysis
				N (EG:CG)	Diagnosis or Procedure	Mean age (yrs) (*Range)	% Male		Frequency, duration	Software	
Akin [1]	2021	Parallel	Acute	100 (50:50)	Labor pain	27.3	0.0	Standard of Care	One session, 15 min	3D video images of the foetus	VAS pain score during labor, measured after intervention
Al-Halabi [2]	2018	Parallel	Procedural	101 (33:34)	Dental care nerve block	7.4	59.4	No distraction	One session, unknown	Cartoon video	WBFPS pain score during procedure, measured after intervention
Ali [3]	2021	Parallel	Procedural	22 (11:11)	Burn wound care physiotherapy	13.2	59.1	Standard of Care	One session, 20-25 min	3D video	VAS pain score after procedure
Alshatrat [4]	2018	Crossover	Procedural	50 (50:50)	Dental treatment	36.0	44.0	No distraction	One session, 30 min	2D video documentary, comedy	Average VAS pain score during procedure, measured after intervention
Asl Aminabadi [5]	2012a	Crossover	Procedural	117 (58:58)	Dental treatment	5.2	56.9	No distraction	One session, 30 min	Cartoon video	WBFPS pain score during procedure, measured after intervention
	2012b	Crossover	Procedural	117 (59:59)	Dental treatment	5.7	50.9	No distraction	One session, 30 min	Cartoon video	WBFPS pain score during procedure, measured after intervention
Asvanund [6]	2015a	Crossover	Procedural	23 (23:23)	Dental care local injection	6.9	44.0	Standard of Care	One session, 2 min	Cartoon video	FPSR pain score after procedure
	2015b	Crossover	Procedural	21 (21:21)	Dental care local injection	7.1	35.0	Standard of Care	One session, 2 min	Cartoon video	FPSR pain score after procedure
Atzori [7]	2018a	Crossover	Procedural	15 (15:15)	Venipuncture	10.9	66.7	No distraction	One session, 3 min	VR game SnowWorld	VAS worst pain during procedure, measured after intervention
Atzori [8]	2018b	Crossover	Procedural	5 (5:5)	Dental treatment	13.2	60.0	No distraction	One session, unknown	VR game SnowWorld	GRS worst pain during procedure, measured after intervention
Austin [9]	2020	Crossover	Chronic	16 (16:16)	Spinal cord injury neuropathic pain	54.3	100.0	2D Nature trek	One session, 15 min	3D 360 environment Nature Trek	NRS average pain score during intervention, measured after intervention
Aydin [10]	2019	Parallel	Procedural	120 (60:60)	Venipuncture	10.4	50.8	No distraction	One session, 3-4 min	Interactive 360 3D VR	VAS pain score after procedure

										environment aquarium VR	
Azurdia [11]	2018	Crossover	Chronic	11 (11:11)	Spinal cord injury pain	43.3	45.5	Conventional Rehabilitation	One session, 6 min	Passive 3D 360 VR environment	NRS after intervention
Babaie [12]	2019	Parallel	Procedural	62 (32:30)	Peripheral venous catheterisation	7.75	51.6	No distraction	One session, 2 min	Animation video	OFS pain during procedure, measured 10 min after procedure
Bani Mohammad [13]	2018	Parallel	Chronic	80 (40:40)	Breast cancer pain	52.0	0.0	Standard of Care	One session, 15 min	Interactive 3D VR environment Ocean rift, Happy Place	VAS pain score after intervention
Barad [14]	2020	Parallel	Procedural	108 (36:36)	Venipuncture	7.5	48.1	No distraction	One session, 5 min	Animation video	WBFPS pain score after procedure
Basak [16]	2020	Parallel	Procedural	120 (40:40)	Peripheral venous catheterisation	34.0	40.0	No distraction	One session, 2-3 min	3D video	VAS pain score during procedure, measured after intervention
Basak [15]	2021	Parallel	Procedural	91 (31:30)	Intramuscular injection	31.1	Unknown	No distraction	One session, 5 min	3D video	VAS pain score after procedure
Bekelis [17]	2017	Parallel	Acute	127 (64:63)	Craniotomy or spinal surgery	55.3	58.1	Standard of Care	One session, 5 min	3D Video of surgical procedure	VAS pain score measured within 24h after surgery
Bentsen [18]	2001	Crossover	Procedural	23 (23:23)	Dental treatment	27.0	26.1	No distraction	One session, 2.5 min	3D video	VAS pain score after procedure
Bentsen [19]	2003	Crossover	Procedural	26 (26:26)	Dental treatment	55.0	53.9	No distraction	One session, unknown	2D music video	VAS pain score after procedure
Brown [20]	2020	Parallel	Procedural	45 (15:15)	Spinal injection	61.9	40.0	No distraction	One session, 5 min	3D Nature video	NRS pain score after procedure
Buldur [21]	2021a	Crossover	Procedural	70 (38:38)	Dental treatment	8.97	52.6	No distraction	One session, 32 min	Cartoon or animation video	WBFPS pain score after local injection, measured during procedure
	2021b	Crossover	Procedural	70 (36:34)	Dental treatment	9.07	50.0	No distraction	One session, 32 min	Cartoon or animation video	WBFPS pain score after local injection, measured during procedure
Butt [22]	2021	Parallel	Acute	110 (55:55)	Procedures on emergency department	15.0	60.0	iPad	One session, 5 min	VR meditation	WBFPS pain score after procedure
Canares [23]	2021	Parallel	Procedural	55 (19:20)	Venipuncture	14.1	36.0	No distraction	One session, 12 min	VR games or video	VAS worst pain score during procedure, measured after intervention

Carrougher [24]	2009	Crossover	Procedural	39 (39:39)	Burn wound care physiotherapy	35.0	89.7	Standard of Care	One session, 10 min	VR game SnowWorld	GRS worst pain during procedure, measured after intervention
Caruso [25]	2020	Parallel	Procedural	220 (106:114)	Venipuncture, Peripheral venous catheterisation, or port access	13.6	57.3	Distraction and Standard of Care	One session, unknown	VR games Ocean Rift, Pebbles the Penguin, Space Pups	FPSR pain score after procedure
Chan [27]	2007	Crossover	Procedural	8 (8:8)	Burn wound care	6.5	87.5	Standard of Care	One session, 20-35 min	VR game	FPS pain score during procedure, measured during intervention
Chan [26]	2019a	Parallel	Procedural	123 (64:59)	Venipuncture	8.1	54.5	Distraction and Standard of Care	One session, 7 min	Interactive 3D 360 VR environment, marine scenes	FPSR pain score during procedure, measured after intervention
	2019b	Parallel	Procedural	129 (63:66)	Venipuncture	7.8	57.4	Distraction and Standard of Care	One session, 5 min	Interactive 3D VR environment, marine scenes	FPSR pain score during procedure, measured after intervention
Chang [28]	2021	Parallel	Procedural	16 (8:8)	Laryngoscopy	59.4	60.0	Standard of Care	One session, 12 min	Passive 3D 360 VR environment	VAS pain score during procedure, measured after intervention
Chen [29]	2020	Parallel	Procedural	136 (68:68)	Intravenous injection	9.1	56.6	Standard of Care	One session, 3 min	Passive 3D 360 VR environment	WBFPS pain score during procedure, measured 5 min after intervention
Clerc [30]	2021	Parallel	Acute	64 (35:29)	Plastic surgery	11.5	42.0	Distraction and Standard of Care	One session, 22 min	3D video Rollercoaster	FPSR pain score after procedure
Darnall [31]	2020	Parallel	Chronic	74 (35:39)	Low back pain, fibromyalgia	25-74*	70.3	Audio Cognitive Behavioral Therapy program	One session on 21 days, 1-15 min/ session	VR Cognitive Behavioral Therapy program	NRS average pain score for previous 24 hours after 21 days of therapy
Das [32]	2005	Crossover	Procedural	7 (7:7)	Burn wound care	11.1	66.7	Standard of Care	One session on 1-3 days, 10-40 min/ session	VR game	FPSR average pain score during procedure, measured after intervention
De Silva [33]	2016	Parallel	Procedural	200 (67:67)	Colonoscopy	54.1	55.0	No distraction	One session, Unknown	Video; action, comedy, cartoon	VAS pain score during procedure, measured after recovery
Deo [34]	2020	Parallel	Procedural	40 (20:20)	Hysteroscopy	31.2	0.0	Standard of Care	One session, 4.1 min	Guided relaxation VR	NRS pain score during procedure, measured after intervention
Ding [35]	2019	Parallel	Procedural	182 (91:91)	Wound care after	45.8	39.6	Standard of Care	One session, 21.2 min	VR game Snowworld	VAS pain score during procedure, measured 20 min after procedure

					Hemorroid-ectomy						
Dumoulin [36]	2019	Parallel	Procedural	59 (20:15)	Venipuncture	13.4	65.0	Standard of Care	One session, 10 min	VR game	VAS pain score during procedure, measured after intervention
Ebrahimi [37]	2017	Parallel	Procedural	60 (20:20)	Burn wound care	35.0	56.7	Standard of Care	One session on 1-5 days, unknown	Passive 3D 360 VR environment	VAS pain score during procedure, measured after intervention day 5
Eijlers [38]	2019	Parallel	Acute	191 (94:97)	Dental surgery	7.9	52.9	Standard of Care	One session, 15 min	Interactive 3D 360 VR environment	FPSR postoperative pain at recovery
El-Sharkawi [39]	2012	Crossover	Procedural	42 (42:42)	Dental care local injection	5-7*	47.6	No distraction	One session, unknown	Animated movies	FPS pain after procedure
Erdogan [40]	2021	Parallel	Procedural	142 (37:34)	Venipuncture	9.4	50.0	No distraction	One session, 5 min	3D video, Dinosaur animation	VAS pain score after procedure
Felemban [41]	2021	Parallel	Procedural	50 (25:25)	Dental care local injection	8.4	42.0	Cartoon video on screen	One session, 5 min	Cartoon video via VR goggles	WBFPS pain score during procedure, measured after intervention
Fouks [42]	2021	Parallel	Procedural	82 (44:38)	Hysteroscopy	38.5	0.0	Standard of Care	One session, 7 min	Interactive 3D 360 VR environment SoothVR	NRS pain score during procedure, measured after procedure
Frey [43]	2018	Crossover	Acute	27 (27:27)	Labor pain	27.9	0.0	No distraction	One session, 10 min	Interactive 3D 360 VR environment Ocean Rift	NRS worst pain during intervention, measured after intervention
Furman [44]	2009a	Crossover	Procedural	38 (38:38)	Dental treatment	45.9	44.7	No distraction	One session, 20 min	Interactive 3D 360 VR environment	VAS average pain during procedure, measured after intervention
	2009b	Crossover	Procedural	38 (38:38)	Dental treatment	45.9	44.7	No distraction	One session, 20 min	3D video Cars	VAS average pain during procedure, measured after intervention
Gershon [46]	2004	Parallel	Procedural	59 (22:22)	Port acces	12.7	51.0	No distraction	One session, 10-15 min	VR game Virtual Gorilla program	VAS pain during procedure, measured after intervention
Gold [47]	2006	Parallel	Procedural	20 (10:10)	Peripheral venous catheterisation	10.2	60.0	No distraction	One session, 10 min	VR game Street Luge	FPSR pain score after procedure
Gold [48]	2018	Parallel	Procedural	143 (70:73)	Venipuncture	15.4	49.7	Standard of Care	One session, 5 min	VR game Bear Blast	VAS pain score after procedure
Gold [49]	2021	Parallel	Procedural	107 (53:54)	Peripheral venous catheterisation	14.7	58.9	Distraction and Standard of Care	One session, 5 min	VR game Bear Blast	FPSR pain score after procedure

Goldman [51]	2021a	Parallel	Procedural	66 (35:31)	Peripheral venous catheterisation	9.5	55.0	Distraction and Standard of Care	One session, 15 min	3D video Rollercoaster	FPSR pain score after procedure
Goldman [50]	2021b	Parallel	Procedural	62 (32:30)	Laceration repair	10.2	63.0	Distraction and Standard of Care	One session, 30 min	3D video Rollercoaster	FPSR pain score after procedure
Gray [52]	2021a	Crossover	Procedural	86 (43:43)	Nasal endoscopy	45.5	53.7	Standard of Care	One session, 5 min	VR game SpaceBurgers	VAS pain during procedure, measured after procedure
	2021b	Crossover	Procedural	78 (39:39)	Nasal endoscopy	45.5	53.7	Standard of Care	One session, 5 min	VR game SpaceBurgers	VAS pain during procedure, measured after procedure
Groninger [53]	2021	Parallel	Acute	88 (52:36)	Heart failure pain	56.0	61.2	2D guided imagery	One session, 10 min	VR meditation	NRS pain score after intervention
Gulsen [54]	2020	Parallel	Chronic	16 (8:8)	Fibromyalgia	42.5	0.0	Conventional Rehabilitation	Two sessions a week for 8 weeks, 20 min/session	VR physiotherapy game	VAS pain score after 8 weeks of therapy
Guo [55]	2014	Parallel	Procedural	98 (49:49)	Dressing change of hand wounds	31.1	86.7	No distraction	One session, unknown	3D video	VAS pain score during procedure, measured after intervention
Gür [56]	2020a	Parallel	Acute	273 (54:54)	Labor pain	26.0	0.0	Standard of Care	One session, 10 min	2D video of newborns with classical music	VAS pain score after intervention
	2020b	Parallel	Acute	273 (55:54)	Labor pain	26.0	0.0	Standard of Care	One session, 10 min	2D video of newborns	VAS pain score after intervention
	2020c	Parallel	Acute	273 (55:54)	Labor pain	26.0	0.0	Standard of Care	One session, 10 min	2D video of Turkey	VAS pain score after intervention
Haisley [57]	2020	Parallel	Acute	52 (26:26)	Foregut surgery	64.5	26.9	Standard of Care	Two perioperative sessions, 13-15 min / session	VR meditation	NRS pain score day after surgery
Hassannia [58]	2021	Parallel	Acute	40 (20:20)	Circumcision surgery	6.7	100.0	No distraction	One session	VR video of procedure, 360 animation	OPS pain score after surgery
Hoffman [59]	2000	Crossover	Procedural	12 (12:12)	Burn wound care physiotherapy	27.7	91.7	No distraction	One session, 3 min	Interactive 3D 360 VR environment SpiderWorld	VAS average pain during procedure, measured after intervention
Hoffman [60]	2001	Crossover	Procedural	7 (7:7)	Burn wound care physiotherapy	21.9	85.7	Standard of Care	One session on 3-7 days, 3-9 min / session	VR game SnowWorld	VAS average pain during procedure, measured after intervention on day 7

Hoffman [61]	2008	Crossover	Procedural	11 (11:11)	Burn wound care	27.0	100.0	No distraction	One session, 3 min	VR game SnowWorld	GRS worst pain during procedure, measured after intervention
Hoxhallari [62]	2019	Parallel	Acute	41 (21:20)	Hand surgery	53.0	43.9	Standard of Care	One session, 8-35 min	3D VR Youtube videos	NRS pain during surgery, measured after procedure
Hua [63]	2015	Parallel	Procedural	65 (33:32)	Wound dressing of chronic wounds	8.7	47.7	Video game on laptop	One session, 32 min	VR game	VAS pain score during procedure, measured during intervention
Hwang [64]	2014	Parallel	Chronic	39 (13:13)	Complex regional pain syndrome	40.8	71.7	Mental Rehersal voice recording	One session, 6 min	Virtual body swapping	NRS pain intensity after intervention
Inangil [65]	2020	Parallel	Procedural	120 (40:40)	Venipuncture	9.1	55.0	No distraction	One session, 4 min	Cartoon video	WBFPS pain score due to procedure, measured after intervention
JahaniShoorab [66]	2015	Parallel	Procedural	30 (15:15)	Episiotomy repair	24.1	0.0	Standard of Care	One session, 11 min	3D video IMAX Dolpine and Whales 3D	NRS Pain score during procedure, measured during second stage of repair
Jeffs [67]	2014	Parallel	Procedural	28 (8:10)	Burn wound care	13.5	68.0	Standard of Care	One session, 5-100 min	VR game SnowWorld	APPT WGRS pain score during procedure, measured after intervention
Jin [69]	2016	Crossover	Chronic	66 (33:33)	Chronic pain conditions	30-75*	20.0	Self-mediated control	One session, 10 min	VR game Cryoslide	VAS Pain intensity during intervention, measured after intervention
Jin [68]	2018	Parallel	Acute	20 (20:20)	Total Knee arthroplasty	66.4	42.4	Conventional Rehabilitation	Three sessions a day until discharge, 30 min / session	Interactive 3D 360 VR environment including rehabilitation	VAS pain intensity 7 days after surgery
Joo [70]	2021	Parallel	Procedural	38 (19:19)	Spinal injection	62.6	42.1	No distraction	One session, 30 min	VR meditation	NRS pain during procedure, measured after procedure
Karaman [71]	2021	Parallel	Procedural	60 (30:30)	Breast biopsy	44.0	0.0	Standard of Care	One session, 5 min	Passive 3D 360 VR environment Walk on the beach	VAS pain score during procedure, measured after procedure
Karaveli Çakır [72]	2021	Parallel	Procedural	60 (30:30)	Colonoscopy	56.0	65.0	Standard of Care	One session, 10 min	3D VR images	VAS pain score during procedure, measured after procedure

Kipping [73]	2012	Parallel	Procedural	41 (20:21)	Burn wound care	13.1	68.3	Distraction and standard of care	One session, 18 min (4-120)	VR game Chicken Little	VAS pain score during second stage of procedure (application), measured after intervention
Koc Özkan [74]	2019	Parallel	Procedural	135 (46:43)	Venipuncture	9.3	33.3	Standard of Care	One session, unknown	Video	VAS pain score after procedure
Konstantatos [75]	2009	Parallel	Procedural	86 (43:43)	Burn wound care	38.6	Unknown	Standard of Care	One session, 18 min	VR Hypnotherapy	VAS Worst pain during procedure, measured after intervention
Koushali [76]	2017	Parallel	Procedural	40 (20:20)	Burn wound care	9.0	65.0	Standard of Care	One session, < 30-40 min	VR game Smash Hit	WBFPS after procedure
Laghlam [77]	2021	Parallel	Procedural	180 (90:90)	Drain removal	68.0	75.0	Standard of Care	One session, 15 min	3D 360 VR environment	NRS pain score after procedure
Le May [78]	2021	Crossover	Procedural	20 (20:20)	Burn wounds or orthopedic injuries	11.8	75.0	Standard of Care	One session, 15 min	VR game Dreamland	NRS pain score after procedure
Litwin [79]	2021	Parallel	Procedural	58 (31:27)	Peripheral venous catheterisation	12.5	56.9	2D video on screen	One session, 4 min	Interactive 3D 360 VR environment KidsVR aqua	NRS pain score after procedure
Liu [80]	2020	Parallel	Procedural	53 (30:23)	Nasal endoscopy	12.5	69.8	Standard of Care	One session, 2 min	VR game SpaceBurgers	WBFPS pain score during procedure, measured after procedure
Luczak [81]	2021	Parallel	Procedural	103 (52:51)	Cystoscopy	66.4	71.8	Standard of Care	One session, unknown	Passive 3D 360 VR environment	NRS pain score after procedure
Maani [82]	2011	Crossover	Procedural	12 (12:12)	Burn wound care	22.0	100.0	Standard of Care	One session, 6 min (1-11)	VR game SnowWorld	GRS worst pain during procedure, measured after intervention
McSherry [83]	2018	Crossover	Procedural	12 (12:12)	Burn wound care	38.4	72.0	Standard of Care	One session, 30 min	VR game SnowWorld	NRS pain after procedure
Melcer [84]	2021	Parallel	Procedural	60 (30:30)	Amniocentesis	35.8	0.0	Standard of Care	One session, 5-15 min	3D video	VAS pain score during procedure, measured after procedure
Mitrakul [85]	2015a	Crossover	Procedural	42 (21:21)	Dental treatment	6.8	48.0	No distraction	One session, 30 min	Cartoon video	FPSR pain score during procedure, measured after procedure
	2015b	Crossover	Procedural	42 (21:21)	Dental treatment	7.0	29.0	No distraction	One session, 30 min	Cartoon video	FPSR pain score during procedure, measured after procedure
Mohanasundari [86]	2021	Parallel	Procedural	105 (35:35)	Venipuncture, Peripheral venous catheterisation	56.1	8.2	Standard of Care	One session, 15 min	Cartoon video	WBFPS pain score during procedure, measured after procedure

Momenyan [87]	2021	Parallel	Acute	52 (26:26)	Labor pain	29.4	0.0	Standard of Care	Two sessions, 10 min/session	Passive 3D 360 VR environment	VAS pain score after intervention
Morales Tejera [112]	2020	Parallel	Chronic	44 (22:22)	Neck pain	48.0	29.7	Standardized fysiotherapy	Eight sessions, Unknown	VR therapy exercises	VAS pain score after 4 weeks of treatment
Morris [88]	2010	Crossover	Procedural	11 (11:11)	Burn wound care physiotherapy	33.0	72.7	Standard of Care	One session, 9 min (7.5-10)	VR game Chicken Little	NRS pain after procedure
Mukherjee [89]	2020	Parallel	Chronic	43 (22:21)	Neck pain	55.0	47.7	Standardized fysiotherapy	Three sessions, 10 min/ session	VR therapy exercises	NRS pain score after three days of treatment
Niharika [90]	2018a	Crossover	Procedural	36 (18:18)	Dental treatment	7.2	44.4	Standard of Care	One session, 30 min	Cartoon video Doreman	WBFPS pain score during procedure, measured after procedure
	2018b	Crossover	Procedural	36 (18:18)	Dental treatment	7.3	55.6	Standard of Care	One session, 30 min	Cartoon video Doreman	WBFPS pain score during procedure, measured after procedure
Nunna [91]	2019	Parallel	Procedural	70 (35:35)	Dental care local injection	8.9	50.0	Counter stimulation	One session, unknown	3D cartoon video	VAS pain score after procedure
Nusser [92]	2021	Parallel	Chronic	55 (17:18)	Neck pain	51.3	62.7	Standardized fysiotherapy	Six sessions, 20 min/ session	VR therapy exercises	NRS pain score after 4 weeks of treatment
Osmanlliu [93]	2020	Parallel	Procedural	62 (31:31)	Venipuncture, Peripheral venous catheterisation	11.7	38.7	Distraction	One session, 3 min	VR game Dreamland	NRS pain score during procedure, measured after procedure
Ozalp Gerceker [95]	2018	Parallel	Procedural	121 (40:40)	Venipuncture	9.4	50.4	No distraction	One session, unknown	Animation video	WBFPS pain score after procedure, measured within 5 min after intervention
Ozalp Gerceker [94]	2020a	Parallel	Procedural	136 (45:46)	Venipuncture	5-12*	53.8	No distraction	One session, unknown	3D video Rollercoaster	WBFPS pain score after procedure
	2020b	Parallel	Procedural	136 (45:46)	Venipuncture	5-12*	53.8	No distraction	One session, unknown	Interactive 3D 360 VR environment Ocean Rift	WBFPS pain score after procedure
Ozalp Gerceker [45]	2021	Parallel	Procedural	42 (21:21)	Port access	11.5	61.9	Standard of Care	One session, 5 min	Interactive 3D 360 VR environment Ocean Rift, Rollercoaster video	WBFPS pain score after procedure

Powers [96]	2021a	Parallel	Acute	103 (34:34)	Pain in hospitalised patients	42.8	56.0	No distraction	One session, 10 min	Passive 3D 360 Video environment	NRS pain score after intervention
	2021b	Parallel	Acute	103 (35:34)	Pain in hospitalised patients	42.2	60.9	No distraction	One session, 10 min	Passive 3D 360 virtual environment	NRS pain score after intervention
Ran [97]	2021	Parallel	Procedural	120 (60:60)	Dental care, local injection	5.6	52.5	Standard of Care	One session, 19 min	Passive 3D 360 VR environment	WBFPS pain score after injection
Rousseaux [98]	2021a	Parallel	Acute	70 (15:22)	Cardiac surgery	64.0	74.0	Standard of Care	Two sessions, 20 min/ session	Passive 3D 360 VR environment	VAS pain score after second session
	2021b	Parallel	Acute	70 (15:22)	Cardiac surgery	65.8	78.0	Standard of Care	Two sessions, 20 min/ session	VR hypnosis therapy	VAS pain score after second session
Sander Wint [99]	2002	Parallel	Procedural	30 (17:13)	Lumbal puncture	13.6	53.3	Standard of Care	One session, 32 min	3D video Escape	VAS pain score during procedure, measured 30min after intervention
Sarig Bahat [101]	2015	Parallel	Chronic	90 (30:30)	Neck pain	40.9	34.4	Standard physiotherapy	Four till six sessions in 5 weeks, 15-20 min/ session	VR therapy exercises	VAS pain score after 5 weeks of therapy
Sarig Bahat [100]	2017	Parallel	Chronic	30 (16:14)	Neck pain	48.0	30.0	No VR, waiting list	Four times a day, 4 days a week, for 4 weeks, 5 min/ session	VR therapy exercises	VAS pain score after 4 weeks of therapy
Schlechter [102]	2020	Parallel	Procedural	115 (58:57)	Peripheral venous catheterisation	11.3	47.8	Standard of Care	One session, 1-2 min	VR game	FPSR pain after procedure
Schmitt [103]	2011	Crossover	Procedural	54 (54:54)	Burn wound care physiotherapy	12.0	81.0	No distraction	One session on 1-5 days, 3-10 min / session	VR game SnowWorld	GRS worst pain during procedure, measured after intervention on day 5
Semerci [104]	2020	Parallel	Procedural	71 (35:36)	Port access	11.7	50.0	Standard of Care	One session, 5 min	3D video Rollercoaster	WBFPS pain score after procedure
Sharifpour [105]	2020	Parallel	Chronic	30 (15:15)	Cancer patients with chronic pain	14.9	Unknown	Standard of Care	One weekly session for 8 weeks, 30 min/session	3D video Ocean journey	Mcgill Pain questionnaire Pain at 7-day follow up after 8 weeks of therapy

Shetty [106]	2019	Parallel	Procedural	120 (60:60)	Dental treatment	5-8*	50.0	No distraction	One session, max. 45 min	Cartoon video	WBFPS pain score during procedure, measured after intervention
Smith [107]	2019	Parallel	Procedural	50 (25:25)	External cephalic version	31.6	0.0	Standard of Care	One session, 11 min	Interactive 3D 360 VR environment	VAS pain score during procedure, measured after intervention
Soltani [108]	2018	Crossover	Procedural	39 (39:39)	Burn wound care - physiotherapy	36.0	Unknown	Standard of Care	One session, 3 min	VR game SnowWorld	GRS worst pain during procedure, measured after intervention
Spiegel [109]	2019	Parallel	Acute	120 (61:59)	Pain in hospitalised patients	50.8	50.0	Television	Three sessions a day until discharge,10 min / session	VR meditation and VR games	NRS pain intensity 60-72 hours after first intervention
Sweta [110]	2019	Parallel	Procedural	50 (25:25)	Dental surgery, local injection	39.7	Unknown	Standard of Care	One session, unknown	VR video Relaxation	VAS pain score after surgery
Tanja-Dijkstra [111]	2018a	Parallel	Procedural	70 (20:28)	Dental treatment	45.1	42.9	No distraction	One session, 13 min	Passive 3D 360 VR environment UrbanVR	NRS pain score during procedure, measured after procedure
	2018b	Parallel	Procedural	70 (22:28)	Dental treatment	45.1	42.9	No distraction	One session, 13 min	Passive 3D 360 VR environment CoastVR	NRS pain score during procedure, measured after procedure
Tennant [113]	2020	Parallel	Acute	90 (61:29)	Cancer patients with acute pain	11.6	55.5	iPad	One session, 10 min	Passive 3D 360 VR environments	VAS pain score after intervention
Ustuner Top [114]	2021	Parallel	Procedural	77 (37:40)	Venipuncture	4.8	47.0	No distraction	One session, 6 min	3D video Aquarium	FPSR pain score during procedure, measured after procedure
van Twillert [115]	2007	Crossover	Procedural	19 (19:19)	Burn wound care	30.0	63.2	No distraction	One session, 19 min	VR game SnowWorld	VAT pain score during procedure, measured after intervention
Walker [116]	2014	Parallel	Procedural	43 (22:21)	Cystoscopy	42.6	100.0	Standard of Care	One session, unknown	VR game SnowWorld	VAS average pain during procedure, measured after intervention
Walther-Larsen [117]	2019	Parallel	Procedural	59 (28:31)	Peripheral venous catheterisation	10.5	88.1	Smartphone	One session, 10-20 min	VR game Seagull Splash	VAS pain score after procedure, measured 15 min after intervention
Wong [118]	2020	Parallel	Procedural	108 (54:54)	Peripheral venous catheterisation	10.4	51.9	Standard of Care	One session, 5 min	Animation video	VAS pain score after procedure

Wong [119]	2021	Parallel	Acute	40 (21:19)	Labor pain	32.0	100.0	Standard of Care	One session, 30 min	VR meditation	NRS pain score after intervention
Xiang [120]	2021a	Parallel	Procedural	90 (31:29)	Burn wound care	11.3	48.0	Distraction	One session, unknown	VR game Virtual river cruise	VAS average pain during procedure, measured after procedure
	2021b	Parallel	Procedural	90 (30:29)	Burn wound care	11.3	48.0	Distraction	One session, unknown	Passive 3D 360 VR environment	VAS average pain during procedure, measured after procedure
Yang [121]	2019	Parallel	Acute	48 (24:24)	Athroscopic knee surgery	35.3	37.5	Television	One session, unknown	3D video with Model of knee	VAS pain intensity on postoperative day 3
Yilmaz Yelvar [122]	2017	Parallel	Chronic	44 (22:22)	Low back pain	49.6	36.4	Conventional Rehabilitation	5 sessions a week for 2 weeks, 15 min/ session	3D video of a walking session	VAS pain after 2 weeks of therapy

EG: experimental group, CG: control group, WBFPS: Wong Baker FACES pain rating scale, VAS: Visual Analogue Scale, NRS: Numeric Rating Scale, FPS: Faces Pain Scale, FPSR: Faces Pain Scale-Revised, GRS: graphic rating scale, OFS: Oucher Face scale, APPT-WGRS: The Adolescent Pediatric Pain Tool (APPT) word graphic rating scale (WGRS), VAT: Visual Analogue Thermometer Score, ED: Emergency Department, OC: Outpatient clinic

References of included studies

[1] Akin B, Yilmaz Kocak M, Kucukaydin Z, Guzel K. The Effect of Showing Images of the Foetus with the Virtual Reality Glass During Labour Process on Labour Pain, Birth Perception and Anxiety. J Clin Nurs 2021;30(15-16):2301-2308.

[2] Al-Halabi MN, Bshara N, AlNerabieah Z. Effectiveness of audio visual distraction using virtual reality eyeglasses versus tablet device in child behavioral management during inferior alveolar nerve block. Anaesthesia, Pain and Intensive Care 2018;22(1):55-61.

[3] Ali RR, Selim AO, Abdel Ghafar MA, Abdelraouf OR, Ali OI. Virtual reality as a pain distractor during physical rehabilitation in pediatric burns. Burns 2021.

[4] Alshatrat SM, Alotaibi R, Sirois M, Malkawi Z. The use of immersive virtual reality for pain control during periodontal scaling and root planing procedures in dental hygiene clinic. Int J Dent Hyg 2019;17(1):71-76.

[5] Asl Aminabadi N, Erfanparast L, Sohrabi A, Ghertasi Oskouei S, Naghili A. The Impact of Virtual Reality Distraction on Pain and Anxiety during Dental Treatment in 4-6 Year-Old Children: a Randomized Controlled Clinical Trial. J Dent Res Dent Clin Dent Prospects 2012;6(4):117-124.

[6] Asvanund Y, Mitrakul K, Juhong RO, Arunakul M. Effect of audiovisual eyeglasses during local anesthesia injections in 5-to 8-year-old children. Quintessence Int 2015;46(6):513-521.

[7] Atzori B, Hoffman HG, Vagnoli L, Patterson DR, Alhalabi W, Messeri A, Lauro Grotto R. Virtual Reality Analgesia During Venipuncture in Pediatric Patients With Onco-Hematological Diseases. Front Psychol 2018;9:2508.

[8] Atzori B, Lauro Grotto R, Giugni A, Calabro M, Alhalabi W, Hoffman HG. Virtual Reality Analgesia for Pediatric Dental Patients. Front Psychol 2018;9:2265.

[9] Austin PD, Craig A, Middleton JW, Tran Y, Costa DSJ, Wrigley PJ, Siddall PJ. The short-term effects of head-mounted virtual-reality on neuropathic pain intensity in people with spinal cord injury pain: a randomised cross-over pilot study. Spinal Cord 2021;59(7):738-746.

[10] Aydın A, Özyazıcıoğlu N. Using a Virtual Reality Headset to Decrease Pain Felt During a Venipuncture Procedure in Children. J Perianesth Nurs 2019;34(6):1215-1221.

[11] Azurdia D, Alberto A, Watkins T, Lorimer I, Fernandez M, Jara M, Tolentino D, Furtado O, Jung TY. Effects Of Virtual Reality On Pain And Fatigue In Individuals With SCI. Medicine and Science in Sports and Exercise 2019;51(6):168-168.

[12] Babaie M, Shirinabadi Farahani A, Nourian M, Pourhoseingholi A, Masoumpoor A. The Effect of Audio-Visual Distraction on Catheterization Pain among School-Age Children. Evidence Based Care Journa 2019;9(1):35-42.

[13] Bani Mohammad E, Ahmad M. Virtual reality as a distraction technique for pain and anxiety among patients with breast cancer: A randomized control trial. Palliat Support Care 2019;17(1):29-34.

[14] Barad D, Tripathy P, Prusty BK, Pradhan R. A RANDOMIZED CONTROL TRIAL ONTHE EFFECT OF VIRTUAL REALITY VERSUS COLD VIBRATION ON PAIN AND PHYSIOLOGICAL PARAMETERS DURING PHLEBOTOMY AMONG CHILDREN. European Journal of Molecular & Clinical Medicine 2020;07(10):37888-33802.

[15] Basak T, Demirtas A, Yorubulut SM. Virtual reality and distraction cards to reduce pain during intramuscular benzathine penicillin injection procedure in adults: A randomized controlled trial. J Adv Nurs 2021;77(5):2511-2518.

[16] Basak T, Duman S, Demirtas A. Distraction-based relief of pain associated with peripheral intravenous catheterisation in adults: a randomised controlled trial. J Clin Nurs 2020;29(5-6):770-777.

[17] Bekelis K, Calnan D, Simmons N, MacKenzie TA, Kakoulides G. Effect of an Immersive Preoperative Virtual Reality Experience on Patient Reported Outcomes: A Randomized Controlled Trial. Ann Surg 2017;265(6):1068-1073.

[18] Bentsen B, Svensson P, Wenzel A. Evaluation of effect of 3D video glasses on perceived pain and unpleasantness induced by restorative dental treatment. Eur J Pain 2001;5(4):373-378.

[19] Bentsen B, Wenzel A, Svensson P. Comparison of the effect of video glasses and nitrous oxide analgesia on the perceived intensity of pain and unpleasantness evoked by dental scaling. Eur J Pain 2003;7(1):49-53.
[20] Brown L, Chen ET, Binder DS. The use of virtual reality for Peri-procedural pain and anxiety at an outpatient spine clinic injection visit: an exploratory controlled randomized trial. Am J Transl Res 2020;12(9):5818-5826.
[21] Buldur B, Candan M. Does virtual reality affect children's dental anxiety, pain, and behaviour? A randomised, placebo-controlled, cross-over trial. Pesquisa Brasileira em Odontopediatria e Clinica Integrada 2020;21:1-14.
[22] Butt M, Kabariti S, Likourezos A, Drapkin J, Luthra D, Hossain R, Brazg J, Motov S. TAKE-PAUSE: Efficacy of Mindfulness-Based Virtual Reality as an Intervention in the Pediatric Emergency Department. Acad Emerg Med 2021.
[23] Canares T, Parrish C, Santos C, Badawi A, Kleinman K, Stewart A, Biddle A, Brylske P, McGuire J. Optimizing coping during venipuncture procedures with virtualreality in the pediatric emergency department: A randomizedcontrolled trial. Pediatrics 2021;147(3):307-308.
[24] Carrougher GJ, Hoffman HG, Nakamura D, Lezotte D, Soltani M, Leahy L, Engrav LH, Patterson DR. The effect of virtual reality on pain and range of motion in adults with burn injuries. J Burn Care Res 2009;30(5):785-791.
[25] Caruso TJ, George A, Menendez M, De Souza E, Khoury M, Kist MN, Rodriguez ST. Virtual reality during pediatric vascular access: A pragmatic, prospective randomized, controlled trial. Paediatr Anaesth 2020;30(2):116-123.
[26] Chan E, Hovenden M, Ramage E, Ling N, Pham JH, Rahim A, Lam C, Liu L, Foster S, Sambell R, Jeyachanthiran K, Crock C, Stock A, Hopper SM, Cohen S, Davidson A, Plummer K, Mills E, Craig SS, Deng G, Leong P. Virtual Reality for Pediatric Needle Procedural Pain: Two Randomized Clinical Trials. J Pediatr 2019;209:160-167 e164.
[27] Chan EA, Chung JW, Wong TK, Lien AS, Yang JY. Application of a virtual reality prototype for pain relief of pediatric burn in Taiwan. J Clin Nurs 2007;16(4):786-793.
[28] Chang J, Ninan S, Liu K, Iloreta AM, Kirke D, Courey M. Enhancing Patient Experience in Office-Based Laryngology Procedures With Passive Virtual Reality. OTO Open 2021;5(1):2473974x20975020.
[29] Chen YJ, Cheng SF, Lee PC, Lai CH, Hou IC, Chen CW. Distraction using virtual reality for children during intravenous injections in an emergency department: A randomised trial. J Clin Nurs 2020;29(3-4):503-510.
[30] Clerc PGB, Arneja JS, Zwimpfer CM. Virtual Reality vs. Standard-of-Care for Comfort During Minor Plastic Surgery Procedures in Children. Plast Reconstr Surg 2021;148:400-408.
[31] Darnall BD, Krishnamurthy P, Tsuei J, Minor JD. Self-Administered Skills-Based Virtual Reality Intervention for Chronic Pain: Randomized Controlled Pilot Study. JMIR Form Res 2020;4(7):e17293.
[32] Das DA, Grimmer KA, Sparnon AL, McRae SE, Thomas BH. The efficacy of playing a virtual reality game in modulating pain for children with acute burn injuries: a randomized controlled trial [ISRCTN87413556]. BMC Pediatr 2005;5(1):1.
[33] De Silva AP, Niriella MA, Nandamuni Y, Nanayakkara SD, Perera KR, Kodisinghe SK, Subasinghe KC, Pathmeswaran A, de Silva HJ. Effect of audio and visual distraction on patients undergoing colonoscopy: a randomized controlled study. Endosc Int Open 2016;4(11):E1211-E1214.
[34] Deo N, Khan KS, Mak J, Allotey J, Gonzalez Carreras FJ, Fusari G, Benn J. Virtual reality for acute pain in outpatient hysteroscopy: a randomised controlled trial. BJOG 2020.
[35] Ding J, He Y, Chen L, Zhu B, Cai Q, Chen K, Liu G. Virtual reality distraction decreases pain during daily dressing changes following haemorrhoid surgery. J Int Med Res 2019;47(9):4380-4388.
[36] Dumoulin S, Bouchard S, Ellis J, Lavoie KL, Vezina MP, Charbonneau P, Tardif J, Hajjar A. A Randomized Controlled Trial on the Use of Virtual Reality for Needle-Related Procedures in Children and Adolescents in the Emergency Department. Games Health J 2019;8(4):285-293.

[37] Ebrahimi H, Namdar H, Ghahramanpour M, Ghafourifard M, Musavi S. Effect of virtual reality method and multimedia system on burn patients' pain during dressing. Journal of Clinical and Analytical Medicine 2017;8(suppl 5):485-489.
[38] Eijlers R, Dierckx B, Staals LM, Berghmans JM, van der Schroeff MP, Strabbing EM, Wijnen RMH, Hillegers MHJ, Legerstee JS, Utens E. Virtual reality exposure before elective day care surgery to reduce anxiety and pain in children: A randomised controlled trial. Eur J Anaesthesiol 2019;36(10):728-737.
[39] El-Sharkawi HF, El-Housseiny AA, Aly AM. Effectiveness of new distraction technique on pain associated with injection of local anesthesia for children. Pediatr Dent 2012;34(2):e35-38.
[40] Erdogan B, Aytekin Ozdemir A. The Effect of Three Different Methods on Venipuncture Pain and Anxiety in Children: Distraction Cards, Virtual Reality, and Buzzy(R) (Randomized Controlled Trial). J Pediatr Nurs 2021;58:e54-e62.
[41] Felemban OM, Alshamrani RM, Aljeddawi DH, Bagher SM. Effect of virtual reality distraction on pain and anxiety during infiltration anesthesia in pediatric patients: a randomized clinical trial. BMC Oral Health 2021;21(1):321.
[42] Fouks Y, Kern G, Cohen A, Reicher L, Shapira Z, Many A, Yogev Y, Rattan G. A virtual reality system for pain and anxiety management during outpatient hysteroscopy-A randomized control trial. Eur J Pain 2021.
[43] Frey DP, Bauer ME, Bell CL, Low LK, Hassett AL, Cassidy RB, Boyer KD, Sharar SR. Virtual Reality Analgesia in Labor: The VRAIL Pilot Study-A Preliminary Randomized Controlled Trial Suggesting Benefit of Immersive Virtual Reality Analgesia in Unmedicated Laboring Women. Anesth Analg 2019;128(6):e93-e96.
[44] Furman E, Jasinevicius TR, Bissada NF, Victoroff KZ, Skillicorn R, Buchner M. Virtual reality distraction for pain control during periodontal scaling and root planing procedures. J Am Dent Assoc 2009;140(12):1508-1516.
[45] Gerçeker G, Bektaş M, Aydınok Y, Ören H, Ellidokuz H, Olgun N. The effect of virtual reality on pain, fear, and anxiety during access of a port with huber needle in pediatric hematology-oncology patients: Randomized controlled trial. Eur J Oncol Nurs 2021;50:101886.
[46] Gershon J, Zimand E, Pickering M, Rothbaum BO, Hodges L. A pilot and feasibility study of virtual reality as a distraction for children with cancer. J Am Acad Child Adolesc Psychiatry 2004;43(10):1243-1249.
[47] Gold JI, Kim SH, Kant AJ, Joseph MH, Rizzo AS. Effectiveness of virtual reality for pediatric pain distraction during i.v. placement. Cyberpsychol Behav 2006;9(2):207-212.
[48] Gold JI, Mahrer NE. Is Virtual Reality Ready for Prime Time in the Medical Space? A Randomized Control Trial of Pediatric Virtual Reality for Acute Procedural Pain Management. J Pediatr Psychol 2018;43(3):266-275.
[49] Gold JI, SooHoo M, Laikin AM, Lane AS, Klein MJ. Effect of an Immersive Virtual Reality Intervention on Pain and Anxiety Associated With Peripheral Intravenous Catheter Placement in the Pediatric Setting: A Randomized Clinical Trial. JAMA Netw Open 2021;4(8):e2122569.
[50] Goldman RD, Behboudi A. Pilot Randomized Controlled Trial of Virtual Reality vs. Standard-of-Care During Pediatric Laceration Repair. J Child Adolesc Trauma 2021;14(2):295-298.
[51] Goldman RD, Behboudi A. Virtual reality for intravenous placement in the emergency department-a randomized controlled trial. Eur J Pediatr 2021;180(3):725-731.
[52] Gray ML, Goldrich DY, McKee S, Schaberg M, Del Signore A, Govindaraj S, Iloreta AM. Virtual Reality as Distraction Analgesia for Office-Based Procedures: A Randomized Crossover-Controlled Trial. Otolaryngol Head Neck Surg 2021;164(3):580-588.
[53] Groninger H, Stewart D, Fisher JM, Tefera E, Cowgill J, Mete M. Virtual reality for pain management in advanced heart failure: A randomized controlled study. Palliat Med 2021;35(10):2008-2016.

[54] Gulsen C Pt M, Soke F Pt P, Eldemir K Pt M, Apaydin Y Pt M, Ozkul C Pt P, Guclu-Gunduz A Pt P, Akcali Dt Md P. Effect of fully immersive virtual reality treatment combined with exercise in fibromyalgia patients: a randomized controlled trial. Assist Technol 2020:1-8.
[55] Guo C, Deng H, Yang J. Effect of virtual reality distraction on pain among patients with hand injury undergoing dressing change. J Clin Nurs 2015;24(1-2):115-120.
[56] Gur EY, Apay SE. The effect of cognitive behavioral techniques using virtual reality on birth pain: a randomized controlled trial. Midwifery 2020;91:102856.
[57] Haisley KR, Straw OJ, Muller DT, Antiporda MA, Zihni AM, Reavis KM, Bradley DD, Dunst CM. Feasibility of implementing a virtual reality program as an adjuvant tool for peri-operative pain control; Results of a randomized controlled trial in minimally invasive foregut surgery. Complement Ther Med 2020;49:102356.
[58] Hassannia R, Sajjadi M, Shareinia H, Elmimehr R, Moravejjifar M. Effect of Virtual Reality on Relieving Pain and Anxiety of Circumcision in Children. Evidence Based Care Journal 2021;10:67-74.
[59] Hoffman HG, Patterson DR, Carrougher GJ. Use of virtual reality for adjunctive treatment of adult burn pain during physical therapy: a controlled study. Clin J Pain 2000;16(3):244-250.
[60] Hoffman HG, Patterson DR, Carrougher GJ, Sharar SR. Effectiveness of virtual reality-based pain control with multiple treatments. Clin J Pain 2001;17(3):229-235.
[61] Hoffman HG, Patterson DR, Seibel E, Soltani M, Jewett-Leahy L, Sharar SR. Virtual reality pain control during burn wound debridement in the hydrotank. Clin J Pain 2008;24(4):299-304.
[62] Hoxhallari E, Behr IJ, Bradshaw JS, Morkos MS, Haan PS, Schaefer MC, Clarkson JHW. Virtual Reality Improves the Patient Experience during Wide-Awake Local Anesthesia No Tourniquet Hand Surgery: A Single-Blind, Randomized, Prospective Study. Plast Reconstr Surg 2019;144(2):408-414.
[63] Hua Y, Qiu R, Yao WY, Zhang Q, Chen XL. The Effect of Virtual Reality Distraction on Pain Relief During Dressing Changes in Children with Chronic Wounds on Lower Limbs. Pain Manag Nurs 2015;16(5):685-691.
[64] Hwang H, Cho S, Lee JH. The effect of virtual body swapping with mental rehearsal on pain intensity and body perception disturbance in complex regional pain syndrome. Int J Rehabil Res 2014;37(2):167-172.
[65] Inangil D, Sendir M, Buyukyilmaz F. Efficacy of Cartoon Viewing Devices During Phlebotomy in Children: A Randomized Controlled Trial. J Perianesth Nurs 2020;35(4):407-412.
[66] JahaniShoorab N, Ebrahimzadeh Zagami S, Nahvi A, Mazluom SR, Golmakani N, Talebi M, Pabarja F. The Effect of Virtual Reality on Pain in Primiparity Women during Episiotomy Repair: A Randomize Clinical Trial. Iran J Med Sci 2015;40(3):219-224.
[67] Jeffs D, Dorman D, Brown S, Files A, Graves T, Kirk E, Meredith-Neve S, Sanders J, White B, Swearingen CJ. Effect of virtual reality on adolescent pain during burn wound care. J Burn Care Res 2014;35(5):395-408.
[68] Jin C, Feng Y, Ni Y, Shan Z. Virtual reality intervention in postoperative rehabilitation after total knee arthroplasty: a prospective and randomized controlled clinical trial. International Journal of Clinical and Experimental Medicine 2018;11(6):6119-6124.
[69] Jin W, Choo A, Gromala D, Shaw C, Squire P. A Virtual Reality Game for Chronic Pain Management: A Randomized, Controlled Clinical Study. Stud Health Technol Inform 2016;220:154-160.
[70] Joo Y, Kim EK, Song HG, Jung H, Park H, Moon JY. Effectiveness of virtual reality immersion on procedure-related pain and anxiety in outpatient pain clinic: an exploratory randomized controlled trial. Korean J Pain 2021;34(3):304-314.
[71] Karaman D, Taşdemir N. The Effect of Using Virtual Reality During Breast Biopsy on Pain and Anxiety: A Randomized Controlled Trial. J Perianesth Nurs 2021.
[72] Karaveli Çakır S, Evirgen S. The Effect of Virtual Reality on Pain and Anxiety During Colonoscopy: A Randomized Controlled Trial. Turk J Gastroenterol 2021;32(5):451-457.

[73] Kipping B, Rodger S, Miller K, Kimble RM. Virtual reality for acute pain reduction in adolescents undergoing burn wound care: a prospective randomized controlled trial. Burns 2012;38(5):650-657.
[74] Koc Ozkan T, Polat F. The Effect of Virtual Reality and Kaleidoscope on Pain and Anxiety Levels During Venipuncture in Children. J Perianesth Nurs 2020;35(2):206-211.
[75] Konstantatos AH, Angliss M, Costello V, Cleland H, Stafrace S. Predicting the effectiveness of virtual reality relaxation on pain and anxiety when added to PCA morphine in patients having burns dressings changes. Burns 2009;35(4):491-499.
[76] Koushali AN, Daryabeigi R, Alimohammadi N, Najaf M. The Effect of a Multi-Dimensional Play Program on Children's Pain Intensity During Burn Dressing Change in Burn Intensive Care Units: A clinical Trial. Journal of Critical Care Nursing 2017;10(4e58845).
[77] Laghlam D, Naudin C, Coroyer L, Aidan V, Malvy J, Rahoual G, Estagnasié P, Squara P. Virtual reality vs. Kalinox® for management of pain in intensive care unit after cardiac surgery: a randomized study. Ann Intensive Care 2021;11(1):74.
[78] Le May S, Paquin D, Fortin JS, Khadra C, Acm. DREAM Project: Using virtual reality to decrease pain and anxiety of children with burns during treatments, Proceedings of the Virtual Reality International Conference (VRIC). NEW YORK: Assoc Computing Machinery, 2016.
[79] Litwin SP, Nguyen C, Hundert A, Stuart S, Liu D, Maguire B, Matava C, Stinson J. Virtual Reality to Reduce Procedural Pain During IV Insertion in the Pediatric Emergency Department: A Pilot Randomized Controlled Trial. Clin J Pain 2021;37(2):94-101.
[80] Liu KY, Ninan SJ, Laitman BM, Goldrich DY, Iloreta AM, Londino AV, 3rd. Virtual Reality as Distraction Analgesia and Anxiolysis for Pediatric Otolaryngology Procedures. Laryngoscope 2021;131(5):E1714-E1721.
[81] Łuczak M, Nowak Ł, Chorbińska J, Galik K, Kiełb P, Łaszkiewicz J, Tukiendorf A, Kościelska-Kasprzak K, Małkiewicz B, Zdrojowy R, Szydełko T, Krajewski W. Influence of Virtual Reality Devices on Pain and Anxiety in Patients Undergoing Cystoscopy Performed under Local Anaesthesia. J Pers Med 2021;11(11).
[82] Maani CV, Hoffman HG, Morrow M, Maiers A, Gaylord K, McGhee LL, DeSocio PA. Virtual reality pain control during burn wound debridement of combat-related burn injuries using robot-like arm mounted VR goggles. J Trauma 2011;71(1 Suppl):S125-130.
[83] McSherry T, Atterbury M, Gartner S, Helmold E, Searles DM, Schulman C. Randomized, Crossover Study of Immersive Virtual Reality to Decrease Opioid Use During Painful Wound Care Procedures in Adults. J Burn Care Res 2018;39(2):278-285.
[84] Melcer Y, Maymon R, Gal-Kochav M, Pekar-Zlotin M, Levinsohn-Tavor O, Meizner I, Svirsky R. Analgesic efficacy of virtual reality for acute pain in amniocentesis: A randomized controlled trial. Eur J Obstet Gynecol Reprod Biol 2021;261:134-138.
[85] Mitrakul K, Asvanund Y, Arunakul M, Paka-Akekaphat S. Effect of audiovisual eyeglasses during dental treatment in 5-8 year-old children. Eur J Paediatr Dent 2015;16(3):239-245.
[86] Mohanasundari SK, Raghu VA, Joseph J, Mohan R, Sharma S. Effectiveness of Flippits and Virtual Reality Therapy on Pain and Anxiety Among Children Undergoing Painful Procedures. Cureus 2021;13(8):e17134.
[87] Momenyan N, Safaei AA, Hantoushzadeh S. Immersive virtual reality analgesia in un-medicated laboring women (during stage 1 and 2): A randomized controlled trial. Clinical and Experimental Obstetrics and Gynecology 2021;48(1):110-116.
[88] Morris LD, Louw QA, Crous LC. Feasibility and potential effect of a low-cost virtual reality system on reducing pain and anxiety in adult burn injury patients during physiotherapy in a developing country. Burns 2010;36(5):659-664.
[89] Mukherjee M, Bedekar N, Sancheti PK, Shyam A. Immediate and ShortTerm Effect of Virtual Reality Training on Pain, Range of Motion, and Kinesiophobia in Patients with Cervical Spondylosis. Indian J Phys Ther Res 2020;2:55-60.

[90] Niharika P, Reddy NV, Srujana P, Srikanth K, Daneswari V, Geetha KS. Effects of distraction using virtual reality technology on pain perception and anxiety levels in children during pulp therapy of primary molars. J Indian Soc Pedod Prev Dent 2018;36(4):364-369.
[91] Nunna M, Dasaraju RK, Kamatham R, Mallineni SK, Nuvvula S. Comparative evaluation of virtual reality distraction and counter-stimulation on dental anxiety and pain perception in children. J Dent Anesth Pain Med 2019;19(5):277-288.
[92] Nusser M, Knapp S, Kramer M, Krischak G. Effects of virtual reality-based neck-specific sensorimotor training in patients with chronic neck pain: A randomized controlled pilot trial. J Rehabil Med 2021;53(2):jrm00151.
[93] Osmanlliu E, Trottier ED, Bailey B, Lagace M, Certain M, Khadra C, Sanchez M, Theriault C, Paquin D, Cotes-Turpin C, Le May S. Distraction in the Emergency department using Virtual reality for INtravenous procedures in Children to Improve comfort (DEVINCI): a pilot pragmatic randomized controlled trial. CJEM 2021;23(1):94-102.
[94] Ozalp Gerceker G, Ayar D, Ozdemir EZ, Bektas M. Effects of virtual reality on pain, fear and anxiety during blood draw in children aged 5-12 years old: A randomised controlled study. J Clin Nurs 2020;29(7-8):1151-1161.
[95] Ozalp Gerceker G, Binay S, Bilsin E, Kahraman A, Yilmaz HB. Effects of Virtual Reality and External Cold and Vibration on Pain in 7- to 12-Year-Old Children During Phlebotomy: A Randomized Controlled Trial. J Perianesth Nurs 2018;33(6):981-989.
[96] Powers MB, Carl E, Levihn-Coon A, Van Veldhuizen M, Caven A, Pogue J, Fresnedo M, Turner ED, Adams M, Leonard K, Conroy H, Lantrip C, Caven T, Isbell C, Regner J, Garmon E, Foreman M, Miller W, Fares LA, Carlbring P, Otto MW, Weiss DN, Hughes J, Bernhardt JM, Roy R, Oh J, Copt R, MacClements J, Warren AM, Rosenfield B, Rosenfield D, Minns S, Telch MJ, Smits JAJ. Nonpharmacologic Pain Management Among Hospitalized Inpatients: A Randomized Waitlist-Controlled Trial of Standard Virtual Reality (CGI VR) Versus Video Capture VR (360 degrees 3D/Stereoscopic Video Capture VR). Clin J Pain 2021;37(9):678-687.
[97] Ran L, Zhao N, Fan L, Zhou P, Zhang C, Yu C. Application of virtual reality on non-drug behavioral management of short-term dental procedure in children. Trials 2021;22(1):562.
[98] Rousseaux F, Dardenne N, Massion PB, Ledoux D, Bicego A, Donneau AF, Faymonville ME, Nyssen AS, Vanhaudenhuyse A. Virtual reality and hypnosis for anxiety and pain management in intensive care units: A prospective randomised trial among cardiac surgery patients. Eur J Anaesthesiol 2021.
[99] Sander Wint S, Eshelman D, Steele J, Guzzetta CE. Effects of distraction using virtual reality glasses during lumbar punctures in adolescents with cancer. Oncol Nurs Forum 2002;29(1):E8-E15.
[100] Sarig Bahat H, Croft K, Carter C, Hoddinott A, Sprecher E, Treleaven J. Remote kinematic training for patients with chronic neck pain: a randomised controlled trial. Eur Spine J 2018;27(6):1309-1323.
[101] Sarig Bahat H, Takasaki H, Chen X, Bet-Or Y, Treleaven J. Cervical kinematic training with and without interactive VR training for chronic neck pain - a randomized clinical trial. Man Ther 2015;20(1):68-78.
[102] Schlechter AK, Whitaker W, Iyer S, Gabriele G, Wilkinson M. Virtual reality distraction during pediatric intravenous line placement in the emergency department: A prospective randomized comparison study. Am J Emerg Med 2021;44:296-299.
[103] Schmitt YS, Hoffman HG, Blough DK, Patterson DR, Jensen MP, Soltani M, Carrougher GJ, Nakamura D, Sharar SR. A randomized, controlled trial of immersive virtual reality analgesia, during physical therapy for pediatric burns. Burns 2011;37(1):61-68.
[104] Semerci R, Akgün Kostak M, Eren T, Avci G. Effects of Virtual Reality on Pain During Venous Port Access in Pediatric Oncology Patients: A Randomized Controlled Study. J Pediatr Oncol Nurs 2021;38(2):142-151.

[105] Sharifpour S, Manshaee G, Sajjadian I. Effects of virtual reality therapy on perceived pain intensity, anxiety, catastrophising and self-efficacy among adolescents with cancer. Couns Psychother Res 2020(00):1-9.
[106] Shetty V, Suresh LR, Hegde AM. Effect of Virtual Reality Distraction on Pain and Anxiety During Dental Treatment in 5 to 8 Year Old Children. J Clin Pediatr Dent 2019;43(2):97-102.
[107] Smith V, Warty RR, Kashyap R, Neil P, Adriaans C, Nair A, Krishnan S, Da Silva Costa F, Vollenhoven B, Wallace EM. A randomised controlled trial to assess the feasibility of utilising virtual reality to facilitate analgesia during external cephalic version. Sci Rep 2020;10(1):3141.
[108] Soltani M, Drever SA, Hoffman HG, Sharar SR, Wiechman SA, Jensen MP, Patterson DR. Virtual reality analgesia for burn joint flexibility: A randomized controlled trial. Rehabil Psychol 2018;63(4):487-494.
[109] Spiegel B, Fuller G, Lopez M, Dupuy T, Noah B, Howard A, Albert M, Tashjian V, Lam R, Ahn J, Dailey F, Rosen BT, Vrahas M, Little M, Garlich J, Dzubur E, IsHak W, Danovitch I. Virtual reality for management of pain in hospitalized patients: A randomized comparative effectiveness trial. PLoS One 2019;14(8):e0219115.
[110] Sweta VR, Abhinav RP, Ramesh A. Role of Virtual Reality in Pain Perception of Patients Following the Administration of Local Anesthesia. Ann Maxillofac Surg 2019;9(1):110-113.
[111] Tanja-Dijkstra K, Pahl S, White MP, Auvray M, Stone RJ, Andrade J, May J, Mills I, Moles DR. The Soothing Sea: A Virtual Coastal Walk Can Reduce Experienced and Recollected Pain. Environ Behav 2018;50(6):599-625.
[112] Tejera DM, Beltran-Alacreu H, Cano-de-la-Cuerda R, Leon Hernandez JV, Martin-Pintado-Zugasti A, Calvo-Lobo C, Gil-Martinez A, Fernandez-Carnero J. Effects of Virtual Reality versus Exercise on Pain, Functional, Somatosensory and Psychosocial Outcomes in Patients with Non-specific Chronic Neck Pain: A Randomized Clinical Trial. Int J Environ Res Public Health 2020;17(16).
[113] Tennant M, Youssef GJ, McGillivray J, Clark TJ, McMillan L, McCarthy MC. Exploring the use of Immersive Virtual Reality to enhance Psychological Well-Being in Pediatric Oncology: A pilot randomized controlled trial. Eur J Oncol Nurs 2020;48:101804.
[114] Ustuner Top F, Kuzlu Ayyıldız T. Pain management in children during invasive procedures: A randomized clinical trial. Nurs Forum 2021;56(4):816-822.
[115] van Twillert B, Bremer M, Faber AW. Computer-generated virtual reality to control pain and anxiety in pediatric and adult burn patients during wound dressing changes. J Burn Care Res 2007;28(5):694-702.
[116] Walker MR, Kallingal GJ, Musser JE, Folen R, Stetz MC, Clark JY. Treatment efficacy of virtual reality distraction in the reduction of pain and anxiety during cystoscopy. Mil Med 2014;179(8):891-896.
[117] Walther-Larsen S, Petersen T, Friis SM, Aagaard G, Drivenes B, Opstrup P. Immersive Virtual Reality for Pediatric Procedural Pain: A Randomized Clinical Trial. Hosp Pediatr 2019;9(7):501-507.
[118] Wong CL, Li CK, Chan CWH, Choi KC, Chen J, Yeung MT, Chan ON. Virtual Reality Intervention Targeting Pain and Anxiety Among Pediatric Cancer Patients Undergoing Peripheral Intravenous Cannulation: A Randomized Controlled Trial. Cancer Nurs 2021;44(6):435-442.
[119] Wong MS, Spiegel BMR, Gregory KD. Virtual Reality Reduces Pain in Laboring Women: A Randomized Controlled Trial. Am J Perinatol 2021;38(S 01):e167-e172.
[120] Xiang H, Shen J, Wheeler KK, Patterson J, Lever K, Armstrong M, Shi J, Thakkar RK, Groner JI, Noffsinger D, Giles SA, Fabia RB. Efficacy of Smartphone Active and Passive Virtual Reality Distraction vs Standard Care on Burn Pain Among Pediatric Patients: A Randomized Clinical Trial. JAMA Netw Open 2021;4(6):e2112082.
[121] Yang JH, Ryu JJ, Nam E, Lee HS, Lee JK. Effects of Preoperative Virtual Reality Magnetic Resonance Imaging on Preoperative Anxiety in Patients Undergoing Arthroscopic Knee Surgery: A Randomized Controlled Study. Arthroscopy 2019;35(8):2394-2399.

[122] Yilmaz Yelvar GD, Cirak Y, Dalkilinc M, Parlak Demir Y, Guner Z, Boydak A. Is physiotherapy integrated virtual walking effective on pain, function, and kinesiophobia in patients with non-specific low-back pain? Randomised controlled trial. Eur Spine J 2017;26(2):538-545.

Appendix 3: Risk of Bias
Appendix 3a: Analysis of individual studies

Parallel studies	D1	D2	D3	D4	D5	Overall
Akin2021	−	+	+	+	+	−
AlHalabi2018	+	+	+	?	?	!
Ali2021	+	+	+	?	+	!
Aydin2019	−	+	+	?	?	−
Babaie2019	+	+	+	?	+	!
BaniMohammad2018	−	+	+	?	?	−
Barad2020	+	+	−	?	+	−
Basak2020	+	+	+	+	−	−
Basak2021	?	+	+	+	+	!
Bekelis2017	+	+	+	+	+	+
Brown2020	?	+	+	?	+	!
Butt 2021	?	+	+	+	+	!
Canares2021	?	+	+	?	+	!
Caruso2020	+	+	−	?	+	−
Chan2019	+	+	+	+	?	!
Chang2021	?	+	+	?	?	!
Chen2020	+	+	+	?	+	!
Clerc2021	+	+	+	?	+	!
Darnall2020	?	+	−	?	?	−
Deo2020	+	+	+	?	+	!
DeSilva2016	?	+	+	+	+	!
Ding2019	?	+	+	?	+	!
Dumoulin2019	+	+	+	?	?	!
Ebrahimi2017	?	+	+	?	+	!
Eijlers2019	+	+	+	+	+	+
Erdogan2021	+	+	?	?	+	!
Felemban2021	+	+	+	+	+	+
Fouks2021	+	+	+	?	+	!
Gershon2004	?	+	+	?	?	!
Gold2006	?	+	+	?	?	!
Gold2018	+	+	+	?	?	!
Gold2021	+	+	?	?	+	!
Goldman2021a	?	+	−	?	+	−
Goldman2021b	+	+	+	?	+	!
Groninger2021	+	+	+	+	+	+
Gulsen2020	?	+	−	+	?	−
Guo2014	?	+	+	+	?	!
Gür2020	+	+	+	+	?	!
Haisley2020	+	+	+	?	?	!
Hassania2021	?	+	+	?	?	!
Hoxhallari2019	+	−	−	?	?	−
Hua2015	?	+	+	?	?	!
Hwang2014	?	+	+	?	?	!
Inangil2020	+	+	+	?	+	!
JahaniShoorab2015	?	+	+	?	+	!
Jeffs2014	+	+	+	+	?	!
Jin2018	?	+	+	?	?	!
Joo2021	+	+	+	+	+	+
Karaman2021	?	+	+	?	?	!
KaraveliÇakır2021	+	+	+	?	?	!
Kipping2012	+	+	+	?	?	!
KocÖzkan2019	+	+	?	?	+	!
Konstantatos2009	?	+	+	?	?	!
Koushali2017	?	+	+	?	+	!
Laghlam2021	?	+	?	?	+	!
Litwin2021	?	+	+	?	+	!
Liu2020	?	+	+	?	?	!
Luczak2021	?	+	+	?	?	!

+	Low risk
?	Some concerns
−	High risk

D1	Randomisation process
D2	Deviations from the intended interventions
D3	Missing outcome data
D4	Measurement of the outcome
D5	Selection of the reported result

Parallel studies	D1	D2	D3	D4	D5	Overall
Melcer2021	?	+	+	?	+	!
Mohanasundari2021	+	+	+	+	+	+
Momenyan2021	?	+	?	+	+	+
MoralesTejera2020	+	+	+	+	+	+
Mukherjee2020	+	+	+	+	+	+
Nunna2019	?	+	+	?	?	!
Nusser2021	?	+	+	?	?	!
Osmanlliu2020	+	+	+	?	+	!
OzalpGerceker2018	+	+	+	?	?	!
OzalpGerceker2020	+	+	+	+	+	+
OzalpGerceker2021	?	+	+	+	+	!
Powers2021	+	+	+	?	?	!
Ran2021	+	+	?	?	+	!
Rousseaux2021	?	+	?	?	+	!
SanderWint2002	?	+	+	?	?	!
SarigBahat2015	+	+	+	+	+	+
SarigBahat2017	+	+	?	+	+	!
Schlechter2020	?	+	+	?	+	!
Semerci2020	+	+	+	?	?	!
Sharifpour2020	+	+	+	?	?	!
Shetty2019	?	+	+	?	?	!
Smith2019	+	+	?	?	+	!
Spiegel2019	?	+	−	?	+	−
Sweta2019	?	+	?	?	?	!
Tanja-Dijkstra2018	+	+	+	?	+	!
Tennant2020	+	+	+	?	?	!
UstunerTop2021	?	+	+	?	?	!
Walker2014	+	+	+	?	?	!
Walther-Larsen2019	+	+	+	+	?	!
Wong2020	+	+	+	?	+	!
Wong2021	+	+	+	?	+	!
Xiang2021	+	+	+	?	+	!
Yang2019	+	+	+	+	+	+
YilmazYelvar2017	?	+	+	+	?	!

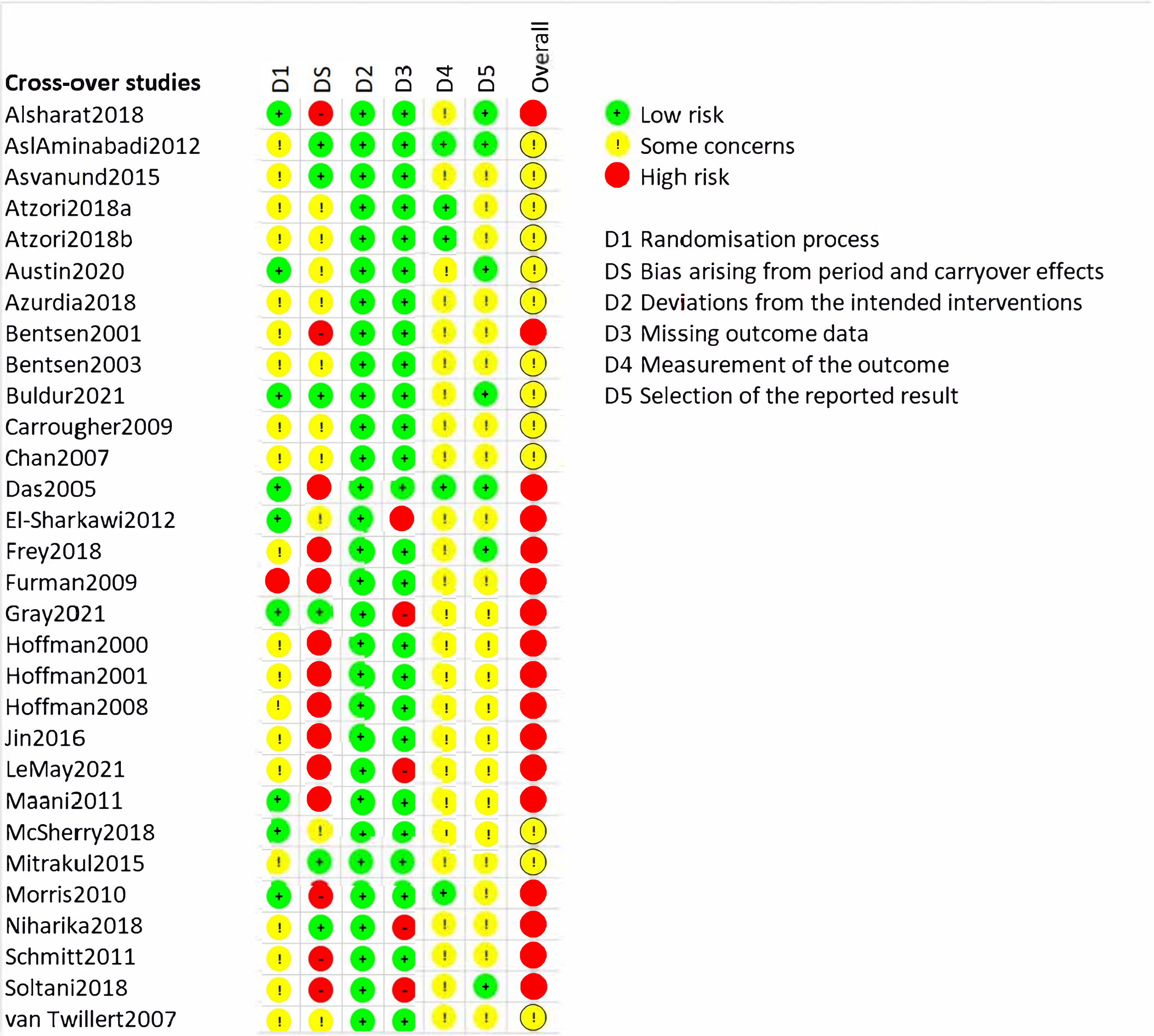
Cross-over studies
D1
DS
D2
D3
D4
D5
Overall
Alsharat2018
AslAminabadi2012
Asvanund2015
Atzori2018a
Atzori2018b
Austin2020
Azurdia2018
Bentsen2001
Bentsen2003
Buldur2021
Carrougher2009
Chan2007
Das2005
El-Sharkawi2012
Frey2018
Furman2009
Gray2021
Hoffman2000
Hoffman2001
Hoffman2008
Jin2016
LeMay2021
Maani2011
McSherry2018
Mitrakul2015
Morris2010
Niharika2018
Schmitt2011
Soltani2018
van Twillert2007
Low risk
Some concerns
High risk
D1 Randomisation process
DS Bias arising from period and carryover effects
D2 Deviations from the intended interventions
D3 Missing outcome data
D4 Measurement of the outcome
D5 Selection of the reported result

Appendix 3b: Risk of bias in parallel studies - Summary

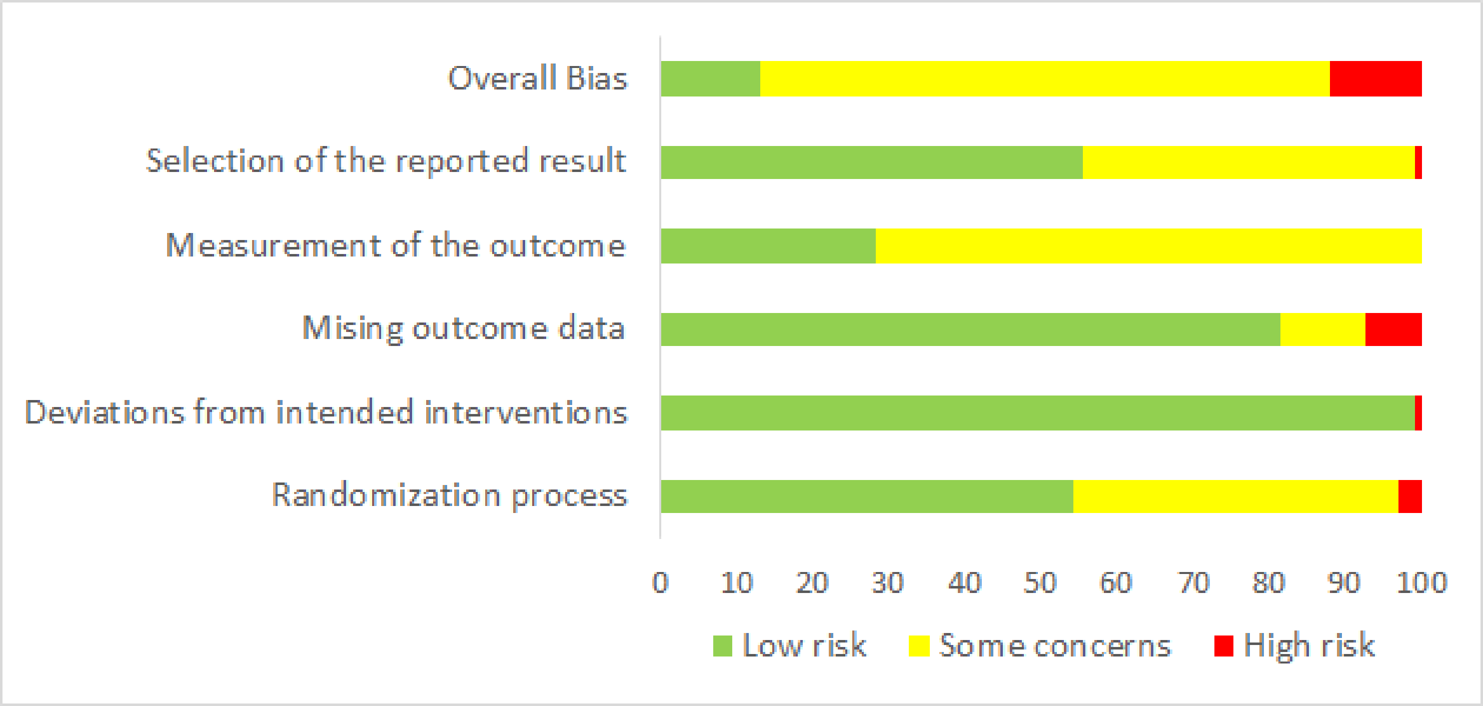

Appendix 3c: Risk of bias in cross-over studies - Summary

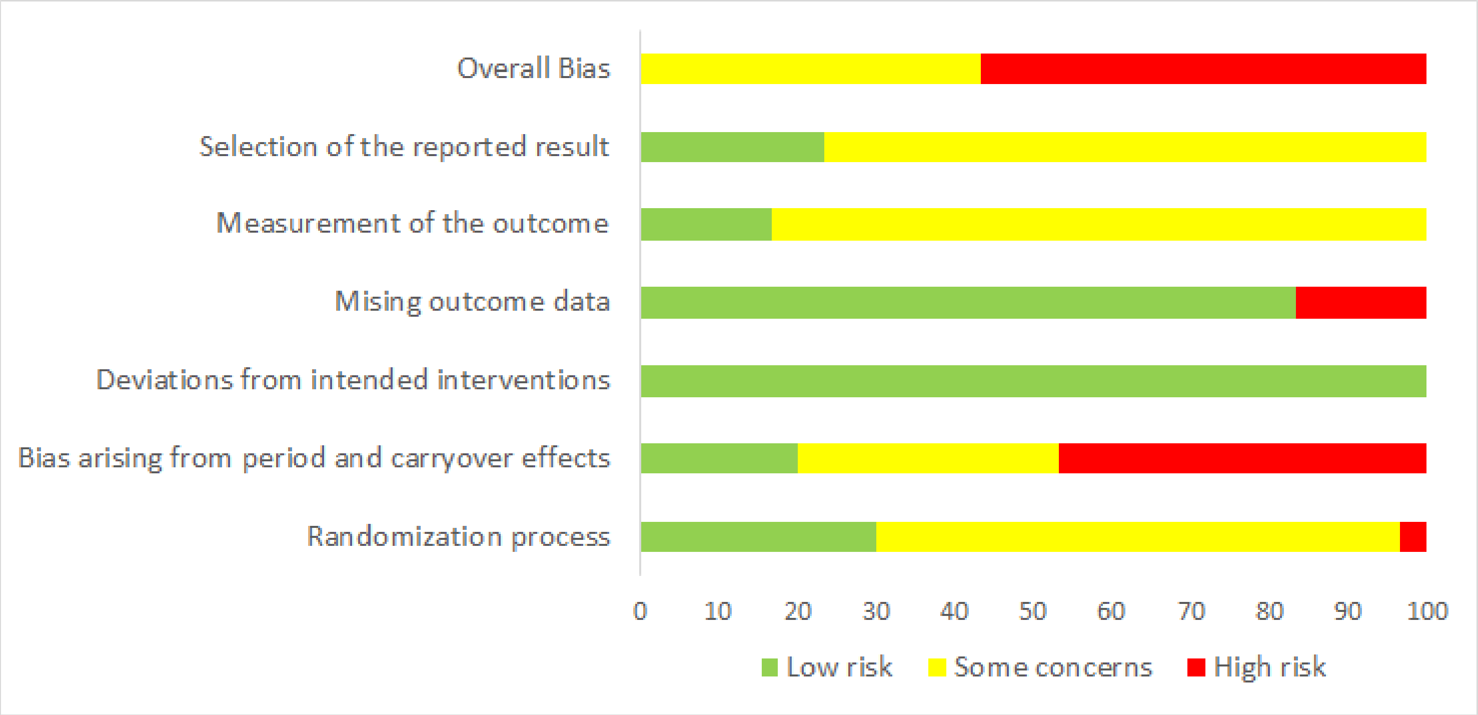

Appendix 3d: Funnelplot

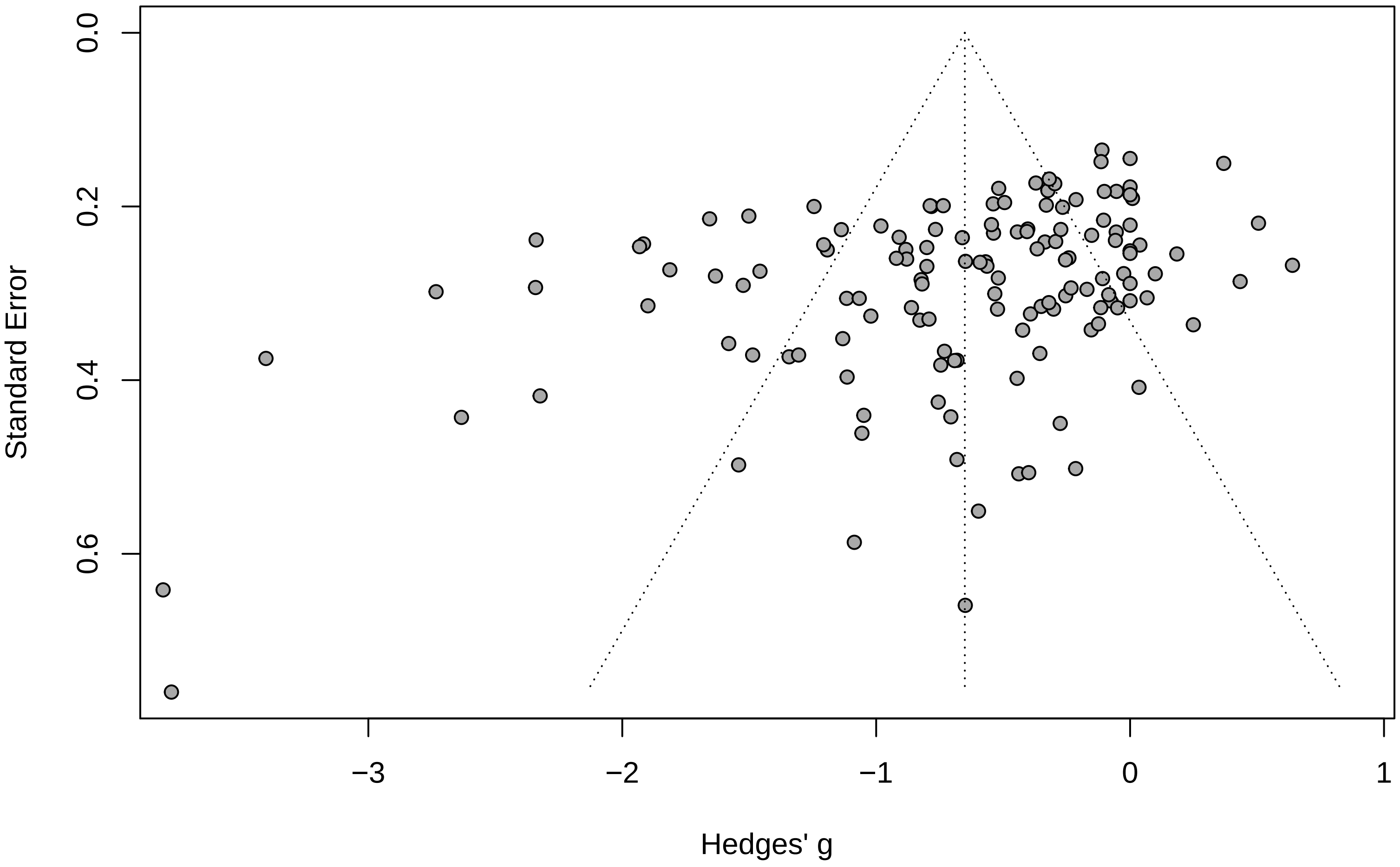

Appendix 4: Primary analysis
Meta-analysis on the effect of VR on self-reported pain scores compared to the control group (SMD)

Study	Experimental Total	Experimental Mean	Experimental SD	Control Total	Control Mean	Control SD	SMD	95%−CI	Weight
Akin 2021	50	4.68	1.44	50	6.74	0.45	−1.92	[−2.39; −1.44]	0.8%
Al−Halabi 2018	33	0.75	0.98	34	0.71	0.86	0.04	[−0.44; 0.52]	0.8%
Ali 2021	11	3.10	1.04	11	7.72	1.30	−3.78	[−5.26; −2.29]	0.3%
Alshatrat 2018	50	10.00	3.88	50	11.05	3.96	−0.27	[−0.66; 0.13]	0.8%
Asl Aminabadi 2012a	58	1.89	0.65	58	3.00	0.81	−1.50	[−1.92; −1.09]	0.8%
Asl Aminabadi 2012b	59	2.05	0.60	59	3.05	0.60	−1.66	[−2.08; −1.24]	0.8%
Asvanund 2015a	23	1.57	2.29	23	3.04	3.08	−0.53	[−1.12; 0.06]	0.7%
Asvanund 2015b	21	2.23	2.29	21	2.46	3.46	−0.08	[−0.68; 0.53]	0.7%
Atzori 2018a	15	2.00	1.20	15	3.60	3.00	−0.68	[−1.42; 0.06]	0.7%
Atzori 2018b	5	2.20	1.79	5	3.80	2.59	−0.65	[−1.94; 0.64]	0.4%
Austin 2020	16	2.20	1.60	16	3.40	1.60	−0.73	[−1.45; −0.01]	0.7%
Aydin 2019	60	3.07	2.86	60	3.23	3.05	−0.05	[−0.41; 0.30]	0.8%
Azurdia 2018	11	1.82	1.60	11	4.10	1.22	−1.54	[−2.52; −0.57]	0.5%
Babaie 2019	32	26.40	7.35	30	36.80	6.02	−1.52	[−2.09; −0.95]	0.7%
Bani Mohammad 2018	40	0.33	0.82	40	4.84	2.57	−2.34	[−2.92; −1.77]	0.7%
Barad 2020	36	2.50	1.75	36	8.00	1.43	−3.40	[−4.14; −2.67]	0.7%
Basak 2020	40	3.50	2.84	40	4.72	3.15	−0.40	[−0.85; 0.04]	0.8%
Basak 2021	30	4.93	3.07	31	6.67	2.17	−0.65	[−1.16; −0.13]	0.8%
Bekelis 2017	64	30.00	11.11	63	30.00	7.41	0.00	[−0.35; 0.35]	0.8%
Bentsen 2001	23	31.57	24.69	23	35.74	23.55	−0.17	[−0.75; 0.41]	0.7%
Bentsen 2003	26	16.90	18.40	26	15.20	15.20	0.10	[−0.44; 0.64]	0.8%
Brown 2020	15	2.60	1.90	15	4.30	2.80	−0.69	[−1.43; 0.05]	0.7%
Buldur 2021a	38	4.00	1.85	38	4.11	2.13	−0.05	[−0.50; 0.40]	0.8%
Buldur 2021b	36	3.52	1.65	34	4.11	1.83	−0.34	[−0.81; 0.14]	0.8%
Butt 2021	55	3.58	3.10	55	3.55	3.30	0.01	[−0.36; 0.38]	0.8%
Canares 2021	19	4.13	3.88	20	5.70	3.96	−0.39	[−1.03; 0.24]	0.7%
Carrougher 2009	39	40.00	30.00	39	55.00	25.00	−0.54	[−0.99; −0.09]	0.8%
Caruso 2020	106	0.72	1.32	114	0.89	1.68	−0.11	[−0.38; 0.15]	0.9%
Chan 2007	8	38.13	34.00	8	53.75	33.38	−0.44	[−1.43; 0.56]	0.5%
Chan 2019a	64	3.08	3.29	59	4.20	3.59	−0.32	[−0.68; 0.03]	0.8%
Chan 2019b	63	2.41	3.11	66	4.21	3.76	−0.52	[−0.87; −0.17]	0.8%
Chang 2021	8	2.64	2.21	8	3.53	2.00	−0.40	[−1.39; 0.59]	0.5%
Chen 2020	68	3.35	2.38	68	4.35	2.95	−0.37	[−0.71; −0.03]	0.8%
Clerc 2021	35	0.00	2.70	29	0.00	5.40	0.00	[−0.49; 0.49]	0.8%
Darnall 2020	35	3.20	3.88	39	3.80	3.96	−0.15	[−0.61; 0.31]	0.8%
Das2005	7	1.30	1.80	7	4.10	2.90	−1.09	[−2.24; 0.06]	0.5%
De Silva 2016	67	4.00	2.96	67	5.00	3.70	−0.30	[−0.64; 0.04]	0.8%
Deo 2020	20	3.77	2.66	20	6.00	2.62	−0.83	[−1.48; −0.18]	0.7%
Ding 2019	91	5.60	14.31	91	7.30	15.26	−0.11	[−0.41; 0.18]	0.9%
Dumoulin 2019	20	21.75	20.92	15	25.33	25.25	−0.15	[−0.82; 0.52]	0.7%
Ebrahimi 2017	20	5.35	2.68	20	5.60	1.35	−0.12	[−0.74; 0.50]	0.7%
Eijlers 2019	94	2.00	2.96	97	2.00	1.85	0.00	[−0.28; 0.28]	0.9%
El−Sharkawi 2012	42	1.48	1.52	42	2.76	1.78	−0.77	[−1.21; −0.32]	0.8%
Erdogan 2021	37	2.70	2.80	34	5.20	2.80	−0.88	[−1.37; −0.39]	0.8%
Felemban 2021	25	2.40	2.82	25	2.72	2.99	−0.11	[−0.66; 0.45]	0.7%
Fouks 2021	44	5.00	6.75	38	5.00	5.67	0.00	[−0.43; 0.43]	0.8%
Frey 2018	27	5.60	2.00	27	7.15	1.70	−0.82	[−1.38; −0.27]	0.7%
Furman 2009a	38	1.58	1.40	38	3.66	2.00	−1.19	[−1.68; −0.70]	0.8%
Furman 2009b	38	2.39	1.80	38	3.66	2.00	−0.66	[−1.12; −0.20]	0.8%
Gershon 2004	22	13.80	20.20	22	15.40	17.00	−0.08	[−0.68; 0.51]	0.7%
Gold 2006	10	1.80	2.31	10	2.40	1.84	−0.28	[−1.16; 0.61]	0.6%
Gold 2018	70	1.31	1.59	73	1.93	2.22	−0.32	[−0.65; 0.01]	0.8%
Gold 2021	53	1.09	1.82	54	2.19	2.21	−0.54	[−0.93; −0.15]	0.8%
Goldman 2021a	35	2.00	5.40	31	4.00	5.40	−0.37	[−0.85; 0.12]	0.8%
Goldman 2021b	32	1.84	1.94	30	1.47	2.03	0.18	[−0.32; 0.68]	0.8%
Gray 2021a	43	3.11	2.35	43	3.37	2.58	−0.10	[−0.53; 0.32]	0.8%
Gray 2021b	39	2.53	2.37	39	3.62	2.49	−0.44	[−0.89; 0.01]	0.8%
Groninger 2021	52	3.80	2.40	36	5.10	2.30	−0.55	[−0.98; −0.11]	0.8%
Gulsen 2020	8	3.95	1.59	8	4.50	3.04	−0.21	[−1.20; 0.77]	0.5%
Guo 2014	49	2.63	1.27	49	7.64	3.41	−1.93	[−2.41; −1.45]	0.8%
Gür 2020a	54	4.98	1.69	54	6.38	1.86	−0.78	[−1.17; −0.39]	0.8%
Gür 2020b	55	4.96	1.72	54	6.38	1.86	−0.79	[−1.18; −0.40]	0.8%
Gür 2020c	55	5.96	2.05	54	6.38	1.86	−0.21	[−0.59; 0.16]	0.8%
Haisley 2020	26	3.80	3.88	26	3.90	3.96	−0.03	[−0.57; 0.52]	0.8%
Hassannia 2021	20	1.10	0.30	20	2.30	0.65	−2.32	[−3.14; −1.50]	0.6%
Hoffman 2000	12	14.67	15.24	12	36.33	23.73	−1.05	[−1.91; −0.19]	0.6%
Hoffman 2001	7	5.00	38.83	7	30.00	39.57	−0.60	[−1.68; 0.48]	0.5%
Hoffman 2008	11	5.10	2.60	11	7.60	1.90	−1.06	[−1.96; −0.15]	0.6%
Hoxhallari 2019	21	0.14	0.47	20	0.60	1.14	−0.52	[−1.15; 0.10]	0.7%
Hua 2015	33	2.42	1.85	32	4.19	2.12	−0.88	[−1.39; −0.37]	0.8%
Hwang 2014	13	5.08	2.02	13	6.15	2.60	−0.45	[−1.22; 0.33]	0.6%
Inangil 2020	40	1.30	2.15	40	4.95	3.65	−1.21	[−1.69; −0.73]	0.8%
JahaniShoorab 2015	15	16.70	16.50	15	39.30	22.50	−1.11	[−1.89; −0.34]	0.6%
Jeffs 2014	8	28.70	15.70	10	38.40	11.60	−0.68	[−1.65; 0.28]	0.5%
Jin 2016	33	36.90	18.60	33	53.10	16.10	−0.92	[−1.43; −0.41]	0.8%
Jin 2018	20	3.87	0.55	20	4.42	0.79	−0.79	[−1.44; −0.15]	0.7%
Joo 2021	19	3.70	1.40	19	5.50	1.70	−1.13	[−1.82; −0.44]	0.7%
Karaman 2021	30	2.86	1.22	30	6.33	2.24	−1.90	[−2.52; −1.28]	0.7%
Karaveli Çakir 2021	30	2.76	1.25	30	3.76	2.11	−0.57	[−1.09; −0.05]	0.8%
Kipping 2012	20	3.90	3.88	21	5.30	3.96	−0.35	[−0.97; 0.27]	0.7%
Koc Özkan 2019	46	1.97	1.20	43	6.81	2.20	−2.73	[−3.32; −2.15]	0.7%
Konstantatos 2009	43	7.30	3.88	43	5.30	3.96	0.51	[0.08; 0.94]	0.8%
Koushali 2017	20	5.50	1.30	20	8.40	0.80	−2.63	[−3.50; −1.77]	0.6%
Laghlam 2021	90	5.00	5.40	90	3.00	5.40	0.37	[0.07; 0.66]	0.9%
Le May 2021	20	3.80	3.40	20	4.80	3.10	−0.30	[−0.92; 0.32]	0.7%
Litwin 2021	31	2.60	2.10	27	3.80	2.10	−0.56	[−1.09; −0.04]	0.8%
Liu 2020	30	0.80	1.06	23	2.26	2.38	−0.82	[−1.39; −0.25]	0.7%
Luczak 2021	52	4.30	2.20	51	5.10	2.60	−0.33	[−0.72; 0.06]	0.8%
Maani 2011	12	4.50	1.95	12	6.25	2.49	−0.76	[−1.59; 0.08]	0.6%
McSherry 2018	12	5.80	2.90	12	5.70	2.60	0.04	[−0.77; 0.84]	0.6%
Melcer 2021	30	2.50	1.50	30	3.80	1.70	−0.80	[−1.33; −0.27]	0.8%
Mitrakul 2015a	21	0.86	1.49	21	1.62	2.94	−0.32	[−0.93; 0.29]	0.7%
Mitrakul 2015b	21	1.90	2.93	21	1.90	3.32	0.00	[−0.60; 0.60]	0.7%
Mohanasundari 2021	35	3.00	1.35	35	5.00	9.45	−0.29	[−0.76; 0.18]	0.8%
Momenyan 2021	26	9.01	0.78	26	9.37	0.57	−0.52	[−1.07; 0.03]	0.7%
Morales Tejera 2020	22	2.67	1.91	22	3.11	1.47	−0.25	[−0.85; 0.34]	0.7%
Morris 2010	11	3.00	4.44	11	6.00	3.70	−0.71	[−1.57; 0.16]	0.6%
Mukherjee 2020	22	1.50	0.80	21	2.71	1.45	−1.02	[−1.66; −0.38]	0.7%
Niharika 2018a	18	2.56	1.65	18	5.22	2.18	−1.34	[−2.07; −0.61]	0.7%
Niharika 2018b	18	2.33	1.57	18	5.44	2.90	−1.31	[−2.03; −0.58]	0.7%
Nunna 2019	35	2.94	1.76	35	3.06	2.31	−0.06	[−0.53; 0.41]	0.8%
Nusser 2021	17	2.20	1.20	18	3.20	3.00	−0.42	[−1.09; 0.25]	0.7%
Osmanlliu 2020	31	3.00	6.75	31	3.00	6.08	0.00	[−0.50; 0.50]	0.8%
Ozalp Gerceker 2018	40	1.50	3.88	40	5.10	3.96	−0.91	[−1.37; −0.45]	0.8%
Ozalp Gerceker 2020a	45	1.20	2.20	46	4.10	3.50	−0.98	[−1.42; −0.55]	0.8%
Ozalp Gerceker 2020b	45	1.00	1.50	46	4.10	3.50	−1.14	[−1.58; −0.69]	0.8%
Ozalp Gerceker 2021	21	2.40	1.80	21	5.30	1.80	−1.58	[−2.28; −0.88]	0.7%
Powers 2021a	34	3.03	2.30	34	6.37	2.23	−1.46	[−2.00; −0.92]	0.8%
Powers 2021b	35	2.77	2.13	34	6.37	2.23	−1.63	[−2.18; −1.08]	0.7%
Ran 2021	60	1.58	1.08	60	2.86	0.96	−1.24	[−1.64; −0.85]	0.8%
Rousseaux 2021a	15	5.50	3.88	22	4.50	3.96	0.25	[−0.41; 0.91]	0.7%
Rousseaux 2021b	15	4.00	3.88	22	4.50	3.96	−0.12	[−0.78; 0.53]	0.7%
Sander Wint 2002	17	3.00	3.88	13	6.00	3.96	−0.75	[−1.50; 0.00]	0.6%
Sarig Bahat 2015	30	22.10	24.10	30	27.72	21.90	−0.24	[−0.75; 0.27]	0.8%
Sarig Bahat 2017	16	31.10	23.60	14	39.45	22.00	−0.36	[−1.08; 0.37]	0.7%
Schlechter 2020	58	4.00	2.22	57	4.00	4.44	0.00	[−0.37; 0.37]	0.8%
Schmitt 2011	54	40.00	30.86	54	55.00	29.39	−0.49	[−0.88; −0.11]	0.8%
Semerci 2020	35	2.34	3.27	36	5.02	3.35	−0.80	[−1.29; −0.32]	0.8%
Sharifpour 2020	15	40.47	4.45	15	62.80	6.73	−3.81	[−5.07; −2.55]	0.4%
Shetty 2019	60	2.42	1.47	60	5.60	1.22	−2.34	[−2.81; −1.87]	0.8%
Smith 2019	25	60.68	21.10	25	49.76	28.00	0.43	[−0.13; 0.99]	0.7%
Soltani 2018	39	52.90	27.80	39	64.40	28.30	−0.41	[−0.85; 0.04]	0.8%
Spiegel 2019	61	4.70	3.88	59	5.10	3.96	−0.10	[−0.46; 0.26]	0.8%
Sweta 2019	25	1.28	0.89	25	2.60	1.38	−1.12	[−1.72; −0.52]	0.7%
Tanja−Dijkstra 2018a	20	2.05	2.63	28	2.63	2.32	−0.23	[−0.81; 0.34]	0.7%
Tanja−Dijkstra 2018b	22	0.63	0.93	28	2.63	2.32	−1.07	[−1.67; −0.47]	0.7%
Tennant 2020	61	10.05	16.43	29	14.76	18.48	−0.27	[−0.72; 0.17]	0.8%
Ustuner Top 2021	37	3.82	1.20	40	6.96	2.08	−1.81	[−2.35; −1.28]	0.8%
van Twillert 2007	19	3.00	1.70	19	5.60	1.73	−1.49	[−2.21; −0.76]	0.7%
Walker 2014	22	44.00	16.90	21	43.00	11.90	0.07	[−0.53; 0.67]	0.7%
Walther−Larsen 2019	28	27.00	18.52	31	15.00	18.52	0.64	[0.11; 1.16]	0.8%
Wong 2020	54	1.94	1.73	54	4.00	3.53	−0.74	[−1.13; −0.35]	0.8%
Wong 2021	21	5.50	2.40	19	5.60	1.40	−0.05	[−0.67; 0.57]	0.7%
Xiang 2021a	31	24.90	34.62	29	47.10	39.57	−0.59	[−1.11; −0.07]	0.8%
Xiang 2021b	30	37.00	38.83	29	47.10	39.57	−0.25	[−0.77; 0.26]	0.8%
Yang 2019	24	30.00	27.05	24	30.00	27.04	0.00	[−0.57; 0.57]	0.7%
Yilmaz Yelvar 2017	22	2.52	1.80	22	4.90	3.39	−0.86	[−1.48; −0.24]	0.7%
**Random effects model**	**4596**			**4542**			**−0.65**	**[−0.76; −0.54]**	**100.0%**

Heterogeneity: $I^2 = 84\%$, $\tau^2 = 0.3497$, $p < 0.01$

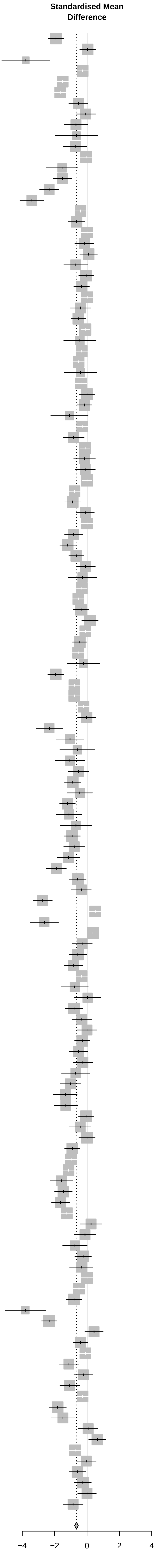

Appendix 5: Sensitivity analyses

Appendix 5A: Sensitivity analysis excluding studies of which the control group was included twice

Study	Experimental Total	Experimental Mean	Experimental SD	Control Total	Control Mean	Control SD	SMD	95%−CI	Weight
Akin 2021	50	4.68	1.44	50	6.74	0.45	−1.92	[−2.39; −1.44]	0.9%
Al−Halabi 2018	33	0.75	0.98	34	0.71	0.86	0.04	[−0.44; 0.52]	0.9%
Ali 2021	11	3.10	1.04	11	7.72	1.30	−3.78	[−5.26; −2.29]	0.4%
Alshatrat 2018	50	10.00	3.88	50	11.05	3.96	−0.27	[−0.66; 0.13]	0.9%
Asl Aminabadi 2012a	58	1.89	0.65	58	3.00	0.81	−1.50	[−1.92; −1.09]	0.9%
Asl Aminabadi 2012b	59	2.05	0.60	59	3.05	0.60	−1.66	[−2.08; −1.24]	0.9%
Asvanund 2015a	23	1.57	2.29	23	3.04	3.08	−0.53	[−1.12; 0.06]	0.8%
Asvanund 2015b	21	2.23	2.29	21	2.46	3.46	−0.08	[−0.68; 0.53]	0.8%
Atzori 2018a	15	2.00	1.20	15	3.60	3.00	−0.68	[−1.42; 0.06]	0.7%
Atzori 2018b	5	2.20	1.79	5	3.80	2.59	−0.65	[−1.94; 0.64]	0.5%
Austin 2020	16	2.20	1.60	16	3.40	1.60	−0.73	[−1.45; −0.01]	0.8%
Aydin 2019	60	3.07	2.86	60	3.23	3.05	−0.05	[−0.41; 0.30]	0.9%
Azurdia 2018	11	1.82	1.60	11	4.10	1.22	−1.54	[−2.52; −0.57]	0.6%
Babaie 2019	32	26.40	7.35	30	36.80	6.02	−1.52	[−2.09; −0.95]	0.8%
Bani Mohammad 2018	40	0.33	0.82	40	4.84	2.57	−2.34	[−2.92; −1.77]	0.8%
Barad 2020	36	2.50	1.75	36	8.00	1.43	−3.40	[−4.14; −2.67]	0.7%
Basak 2020	40	3.50	2.84	40	4.72	3.15	−0.40	[−0.85; 0.04]	0.9%
Basak 2021	30	4.93	3.07	31	6.67	2.17	−0.65	[−1.16; −0.13]	0.9%
Bekelis 2017	64	30.00	11.11	63	30.00	7.41	0.00	[−0.35; 0.35]	0.9%
Bentsen 2001	23	31.57	24.69	23	35.74	23.55	−0.17	[−0.75; 0.41]	0.8%
Bentsen 2003	26	16.90	18.40	26	15.20	15.20	0.10	[−0.44; 0.64]	0.8%
Brown 2020	15	2.60	1.90	15	4.30	2.80	−0.69	[−1.43; 0.05]	0.7%
Buldur 2021a	38	4.00	1.85	38	4.11	2.13	−0.05	[−0.50; 0.40]	0.9%
Buldur 2021b	36	3.52	1.65	34	4.11	1.83	−0.34	[−0.81; 0.14]	0.9%
Butt 2021	55	3.58	3.10	55	3.55	3.30	0.01	[−0.36; 0.38]	0.9%
Canares 2021	19	4.13	3.88	20	5.70	3.96	−0.39	[−1.03; 0.24]	0.8%
Carrougher 2009	39	40.00	30.00	39	55.00	25.00	−0.54	[−0.99; −0.09]	0.9%
Caruso 2020	106	0.72	1.32	114	0.89	1.68	−0.11	[−0.38; 0.15]	1.0%
Chan 2007	8	38.13	34.00	8	53.75	33.38	−0.44	[−1.43; 0.56]	0.6%
Chan 2019a	64	3.08	3.29	59	4.20	3.59	−0.32	[−0.68; 0.03]	0.9%
Chan 2019b	63	2.41	3.11	66	4.21	3.76	−0.52	[−0.87; −0.17]	0.9%
Chang 2021	8	2.64	2.21	8	3.53	2.00	−0.40	[−1.39; 0.59]	0.6%
Chen 2020	68	3.35	2.38	68	4.35	2.95	−0.37	[−0.71; −0.03]	0.9%
Clerc 2021	35	0.00	2.70	29	0.00	5.40	0.00	[−0.49; 0.49]	0.9%
Darnall 2020	35	3.20	3.88	39	3.80	3.96	−0.15	[−0.61; 0.31]	0.9%
Das2005	7	1.30	1.80	7	4.10	2.90	−1.09	[−2.24; 0.06]	0.5%
De Silva 2016	67	4.00	2.96	67	5.00	3.70	−0.30	[−0.64; 0.04]	0.9%
Deo 2020	20	3.77	2.66	20	6.00	2.62	−0.83	[−1.48; −0.18]	0.8%
Ding 2019	91	5.60	14.31	91	7.30	15.26	−0.11	[−0.41; 0.18]	1.0%
Dumoulin 2019	20	21.75	20.92	15	25.33	25.25	−0.15	[−0.82; 0.52]	0.8%
Ebrahimi 2017	20	5.35	2.68	20	5.60	1.35	−0.12	[−0.74; 0.50]	0.8%
Eijlers 2019	94	2.00	2.96	97	2.00	1.85	0.00	[−0.28; 0.28]	1.0%
El−Sharkawi 2012	42	1.48	1.52	42	2.76	1.78	−0.77	[−1.21; −0.32]	0.9%
Erdogan 2021	37	2.70	2.80	34	5.20	2.80	−0.88	[−1.37; −0.39]	0.9%
Feleman 2021	25	2.40	2.82	25	2.72	2.99	−0.11	[−0.66; 0.45]	0.8%
Fouks 2021	44	5.00	6.75	38	5.00	5.67	0.00	[−0.43; 0.43]	0.9%
Frey 2018	27	5.60	2.00	27	7.15	1.70	−0.82	[−1.38; −0.27]	0.8%
Gershon 2004	22	13.80	20.20	22	15.40	17.00	−0.08	[−0.68; 0.51]	0.8%
Gold 2006	10	1.80	2.31	10	2.40	1.84	−0.28	[−1.16; 0.61]	0.7%
Gold 2018	70	1.31	1.59	73	1.93	2.22	−0.32	[−0.65; 0.01]	1.0%
Gold 2021	53	1.09	1.82	54	2.19	2.21	−0.54	[−0.93; −0.15]	0.9%
Goldman 2021a	35	2.00	5.40	31	4.00	5.40	−0.37	[−0.85; 0.12]	0.9%
Goldman 2021b	32	1.84	1.94	30	1.47	2.03	0.18	[−0.32; 0.68]	0.9%
Gray 2021a	43	3.11	2.35	43	3.37	2.58	−0.10	[−0.53; 0.32]	0.9%
Gray 2021b	39	2.53	2.37	39	3.62	2.49	−0.44	[−0.89; 0.01]	0.9%
Groninger 2021	52	3.80	2.40	36	5.10	2.30	−0.55	[−0.98; −0.11]	0.9%
Gulsen 2020	8	3.95	1.59	8	4.50	3.04	−0.21	[−1.20; 0.77]	0.6%
Guo 2014	49	2.63	1.27	49	7.64	3.41	−1.93	[−2.41; −1.45]	0.9%
Haisley 2020	26	3.80	3.88	26	3.90	3.96	−0.03	[−0.57; 0.52]	0.8%
Hassannia 2021	20	1.10	0.30	20	2.30	0.65	−2.32	[−3.14; −1.50]	0.7%
Hoffman 2000	12	14.67	15.24	12	36.33	23.73	−1.05	[−1.91; −0.19]	0.7%
Hoffman 2001	7	5.00	38.83	7	30.00	39.57	−0.60	[−1.68; 0.48]	0.6%
Hoffman 2008	11	5.10	2.60	11	7.60	1.90	−1.06	[−1.96; −0.15]	0.7%
Hoxhallari 2019	21	0.14	0.47	20	0.60	1.14	−0.52	[−1.15; 0.10]	0.8%
Hua 2015	33	2.42	1.85	32	4.19	2.12	−0.88	[−1.39; −0.37]	0.9%
Hwang 2014	13	5.08	2.02	13	6.15	2.60	−0.45	[−1.22; 0.33]	0.7%
Inangil 2020	40	1.30	2.15	40	4.95	3.65	−1.21	[−1.69; −0.73]	0.9%
JahaniShoorab 2015	15	16.70	16.50	15	39.30	22.50	−1.11	[−1.89; −0.34]	0.7%
Jeffs 2014	8	28.70	15.70	10	38.40	11.60	−0.68	[−1.65; 0.28]	0.6%
Jin 2016	33	36.90	18.60	33	53.10	16.10	−0.92	[−1.43; −0.41]	0.9%
Jin 2018	20	3.87	0.55	20	4.42	0.79	−0.79	[−1.44; −0.15]	0.8%
Joo 2021	19	3.70	1.40	19	5.50	1.70	−1.13	[−1.82; −0.44]	0.8%
Karaman 2021	30	2.86	1.22	30	6.33	2.24	−1.90	[−2.52; −1.28]	0.8%
Karaveli Çakir 2021	30	2.76	1.25	30	3.76	2.11	−0.57	[−1.09; −0.05]	0.9%
Kipping 2012	20	3.90	3.88	21	5.30	3.96	−0.35	[−0.97; 0.27]	0.8%
Koc Özkan 2019	46	1.97	1.20	43	6.81	2.20	−2.73	[−3.32; −2.15]	0.8%
Konstantatos 2009	43	7.30	3.88	43	5.30	3.96	0.51	[0.08; 0.94]	0.9%
Koushali 2017	20	5.50	1.30	20	8.40	0.80	−2.63	[−3.50; −1.77]	0.7%
Laghlam 2021	90	5.00	5.40	90	3.00	5.40	0.37	[0.07; 0.66]	1.0%
Le May 2021	20	3.80	3.40	20	4.80	3.10	−0.30	[−0.92; 0.32]	0.8%
Litwin 2021	31	2.60	2.10	27	3.80	2.10	−0.56	[−1.09; −0.04]	0.9%
Liu 2020	30	0.80	1.06	23	2.26	2.38	−0.82	[−1.39; −0.25]	0.8%
Luczak 2021	52	4.30	2.20	51	5.10	2.60	−0.33	[−0.72; 0.06]	0.9%
Maani 2011	12	4.50	1.95	12	6.25	2.49	−0.76	[−1.59; 0.08]	0.7%
McSherry 2018	12	5.80	2.90	12	5.70	2.60	0.04	[−0.77; 0.84]	0.7%
Melcer 2021	30	2.50	1.50	30	3.80	1.70	−0.80	[−1.33; −0.27]	0.9%
Mitrakul 2015a	21	0.86	1.49	21	1.62	2.94	−0.32	[−0.93; 0.29]	0.8%
Mitrakul 2015b	21	1.90	2.93	21	1.90	3.32	0.00	[−0.60; 0.60]	0.8%
Mohanasundari 2021	35	3.00	1.35	35	5.00	9.45	−0.29	[−0.76; 0.18]	0.9%
Momenyan 2021	26	9.01	0.78	26	9.37	0.57	−0.52	[−1.07; 0.03]	0.8%
Morales Tejera 2020	22	2.67	1.91	22	3.11	1.47	−0.25	[−0.85; 0.34]	0.8%
Morris 2010	11	3.00	4.44	11	6.00	3.70	−0.71	[−1.57; 0.16]	0.7%
Mukherjee 2020	22	1.50	0.80	21	2.71	1.45	−1.02	[−1.66; −0.38]	0.8%
Niharika 2018a	18	2.56	1.65	18	5.22	2.18	−1.34	[−2.07; −0.61]	0.7%
Niharika 2018b	18	2.33	1.57	18	5.44	2.90	−1.31	[−2.03; −0.58]	0.7%
Nunna 2019	35	2.94	1.76	35	3.06	2.31	−0.06	[−0.53; 0.41]	0.9%
Nusser 2021	17	2.20	1.20	18	3.20	3.00	−0.42	[−1.09; 0.25]	0.8%
Osmanlliu 2020	31	3.00	6.75	31	3.00	6.08	0.00	[−0.50; 0.50]	0.9%
Ozalp Gerceker 2018	40	1.50	3.88	40	5.10	3.96	−0.91	[−1.37; −0.45]	0.9%
Ozalp Gerceker 2021	21	2.40	1.80	21	5.30	1.80	−1.58	[−2.28; −0.88]	0.8%
Ran 2021	60	1.58	1.08	60	2.86	0.96	−1.24	[−1.64; −0.85]	0.9%
Sander Wint 2002	17	3.00	3.88	13	6.00	3.96	−0.75	[−1.50; 0.00]	0.7%
Sarig Bahat 2015	30	22.10	24.10	30	27.72	21.90	−0.24	[−0.75; 0.27]	0.9%
Sarig Bahat 2017	16	31.10	23.60	14	39.45	22.00	−0.36	[−1.08; 0.37]	0.7%
Schlechter 2020	58	4.00	2.22	57	4.00	4.44	0.00	[−0.37; 0.37]	0.9%
Schmitt 2011	54	40.00	30.86	54	55.00	29.39	−0.49	[−0.88; −0.11]	0.9%
Semerci 2020	35	2.34	3.27	36	5.02	3.35	−0.80	[−1.29; −0.32]	0.9%
Sharifpour 2020	15	40.47	4.45	15	62.80	6.73	−3.81	[−5.07; −2.55]	0.5%
Shetty 2019	60	2.42	1.47	60	5.60	1.22	−2.34	[−2.81; −1.87]	0.9%
Smith 2019	25	60.68	21.10	25	49.76	28.00	0.43	[−0.13; 0.99]	0.8%
Soltani 2018	39	52.90	27.80	39	64.40	28.30	−0.41	[−0.85; 0.04]	0.9%
Spiegel 2019	61	4.70	3.88	59	5.10	3.96	−0.10	[−0.46; 0.26]	0.9%
Sweta 2019	25	1.28	0.89	25	2.60	1.38	−1.12	[−1.72; −0.52]	0.8%
Tennant 2020	61	10.05	16.43	29	14.76	18.48	−0.27	[−0.72; 0.17]	0.9%
Ustuner Top 2021	37	3.82	1.20	40	6.96	2.08	−1.81	[−2.35; −1.28]	0.9%
van Twillert 2007	19	3.00	1.70	19	5.60	1.73	−1.49	[−2.21; −0.76]	0.7%
Walker 2014	22	44.00	16.90	21	43.00	11.90	0.07	[−0.53; 0.67]	0.8%
Walther−Larsen 2019	28	27.00	18.52	31	15.00	18.52	0.64	[0.11; 1.16]	0.9%
Wong 2020	54	1.94	1.73	54	4.00	3.53	−0.74	[−1.13; −0.35]	0.9%
Wong 2021	21	5.50	2.40	19	5.60	1.40	−0.05	[−0.67; 0.57]	0.8%
Yang 2019	24	30.00	27.05	24	30.00	27.04	0.00	[−0.57; 0.57]	0.8%
Yilmaz Yelvar 2017	22	2.52	1.80	22	4.90	3.39	−0.86	[−1.48; −0.24]	0.8%
**Random effects model**	**4064**			**3986**			**−0.64**	**[−0.76; −0.52]**	**100.0%**

Heterogeneity: $I^2 = 85\%$, $\tau^2 = 0.3700$, $p < 0.01$

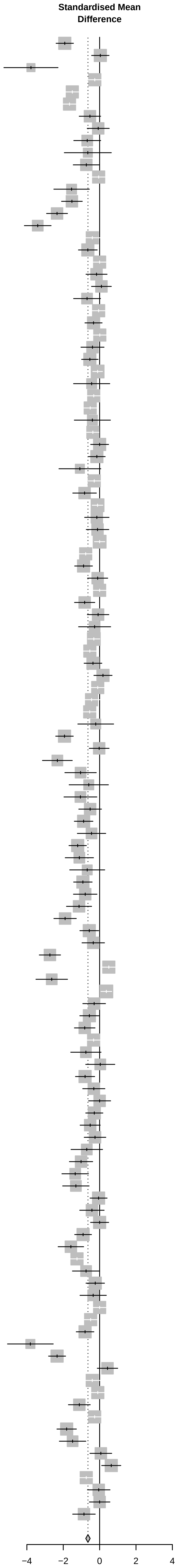

Appendix 5B: Sensitivity analysis excluding studies reporting medians

Study	Experimental Total	Experimental Mean	Experimental SD	Control Total	Control Mean	Control SD	SMD	95%−CI	Weight
Akin 2021	50	4.68	1.44	50	6.74	0.45	−1.92	[−2.39; −1.44]	0.9%
Al−Halabi 2018	33	0.75	0.98	34	0.71	0.86	0.04	[−0.44; 0.52]	0.9%
Ali 2021	11	3.10	1.04	11	7.72	1.30	−3.78	[−5.26; −2.29]	0.4%
Alshatrat 2018	50	10.00	3.88	50	11.05	3.96	−0.27	[−0.66; 0.13]	0.9%
Asl Aminabadi 2012a	58	1.89	0.65	58	3.00	0.81	−1.50	[−1.92; −1.09]	0.9%
Asl Aminabadi 2012b	59	2.05	0.60	59	3.05	0.60	−1.66	[−2.08; −1.24]	0.9%
Asvanund 2015a	23	1.57	2.29	23	3.04	3.08	−0.53	[−1.12; 0.06]	0.8%
Asvanund 2015b	21	2.23	2.29	21	2.46	3.46	−0.08	[−0.68; 0.53]	0.8%
Atzori 2018a	15	2.00	1.20	15	3.60	3.00	−0.68	[−1.42; 0.06]	0.7%
Atzori 2018b	5	2.20	1.79	5	3.80	2.59	−0.65	[−1.94; 0.64]	0.5%
Austin 2020	16	2.20	1.60	16	3.40	1.60	−0.73	[−1.45; −0.01]	0.7%
Aydin 2019	60	3.07	2.86	60	3.23	3.05	−0.05	[−0.41; 0.30]	0.9%
Azurdia 2018	11	1.82	1.60	11	4.10	1.22	−1.54	[−2.52; −0.57]	0.6%
Babaie 2019	32	26.40	7.35	30	36.80	6.02	−1.52	[−2.09; −0.95]	0.8%
Bani Mohammad 2018	40	0.33	0.82	40	4.84	2.57	−2.34	[−2.92; −1.77]	0.8%
Barad 2020	36	2.50	1.75	36	8.00	1.43	−3.40	[−4.14; −2.67]	0.7%
Basak 2020	40	3.50	2.84	40	4.72	3.15	−0.40	[−0.85; 0.04]	0.9%
Basak 2021	30	4.93	3.07	31	6.67	2.17	−0.65	[−1.16; −0.13]	0.9%
Bentsen 2001	23	31.57	24.69	23	35.74	23.55	−0.17	[−0.75; 0.41]	0.8%
Bentsen 2003	26	16.90	18.40	26	15.20	15.20	0.10	[−0.44; 0.64]	0.8%
Brown 2020	15	2.60	1.90	15	4.30	2.80	−0.69	[−1.43; 0.05]	0.7%
Buldur 2021a	38	4.00	1.85	38	4.11	2.13	−0.05	[−0.50; 0.40]	0.9%
Buldur 2021b	36	3.52	1.65	34	4.11	1.83	−0.34	[−0.81; 0.14]	0.9%
Butt 2021	55	3.58	3.10	55	3.55	3.30	0.01	[−0.36; 0.38]	0.9%
Canares 2021	19	4.13	3.88	20	5.70	3.96	−0.39	[−1.03; 0.24]	0.8%
Carrougher 2009	39	40.00	30.00	39	55.00	25.00	−0.54	[−0.99; −0.09]	0.9%
Caruso 2020	106	0.72	1.32	114	0.89	1.68	−0.11	[−0.38; 0.15]	1.0%
Chan 2007	8	38.13	34.00	8	53.75	33.38	−0.44	[−1.43; 0.56]	0.6%
Chan 2019a	64	3.08	3.29	59	4.20	3.59	−0.32	[−0.68; 0.03]	0.9%
Chan 2019b	63	2.41	3.11	66	4.21	3.76	−0.52	[−0.87; −0.17]	0.9%
Chang 2021	8	2.64	2.21	8	3.53	2.00	−0.40	[−1.39; 0.59]	0.6%
Chen 2020	68	3.35	2.38	68	4.35	2.95	−0.37	[−0.71; −0.03]	1.0%
Darnall 2020	35	3.20	3.88	39	3.80	3.96	−0.15	[−0.61; 0.31]	0.9%
Das2005	7	1.30	1.80	7	4.10	2.90	−1.09	[−2.24; 0.06]	0.5%
Deo 2020	20	3.77	2.66	20	6.00	2.62	−0.83	[−1.48; −0.18]	0.8%
Ding 2019	91	5.60	14.31	91	7.30	15.26	−0.11	[−0.41; 0.18]	1.0%
Dumoulin 2019	20	21.75	20.92	15	25.33	25.25	−0.15	[−0.82; 0.52]	0.8%
Ebrahimi 2017	20	5.35	2.68	20	5.60	1.35	−0.12	[−0.74; 0.50]	0.8%
El−Sharkawi 2012	42	1.48	1.52	42	2.76	1.78	−0.77	[−1.21; −0.32]	0.9%
Erdogan 2021	37	2.70	2.80	34	5.20	2.80	−0.88	[−1.37; −0.39]	0.9%
Felemban 2021	25	2.40	2.82	25	2.72	2.99	−0.11	[−0.66; 0.45]	0.8%
Frey 2018	27	5.60	2.00	27	7.15	1.70	−0.82	[−1.38; −0.27]	0.8%
Furman 2009a	38	1.58	1.40	38	3.66	2.00	−1.19	[−1.68; −0.70]	0.9%
Furman 2009b	38	2.39	1.80	38	3.66	2.00	−0.66	[−1.12; −0.20]	0.9%
Gershon 2004	22	13.80	20.20	22	15.40	17.00	−0.08	[−0.68; 0.51]	0.8%
Gold 2006	10	1.80	2.31	10	2.40	1.84	−0.28	[−1.16; 0.61]	0.7%
Gold 2018	70	1.31	1.59	73	1.93	2.22	−0.32	[−0.65; 0.01]	1.0%
Gold 2021	53	1.09	1.82	54	2.19	2.21	−0.54	[−0.93; −0.15]	0.9%
Goldman 2021b	32	1.84	1.94	30	1.47	2.03	0.18	[−0.32; 0.68]	0.9%
Gray 2021a	43	3.11	2.35	43	3.37	2.58	−0.10	[−0.53; 0.32]	0.9%
Gray 2021b	39	2.53	2.37	39	3.62	2.49	−0.44	[−0.89; 0.01]	0.9%
Groninger 2021	52	3.80	2.40	36	5.10	2.30	−0.55	[−0.98; −0.11]	0.9%
Guo 2014	49	2.63	1.27	49	7.64	3.41	−1.93	[−2.41; −1.45]	0.9%
Gür 2020a	54	4.98	1.69	54	6.38	1.86	−0.78	[−1.17; −0.39]	0.9%
Gür 2020b	55	4.96	1.72	54	6.38	1.86	−0.79	[−1.18; −0.40]	0.9%
Gür 2020c	55	5.96	2.05	54	6.38	1.86	−0.21	[−0.59; 0.16]	0.9%
Haisley 2020	26	3.80	3.88	26	3.90	3.96	−0.03	[−0.57; 0.52]	0.8%
Hassannia 2021	20	1.10	0.30	20	2.30	0.65	−2.32	[−3.14; −1.50]	0.7%
Hoffman 2000	12	14.67	15.24	12	36.33	23.73	−1.05	[−1.91; −0.19]	0.7%
Hoffman 2001	7	5.00	38.83	7	30.00	39.57	−0.60	[−1.68; 0.48]	0.6%
Hoffman 2008	11	5.10	2.60	11	7.60	1.90	−1.06	[−1.96; −0.15]	0.6%
Hoxhallari 2019	21	0.14	0.47	20	0.60	1.14	−0.52	[−1.15; 0.10]	0.8%
Hua 2015	33	2.42	1.85	32	4.19	2.12	−0.88	[−1.39; −0.37]	0.9%
Hwang 2014	13	5.08	2.02	13	6.15	2.60	−0.45	[−1.22; 0.33]	0.7%
Inangil 2020	40	1.30	2.15	40	4.95	3.65	−1.21	[−1.69; −0.73]	0.9%
JahaniShoorab 2015	15	16.70	16.50	15	39.30	22.50	−1.11	[−1.89; −0.34]	0.7%
Jeffs 2014	8	28.70	15.70	10	38.40	11.60	−0.68	[−1.65; 0.28]	0.6%
Jin 2016	33	36.90	18.60	33	53.10	16.10	−0.92	[−1.43; −0.41]	0.9%
Jin 2018	20	3.87	0.55	20	4.42	0.79	−0.79	[−1.44; −0.15]	0.8%
Joo 2021	19	3.70	1.40	19	5.50	1.70	−1.13	[−1.82; −0.44]	0.8%
Karaman 2021	30	2.86	1.22	30	6.33	2.24	−1.90	[−2.52; −1.28]	0.8%
Karaveli Çakir 2021	30	2.76	1.25	30	3.76	2.11	−0.57	[−1.09; −0.05]	0.9%
Kipping 2012	20	3.90	3.88	21	5.30	3.96	−0.35	[−0.97; 0.27]	0.8%
Koc Özkan 2019	46	1.97	1.20	43	6.81	2.20	−2.73	[−3.32; −2.15]	0.8%
Konstantatos 2009	43	7.30	3.88	43	5.30	3.96	0.51	[0.08; 0.94]	0.9%
Koushali 2017	20	5.50	1.30	20	8.40	0.80	−2.63	[−3.50; −1.77]	0.7%
Le May 2021	20	3.80	3.40	20	4.80	3.10	−0.30	[−0.92; 0.32]	0.8%
Litwin 2021	31	2.60	2.10	27	3.80	2.10	−0.56	[−1.09; −0.04]	0.9%
Liu 2020	30	0.80	1.06	23	2.26	2.38	−0.82	[−1.39; −0.25]	0.8%
Luczak 2021	52	4.30	2.20	51	5.10	2.60	−0.33	[−0.72; 0.06]	0.9%
Maani 2011	12	4.50	1.95	12	6.25	2.49	−0.76	[−1.59; 0.08]	0.7%
McSherry 2018	12	5.80	2.90	12	5.70	2.60	0.04	[−0.77; 0.84]	0.7%
Melcer 2021	30	2.50	1.50	30	3.80	1.70	−0.80	[−1.33; −0.27]	0.9%
Mitrakul 2015a	21	0.86	1.49	21	1.62	2.94	−0.32	[−0.93; 0.29]	0.8%
Mitrakul 2015b	21	1.90	2.93	21	1.90	3.32	0.00	[−0.60; 0.60]	0.8%
Momenyan 2021	26	9.01	0.78	26	9.37	0.57	−0.52	[−1.07; 0.03]	0.8%
Morales Tejera 2020	22	2.67	1.91	22	3.11	1.47	−0.25	[−0.85; 0.34]	0.8%
Mukherjee 2020	22	1.50	0.80	21	2.71	1.45	−1.02	[−1.66; −0.38]	0.8%
Niharika 2018a	18	2.56	1.65	18	5.22	2.18	−1.34	[−2.07; −0.61]	0.7%
Niharika 2018b	18	2.33	1.57	18	5.44	2.90	−1.31	[−2.03; −0.58]	0.7%
Nunna 2019	35	2.94	1.76	35	3.06	2.31	−0.06	[−0.53; 0.41]	0.9%
Nusser 2021	17	2.20	1.20	18	3.20	3.00	−0.42	[−1.09; 0.25]	0.8%
Ozalp Gerceker 2018	40	1.50	3.88	40	5.10	3.96	−0.91	[−1.37; −0.45]	0.9%
Ozalp Gerceker 2020a	45	1.20	2.20	46	4.10	3.50	−0.98	[−1.42; −0.55]	0.9%
Ozalp Gerceker 2020b	45	1.00	1.50	46	4.10	3.50	−1.14	[−1.58; −0.69]	0.9%
Ozalp Gerceker 2021	21	2.40	1.80	21	5.30	1.80	−1.58	[−2.28; −0.88]	0.8%
Powers 2021a	34	3.03	2.30	34	6.37	2.23	−1.46	[−2.00; −0.92]	0.9%
Powers 2021b	35	2.77	2.13	34	6.37	2.23	−1.63	[−2.18; −1.08]	0.8%
Ran 2021	60	1.58	1.08	60	2.86	0.96	−1.24	[−1.64; −0.85]	0.9%
Rousseaux 2021a	15	5.50	3.88	22	4.50	3.96	0.25	[−0.41; 0.91]	0.8%
Rousseaux 2021b	15	4.00	3.88	22	4.50	3.96	−0.12	[−0.78; 0.53]	0.8%
Sarig Bahat 2015	30	22.10	24.10	30	27.72	21.90	−0.24	[−0.75; 0.27]	0.9%
Sarig Bahat 2017	16	31.10	23.60	14	39.45	22.00	−0.36	[−1.08; 0.37]	0.7%
Schmitt 2011	54	40.00	30.86	54	55.00	29.39	−0.49	[−0.88; −0.11]	0.9%
Semerci 2020	35	2.34	3.27	36	5.02	3.35	−0.80	[−1.29; −0.32]	0.9%
Sharifpour 2020	15	40.47	4.45	15	62.80	6.73	−3.81	[−5.07; −2.55]	0.5%
Shetty 2019	60	2.42	1.47	60	5.60	1.22	−2.34	[−2.81; −1.87]	0.9%
Smith 2019	25	60.68	21.10	25	49.76	28.00	0.43	[−0.13; 0.99]	0.8%
Soltani 2018	39	52.90	27.80	39	64.40	28.30	−0.41	[−0.85; 0.04]	0.9%
Spiegel 2019	61	4.70	3.88	59	5.10	3.96	−0.10	[−0.46; 0.26]	0.9%
Sweta 2019	25	1.28	0.89	25	2.60	1.38	−1.12	[−1.72; −0.52]	0.8%
Tanja−Dijkstra 2018a	20	2.05	2.63	28	2.63	2.32	−0.23	[−0.81; 0.34]	0.8%
Tanja−Dijkstra 2018b	22	0.63	0.93	28	2.63	2.32	−1.07	[−1.67; −0.47]	0.8%
Tennant 2020	61	10.05	16.43	29	14.76	18.48	−0.27	[−0.72; 0.17]	0.9%
Ustuner Top 2021	37	3.82	1.20	40	6.96	2.08	−1.81	[−2.35; −1.28]	0.9%
van Twillert 2007	19	3.00	1.70	19	5.60	1.73	−1.49	[−2.21; −0.76]	0.7%
Walker 2014	22	44.00	16.90	21	43.00	11.90	0.07	[−0.53; 0.67]	0.8%
Wong 2020	54	1.94	1.73	54	4.00	3.53	−0.74	[−1.13; −0.35]	0.9%
Wong 2021	21	5.50	2.40	19	5.60	1.40	−0.05	[−0.67; 0.57]	0.8%
Xiang 2021a	31	24.90	34.62	29	47.10	39.57	−0.59	[−1.11; −0.07]	0.9%
Xiang 2021b	30	37.00	38.83	29	47.10	39.57	−0.25	[−0.77; 0.26]	0.9%
Yilmaz Yelvar 2017	22	2.52	1.80	22	4.90	3.39	−0.86	[−1.48; −0.24]	0.8%
**Random effects model**	**3955**			**3917**			**−0.72**	**[−0.84; −0.61]**	**100.0%**

Heterogeneity: $I^2 = 83\%$, $\tau^2 = 0.3494$, $p < 0.01$

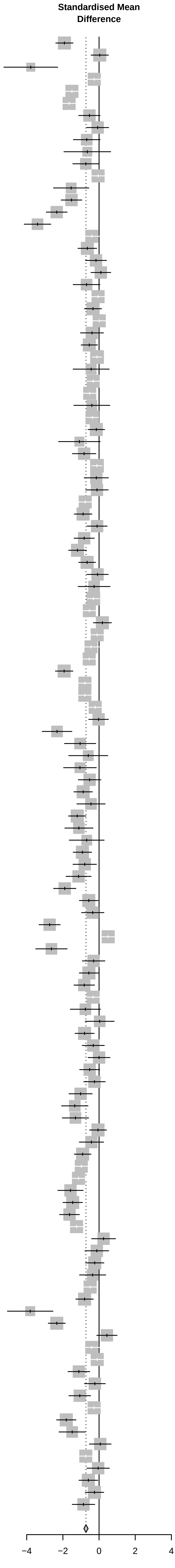

Appendix 5C: Sensitivity analysis excluding studies with imputed standard deviations

Study	Experimental Total	Experimental Mean	Experimental SD	Control Total	Control Mean	Control SD	SMD	95%−CI	Weight
Akin 2021	50	4.68	1.44	50	6.74	0.45	−1.92	[−2.39; −1.44]	0.9%
Al−Halabi 2018	33	0.75	0.98	34	0.71	0.86	0.04	[−0.44; 0.52]	0.9%
Ali 2021	11	3.10	1.04	11	7.72	1.30	−3.78	[−5.26; −2.29]	0.4%
Asl Aminabadi 2012a	58	1.89	0.65	58	3.00	0.81	−1.50	[−1.92; −1.09]	0.9%
Asl Aminabadi 2012b	59	2.05	0.60	59	3.05	0.60	−1.66	[−2.08; −1.24]	0.9%
Asvanund 2015a	23	1.57	2.29	23	3.04	3.08	−0.53	[−1.12; 0.06]	0.8%
Asvanund 2015b	21	2.23	2.29	21	2.46	3.46	−0.08	[−0.68; 0.53]	0.8%
Atzori 2018a	15	2.00	1.20	15	3.60	3.00	−0.68	[−1.42; 0.06]	0.7%
Atzori 2018b	5	2.20	1.79	5	3.80	2.59	−0.65	[−1.94; 0.64]	0.5%
Austin 2020	16	2.20	1.60	16	3.40	1.60	−0.73	[−1.45; −0.01]	0.7%
Aydin 2019	60	3.07	2.86	60	3.23	3.05	−0.05	[−0.41; 0.30]	0.9%
Azurdia 2018	11	1.82	1.60	11	4.10	1.22	−1.54	[−2.52; −0.57]	0.6%
Babaie 2019	32	26.40	7.35	30	36.80	6.02	−1.52	[−2.09; −0.95]	0.8%
Bani Mohammad 2018	40	0.33	0.82	40	4.84	2.57	−2.34	[−2.92; −1.77]	0.8%
Barad 2020	36	2.50	1.75	36	8.00	1.43	−3.40	[−4.14; −2.67]	0.7%
Basak 2020	40	3.50	2.84	40	4.72	3.15	−0.40	[−0.85; 0.04]	0.9%
Basak 2021	30	4.93	3.07	31	6.67	2.17	−0.65	[−1.16; −0.13]	0.8%
Bekelis 2017	64	30.00	11.11	63	30.00	7.41	0.00	[−0.35; 0.35]	0.9%
Bentsen 2001	23	31.57	24.69	23	35.74	23.55	−0.17	[−0.75; 0.41]	0.8%
Bentsen 2003	26	16.90	18.40	26	15.20	15.20	0.10	[−0.44; 0.64]	0.8%
Brown 2020	15	2.60	1.90	15	4.30	2.80	−0.69	[−1.43; 0.05]	0.7%
Buldur 2021a	38	4.00	1.85	38	4.11	2.13	−0.05	[−0.50; 0.40]	0.9%
Buldur 2021b	36	3.52	1.65	34	4.11	1.83	−0.34	[−0.81; 0.14]	0.9%
Butt 2021	55	3.58	3.10	55	3.55	3.30	0.01	[−0.36; 0.38]	0.9%
Carrougher 2009	39	40.00	30.00	39	55.00	25.00	−0.54	[−0.99; −0.09]	0.9%
Caruso 2020	106	0.72	1.32	114	0.89	1.68	−0.11	[−0.38; 0.15]	0.9%
Chan 2007	8	38.13	34.00	8	53.75	33.38	−0.44	[−1.43; 0.56]	0.6%
Chan 2019a	64	3.08	3.29	59	4.20	3.59	−0.32	[−0.68; 0.03]	0.9%
Chan 2019b	63	2.41	3.11	66	4.21	3.76	−0.52	[−0.87; −0.17]	0.9%
Chang 2021	8	2.64	2.21	8	3.53	2.00	−0.40	[−1.39; 0.59]	0.6%
Chen 2020	68	3.35	2.38	68	4.35	2.95	−0.37	[−0.71; −0.03]	0.9%
Clerc 2021	35	0.00	2.70	29	0.00	5.40	0.00	[−0.49; 0.49]	0.8%
Das2005	7	1.30	1.80	7	4.10	2.90	−1.09	[−2.24; 0.06]	0.5%
De Silva 2016	67	4.00	2.96	67	5.00	3.70	−0.30	[−0.64; 0.04]	0.9%
Deo 2020	20	3.77	2.66	20	6.00	2.62	−0.83	[−1.48; −0.18]	0.8%
Ding 2019	91	5.60	14.31	91	7.30	15.26	−0.11	[−0.41; 0.18]	0.9%
Dumoulin 2019	20	21.75	20.92	15	25.33	25.25	−0.15	[−0.82; 0.52]	0.8%
Ebrahimi 2017	20	5.35	2.68	20	5.60	1.35	−0.12	[−0.74; 0.50]	0.8%
Eijlers 2019	94	2.00	2.96	97	2.00	1.85	0.00	[−0.28; 0.28]	0.9%
El−Sharkawi 2012	42	1.48	1.52	42	2.76	1.78	−0.77	[−1.21; −0.32]	0.9%
Erdogan 2021	37	2.70	2.80	34	5.20	2.80	−0.88	[−1.37; −0.39]	0.9%
Felemban 2021	25	2.40	2.82	25	2.72	2.99	−0.11	[−0.66; 0.45]	0.8%
Fouks 2021	44	5.00	6.75	38	5.00	5.67	0.00	[−0.43; 0.43]	0.9%
Frey 2018	27	5.60	2.00	27	7.15	1.70	−0.82	[−1.38; −0.27]	0.8%
Furman 2009a	38	1.58	1.40	38	3.66	2.00	−1.19	[−1.68; −0.70]	0.9%
Furman 2009b	38	2.39	1.80	38	3.66	2.00	−0.66	[−1.12; −0.20]	0.9%
Gershon 2004	22	13.80	20.20	22	15.40	17.00	−0.08	[−0.68; 0.51]	0.8%
Gold 2006	10	1.80	2.31	10	2.40	1.84	−0.28	[−1.16; 0.61]	0.6%
Gold 2018	70	1.31	1.59	73	1.93	2.22	−0.32	[−0.65; 0.01]	0.9%
Gold 2021	53	1.09	1.82	54	2.19	2.21	−0.54	[−0.93; −0.15]	0.9%
Goldman 2021a	35	2.00	5.40	31	4.00	5.40	−0.37	[−0.85; 0.12]	0.9%
Goldman 2021b	32	1.84	1.94	30	1.47	2.03	0.18	[−0.32; 0.68]	0.8%
Gray 2021a	43	3.11	2.35	43	3.37	2.58	−0.10	[−0.53; 0.32]	0.9%
Gray 2021b	39	2.53	2.37	39	3.62	2.49	−0.44	[−0.89; 0.01]	0.9%
Groninger 2021	52	3.80	2.40	36	5.10	2.30	−0.55	[−0.98; −0.11]	0.9%
Gulsen 2020	8	3.95	1.59	8	4.50	3.04	−0.21	[−1.20; 0.77]	0.6%
Guo 2014	49	2.63	1.27	49	7.64	3.41	−1.93	[−2.41; −1.45]	0.9%
Gür 2020a	54	4.98	1.69	54	6.38	1.86	−0.78	[−1.17; −0.39]	0.9%
Gür 2020b	55	4.96	1.72	54	6.38	1.86	−0.79	[−1.18; −0.40]	0.9%
Gür 2020c	55	5.96	2.05	54	6.38	1.86	−0.21	[−0.59; 0.16]	0.9%
Hassannia 2021	20	1.10	0.30	20	2.30	0.65	−2.32	[−3.14; −1.50]	0.7%
Hoffman 2000	12	14.67	15.24	12	36.33	23.73	−1.05	[−1.91; −0.19]	0.6%
Hoffman 2008	11	5.10	2.60	11	7.60	1.90	−1.06	[−1.96; −0.15]	0.6%
Hoxhallari 2019	21	0.14	0.47	20	0.60	1.14	−0.52	[−1.15; 0.10]	0.8%
Hua 2015	33	2.42	1.85	32	4.19	2.12	−0.88	[−1.39; −0.37]	0.8%
Hwang 2014	13	5.08	2.02	13	6.15	2.60	−0.45	[−1.22; 0.33]	0.7%
Inangil 2020	40	1.30	2.15	40	4.95	3.65	−1.21	[−1.69; −0.73]	0.9%
JahaniShoorab 2015	15	16.70	16.50	15	39.30	22.50	−1.11	[−1.89; −0.34]	0.7%
Jeffs 2014	8	28.70	15.70	10	38.40	11.60	−0.68	[−1.65; 0.28]	0.6%
Jin 2016	33	36.90	18.60	33	53.10	16.10	−0.92	[−1.43; −0.41]	0.8%
Jin 2018	20	3.87	0.55	20	4.42	0.79	−0.79	[−1.44; −0.15]	0.8%
Joo 2021	19	3.70	1.40	19	5.50	1.70	−1.13	[−1.82; −0.44]	0.7%
Karaman 2021	30	2.86	1.22	30	6.33	2.24	−1.90	[−2.52; −1.28]	0.8%
Karaveli Çakir 2021	30	2.76	1.25	30	3.76	2.11	−0.57	[−1.09; −0.05]	0.8%
Koc Özkan 2019	46	1.97	1.20	43	6.81	2.20	−2.73	[−3.32; −2.15]	0.8%
Koushali 2017	20	5.50	1.30	20	8.40	0.80	−2.63	[−3.50; −1.77]	0.6%
Laghlam 2021	90	5.00	5.40	90	3.00	5.40	0.37	[0.07; 0.66]	0.9%
Le May 2021	20	3.80	3.40	20	4.80	3.10	−0.30	[−0.92; 0.32]	0.8%
Litwin 2021	31	2.60	2.10	27	3.80	2.10	−0.56	[−1.09; −0.04]	0.8%
Liu 2020	30	0.80	1.06	23	2.26	2.38	−0.82	[−1.39; −0.25]	0.8%
Luczak 2021	52	4.30	2.20	51	5.10	2.60	−0.33	[−0.72; 0.06]	0.9%
Maani 2011	12	4.50	1.95	12	6.25	2.49	−0.76	[−1.59; 0.08]	0.7%
McSherry 2018	12	5.80	2.90	12	5.70	2.60	0.04	[−0.77; 0.84]	0.7%
Melcer 2021	30	2.50	1.50	30	3.80	1.70	−0.80	[−1.33; −0.27]	0.8%
Mitrakul 2015a	21	0.86	1.49	21	1.62	2.94	−0.32	[−0.93; 0.29]	0.8%
Mitrakul 2015b	21	1.90	2.93	21	1.90	3.32	0.00	[−0.60; 0.60]	0.8%
Mohanasundari 2021	35	3.00	1.35	35	5.00	9.45	−0.29	[−0.76; 0.18]	0.9%
Momenyan 2021	26	9.01	0.78	26	9.37	0.57	−0.52	[−1.07; 0.03]	0.8%
Morales Tejera 2020	22	2.67	1.91	22	3.11	1.47	−0.25	[−0.85; 0.34]	0.8%
Morris 2010	11	3.00	4.44	11	6.00	3.70	−0.71	[−1.57; 0.16]	0.6%
Mukherjee 2020	22	1.50	0.80	21	2.71	1.45	−1.02	[−1.66; −0.38]	0.8%
Niharika 2018a	18	2.56	1.65	18	5.22	2.18	−1.34	[−2.07; −0.61]	0.7%
Niharika 2018b	18	2.33	1.57	18	5.44	2.90	−1.31	[−2.03; −0.58]	0.7%
Nunna 2019	35	2.94	1.76	35	3.06	2.31	−0.06	[−0.53; 0.41]	0.9%
Nusser 2021	17	2.20	1.20	18	3.20	3.00	−0.42	[−1.09; 0.25]	0.8%
Osmanlliu 2020	31	3.00	6.75	31	3.00	6.08	0.00	[−0.50; 0.50]	0.8%
Ozalp Gerceker 2020a	45	1.20	2.20	46	4.10	3.50	−0.98	[−1.42; −0.55]	0.9%
Ozalp Gerceker 2020b	45	1.00	1.50	46	4.10	3.50	−1.14	[−1.58; −0.69]	0.9%
Ozalp Gerceker 2021	21	2.40	1.80	21	5.30	1.80	−1.58	[−2.28; −0.88]	0.7%
Powers 2021a	34	3.03	2.30	34	6.37	2.23	−1.46	[−2.00; −0.92]	0.8%
Powers 2021b	35	2.77	2.13	34	6.37	2.23	−1.63	[−2.18; −1.08]	0.8%
Ran 2021	60	1.58	1.08	60	2.86	0.96	−1.24	[−1.64; −0.85]	0.9%
Sarig Bahat 2015	30	22.10	24.10	30	27.72	21.90	−0.24	[−0.75; 0.27]	0.8%
Sarig Bahat 2017	16	31.10	23.60	14	39.45	22.00	−0.36	[−1.08; 0.37]	0.7%
Schlechter 2020	58	4.00	2.22	57	4.00	4.44	0.00	[−0.37; 0.37]	0.9%
Schmitt 2011	54	40.00	30.86	54	55.00	29.39	−0.49	[−0.88; −0.11]	0.9%
Semerci 2020	35	2.34	3.27	36	5.02	3.35	−0.80	[−1.29; −0.32]	0.9%
Sharifpour 2020	15	40.47	4.45	15	62.80	6.73	−3.81	[−5.07; −2.55]	0.5%
Shetty 2019	60	2.42	1.47	60	5.60	1.22	−2.34	[−2.81; −1.87]	0.9%
Smith 2019	25	60.68	21.10	25	49.76	28.00	0.43	[−0.13; 0.99]	0.8%
Soltani 2018	39	52.90	27.80	39	64.40	28.30	−0.41	[−0.85; 0.04]	0.9%
Sweta 2019	25	1.28	0.89	25	2.60	1.38	−1.12	[−1.72; −0.52]	0.8%
Tanja−Dijkstra 2018a	20	2.05	2.63	28	2.63	2.32	−0.23	[−0.81; 0.34]	0.8%
Tanja−Dijkstra 2018b	22	0.63	0.93	28	2.63	2.32	−1.07	[−1.67; −0.47]	0.8%
Tennant 2020	61	10.05	16.43	29	14.76	18.48	−0.27	[−0.72; 0.17]	0.9%
Ustuner Top 2021	37	3.82	1.20	40	6.96	2.08	−1.81	[−2.35; −1.28]	0.8%
van Twillert 2007	19	3.00	1.70	19	5.60	1.73	−1.49	[−2.21; −0.76]	0.7%
Walker 2014	22	44.00	16.90	21	43.00	11.90	0.07	[−0.53; 0.67]	0.8%
Walther−Larsen 2019	28	27.00	18.52	31	15.00	18.52	0.64	[0.11; 1.16]	0.8%
Wong 2020	54	1.94	1.73	54	4.00	3.53	−0.74	[−1.13; −0.35]	0.9%
Wong 2021	21	5.50	2.40	19	5.60	1.40	−0.05	[−0.67; 0.57]	0.8%
Xiang 2021a	31	24.90	34.62	29	47.10	39.57	−0.59	[−1.11; −0.07]	0.8%
Xiang 2021b	30	37.00	38.83	29	47.10	39.57	−0.25	[−0.77; 0.26]	0.8%
Yang 2019	24	30.00	27.05	24	30.00	27.04	0.00	[−0.57; 0.57]	0.8%
Yilmaz Yelvar 2017	22	2.52	1.80	22	4.90	3.39	−0.86	[−1.48; −0.24]	0.8%
**Random effects model**	**4248**			**4180**			**−0.69**	**[−0.81; −0.57]**	**100.0%**

Heterogeneity: $I^2 = 85\%$, $\tau^2 = 0.3624$, $p < 0.01$

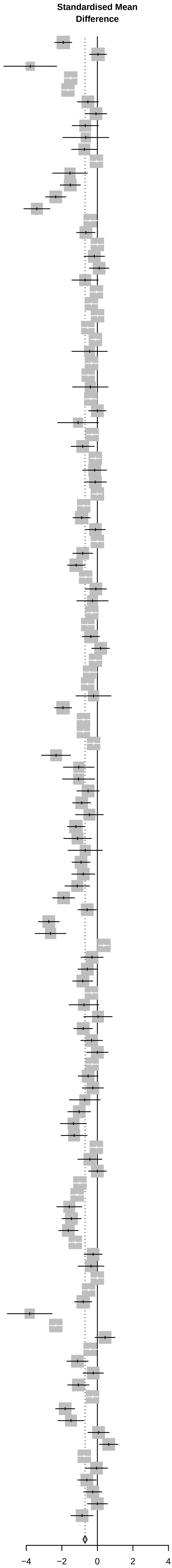

Appendix 5D: Sensitivity analysis excluding studies with high risk of bias

Study	Experimental Total	Experimental Mean	Experimental SD	Control Total	Control Mean	Control SD	SMD	95%−CI	Weight
Al−Halabi 2018	33	0.75	0.98	34	0.71	0.86	0.04	[−0.44; 0.52]	1.0%
Ali 2021	11	3.10	1.04	11	7.72	1.30	−3.78	[−5.26; −2.29]	0.4%
Asl Aminabadi 2012a	58	1.89	0.65	58	3.00	0.81	−1.50	[−1.92; −1.09]	1.0%
Asl Aminabadi 2012b	59	2.05	0.60	59	3.05	0.60	−1.66	[−2.08; −1.24]	1.0%
Asvanund 2015a	23	1.57	2.29	23	3.04	3.08	−0.53	[−1.12; 0.06]	0.9%
Asvanund 2015b	21	2.23	2.29	21	2.46	3.46	−0.08	[−0.68; 0.53]	0.9%
Atzori 2018a	15	2.00	1.20	15	3.60	3.00	−0.68	[−1.42; 0.06]	0.8%
Atzori 2018b	5	2.20	1.79	5	3.80	2.59	−0.65	[−1.94; 0.64]	0.5%
Austin 2020	16	2.20	1.60	16	3.40	1.60	−0.73	[−1.45; −0.01]	0.9%
Azurdia 2018	11	1.82	1.60	11	4.10	1.22	−1.54	[−2.52; −0.57]	0.7%
Babaie 2019	32	26.40	7.35	30	36.80	6.02	−1.52	[−2.09; −0.95]	0.9%
Basak 2021	30	4.93	3.07	31	6.67	2.17	−0.65	[−1.16; −0.13]	1.0%
Bekelis 2017	64	30.00	11.11	63	30.00	7.41	0.00	[−0.35; 0.35]	1.1%
Bentsen 2003	26	16.90	18.40	26	15.20	15.20	0.10	[−0.44; 0.64]	1.0%
Brown 2020	15	2.60	1.90	15	4.30	2.80	−0.69	[−1.43; 0.05]	0.8%
Buldur 2021a	38	4.00	1.85	38	4.11	2.13	−0.05	[−0.50; 0.40]	1.0%
Buldur 2021b	36	3.52	1.65	34	4.11	1.83	−0.34	[−0.81; 0.14]	1.0%
Butt 2021	55	3.58	3.10	55	3.55	3.30	0.01	[−0.36; 0.38]	1.1%
Canares 2021	19	4.13	3.88	20	5.70	3.96	−0.39	[−1.03; 0.24]	0.9%
Carrougher 2009	39	40.00	30.00	39	55.00	25.00	−0.54	[−0.99; −0.09]	1.0%
Chan 2007	8	38.13	34.00	8	53.75	33.38	−0.44	[−1.43; 0.56]	0.7%
Chan 2019a	64	3.08	3.29	59	4.20	3.59	−0.32	[−0.68; 0.03]	1.1%
Chan 2019b	63	2.41	3.11	66	4.21	3.76	−0.52	[−0.87; −0.17]	1.1%
Chang 2021	8	2.64	2.21	8	3.53	2.00	−0.40	[−1.39; 0.59]	0.7%
Chen 2020	68	3.35	2.38	68	4.35	2.95	−0.37	[−0.71; −0.03]	1.1%
Clerc 2021	35	0.00	2.70	29	0.00	5.40	0.00	[−0.49; 0.49]	1.0%
De Silva 2016	67	4.00	2.96	67	5.00	3.70	−0.30	[−0.64; 0.04]	1.1%
Deo 2020	20	3.77	2.66	20	6.00	2.62	−0.83	[−1.48; −0.18]	0.9%
Ding 2019	91	5.60	14.31	91	7.30	15.26	−0.11	[−0.41; 0.18]	1.1%
Dumoulin 2019	20	21.75	20.92	15	25.33	25.25	−0.15	[−0.82; 0.52]	0.9%
Ebrahimi 2017	20	5.35	2.68	20	5.60	1.35	−0.12	[−0.74; 0.50]	0.9%
Eijlers 2019	94	2.00	2.96	97	2.00	1.85	0.00	[−0.28; 0.28]	1.1%
Erdogan 2021	37	2.70	2.80	34	5.20	2.80	−0.88	[−1.37; −0.39]	1.0%
Felemban 2021	25	2.40	2.82	25	2.72	2.99	−0.11	[−0.66; 0.45]	1.0%
Fouks 2021	44	5.00	6.75	38	5.00	5.67	0.00	[−0.43; 0.43]	1.0%
Gershon 2004	22	13.80	20.20	22	15.40	17.00	−0.08	[−0.68; 0.51]	0.9%
Gold 2006	10	1.80	2.31	10	2.40	1.84	−0.28	[−1.16; 0.61]	0.7%
Gold 2018	70	1.31	1.59	73	1.93	2.22	−0.32	[−0.65; 0.01]	1.1%
Gold 2021	53	1.09	1.82	54	2.19	2.21	−0.54	[−0.93; −0.15]	1.1%
Goldman 2021b	32	1.84	1.94	30	1.47	2.03	0.18	[−0.32; 0.68]	1.0%
Groninger 2021	52	3.80	2.40	36	5.10	2.30	−0.55	[−0.98; −0.11]	1.0%
Guo 2014	49	2.63	1.27	49	7.64	3.41	−1.93	[−2.41; −1.45]	1.0%
Gür 2020a	54	4.98	1.69	54	6.38	1.86	−0.78	[−1.17; −0.39]	1.1%
Gür 2020b	55	4.96	1.72	54	6.38	1.86	−0.79	[−1.18; −0.40]	1.1%
Gür 2020c	55	5.96	2.05	54	6.38	1.86	−0.21	[−0.59; 0.16]	1.1%
Haisley 2020	26	3.80	3.88	26	3.90	3.96	−0.03	[−0.57; 0.52]	1.0%
Hassannia 2021	20	1.10	0.30	20	2.30	0.65	−2.32	[−3.14; −1.50]	0.8%
Hua 2015	33	2.42	1.85	32	4.19	2.12	−0.88	[−1.39; −0.37]	1.0%
Hwang 2014	13	5.08	2.02	13	6.15	2.60	−0.45	[−1.22; 0.33]	0.8%
Inangil 2020	40	1.30	2.15	40	4.95	3.65	−1.21	[−1.69; −0.73]	1.0%
JahaniShoorab 2015	15	16.70	16.50	15	39.30	22.50	−1.11	[−1.89; −0.34]	0.8%
Jeffs 2014	8	28.70	15.70	10	38.40	11.60	−0.68	[−1.65; 0.28]	0.7%
Jin 2018	20	3.87	0.55	20	4.42	0.79	−0.79	[−1.44; −0.15]	0.9%
Joo 2021	19	3.70	1.40	19	5.50	1.70	−1.13	[−1.82; −0.44]	0.9%
Karaman 2021	30	2.86	1.22	30	6.33	2.24	−1.90	[−2.52; −1.28]	0.9%
Karaveli Çakir 2021	30	2.76	1.25	30	3.76	2.11	−0.57	[−1.09; −0.05]	1.0%
Kipping 2012	20	3.90	3.88	21	5.30	3.96	−0.35	[−0.97; 0.27]	0.9%
Koc Özkan 2019	46	1.97	1.20	43	6.81	2.20	−2.73	[−3.32; −2.15]	0.9%
Konstantatos 2009	43	7.30	3.88	43	5.30	3.96	0.51	[0.08; 0.94]	1.0%
Koushali 2017	20	5.50	1.30	20	8.40	0.80	−2.63	[−3.50; −1.77]	0.8%
Laghlam 2021	90	5.00	5.40	90	3.00	5.40	0.37	[0.07; 0.66]	1.1%
Litwin 2021	31	2.60	2.10	27	3.80	2.10	−0.56	[−1.09; −0.04]	1.0%
Liu 2020	30	0.80	1.06	23	2.26	2.38	−0.82	[−1.39; −0.25]	1.0%
Luczak 2021	52	4.30	2.20	51	5.10	2.60	−0.33	[−0.72; 0.06]	1.1%
McSherry 2018	12	5.80	2.90	12	5.70	2.60	0.04	[−0.77; 0.84]	0.8%
Melcer 2021	30	2.50	1.50	30	3.80	1.70	−0.80	[−1.33; −0.27]	1.0%
Mitrakul 2015a	21	0.86	1.49	21	1.62	2.94	−0.32	[−0.93; 0.29]	0.9%
Mitrakul 2015b	21	1.90	2.93	21	1.90	3.32	0.00	[−0.60; 0.60]	0.9%
Mohanasundari 2021	35	3.00	1.35	35	5.00	9.45	−0.29	[−0.76; 0.18]	1.0%
Momenyan 2021	26	9.01	0.78	26	9.37	0.57	−0.52	[−1.07; 0.03]	1.0%
Morales Tejera 2020	22	2.67	1.91	22	3.11	1.47	−0.25	[−0.85; 0.34]	0.9%
Mukherjee 2020	22	1.50	0.80	21	2.71	1.45	−1.02	[−1.66; −0.38]	0.9%
Nunna 2019	35	2.94	1.76	35	3.06	2.31	−0.06	[−0.53; 0.41]	1.0%
Nusser 2021	17	2.20	1.20	18	3.20	3.00	−0.42	[−1.09; 0.25]	0.9%
Osmanlliu 2020	31	3.00	6.75	31	3.00	6.08	0.00	[−0.50; 0.50]	1.0%
Ozalp Gerceker 2018	40	1.50	3.88	40	5.10	3.96	−0.91	[−1.37; −0.45]	1.0%
Ozalp Gerceker 2020a	45	1.20	2.20	46	4.10	3.50	−0.98	[−1.42; −0.55]	1.0%
Ozalp Gerceker 2020b	45	1.00	1.50	46	4.10	3.50	−1.14	[−1.58; −0.69]	1.0%
Ozalp Gerceker 2021	21	2.40	1.80	21	5.30	1.80	−1.58	[−2.28; −0.88]	0.9%
Powers 2021a	34	3.03	2.30	34	6.37	2.23	−1.46	[−2.00; −0.92]	1.0%
Powers 2021b	35	2.77	2.13	34	6.37	2.23	−1.63	[−2.18; −1.08]	1.0%
Ran 2021	60	1.58	1.08	60	2.86	0.96	−1.24	[−1.64; −0.85]	1.1%
Rousseaux 2021a	15	5.50	3.88	22	4.50	3.96	0.25	[−0.41; 0.91]	0.9%
Rousseaux 2021b	15	4.00	3.88	22	4.50	3.96	−0.12	[−0.78; 0.53]	0.9%
Sander Wint 2002	17	3.00	3.88	13	6.00	3.96	−0.75	[−1.50; 0.00]	0.8%
Sarig Bahat 2015	30	22.10	24.10	30	27.72	21.90	−0.24	[−0.75; 0.27]	1.0%
Sarig Bahat 2017	16	31.10	23.60	14	39.45	22.00	−0.36	[−1.08; 0.37]	0.8%
Schlechter 2020	58	4.00	2.22	57	4.00	4.44	0.00	[−0.37; 0.37]	1.1%
Semerci 2020	35	2.34	3.27	36	5.02	3.35	−0.80	[−1.29; −0.32]	1.0%
Sharifpour 2020	15	40.47	4.45	15	62.80	6.73	−3.81	[−5.07; −2.55]	0.5%
Shetty 2019	60	2.42	1.47	60	5.60	1.22	−2.34	[−2.81; −1.87]	1.0%
Smith 2019	25	60.68	21.10	25	49.76	28.00	0.43	[−0.13; 0.99]	1.0%
Sweta 2019	25	1.28	0.89	25	2.60	1.38	−1.12	[−1.72; −0.52]	0.9%
Tanja−Dijkstra 2018a	20	2.05	2.63	28	2.63	2.32	−0.23	[−0.81; 0.34]	0.9%
Tanja−Dijkstra 2018b	22	0.63	0.93	28	2.63	2.32	−1.07	[−1.67; −0.47]	0.9%
Tennant 2020	61	10.05	16.43	29	14.76	18.48	−0.27	[−0.72; 0.17]	1.0%
Ustuner Top 2021	37	3.82	1.20	40	6.96	2.08	−1.81	[−2.35; −1.28]	1.0%
van Twillert 2007	19	3.00	1.70	19	5.60	1.73	−1.49	[−2.21; −0.76]	0.8%
Walker 2014	22	44.00	16.90	21	43.00	11.90	0.07	[−0.53; 0.67]	0.9%
Walther−Larsen 2019	28	27.00	18.52	31	15.00	18.52	0.64	[0.11; 1.16]	1.0%
Wong 2020	54	1.94	1.73	54	4.00	3.53	−0.74	[−1.13; −0.35]	1.1%
Wong 2021	21	5.50	2.40	19	5.60	1.40	−0.05	[−0.67; 0.57]	0.9%
Xiang 2021a	31	24.90	34.62	29	47.10	39.57	−0.59	[−1.11; −0.07]	1.0%
Xiang 2021b	30	37.00	38.83	29	47.10	39.57	−0.25	[−0.77; 0.26]	1.0%
Yang 2019	24	30.00	27.05	24	30.00	27.04	0.00	[−0.57; 0.57]	1.0%
Yilmaz Yelvar 2017	22	2.52	1.80	22	4.90	3.39	−0.86	[−1.48; −0.24]	0.9%
**Random effects model**	**3562**			**3503**			**−0.62**	**[−0.75; −0.50]**	**100.0%**

Heterogeneity: I^2 = 84%, τ^2 = 0.3547, p < 0.01

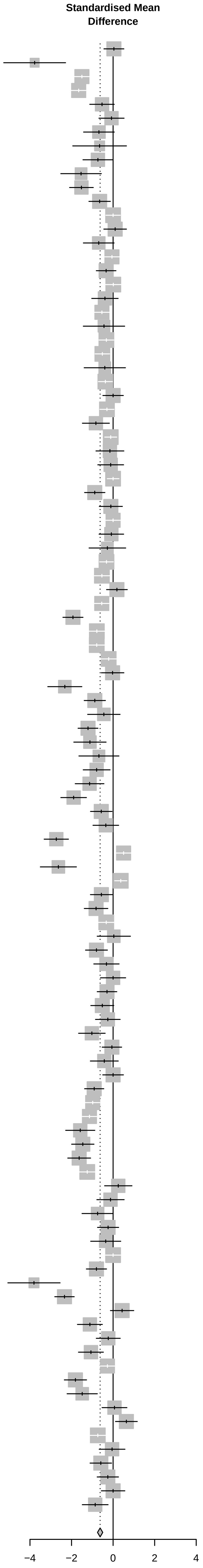

Appendix 5E: Sensitivity analysis excluding studies reporting worst pain scores

Study	Experimental Total	Experimental Mean	Experimental SD	Control Total	Control Mean	Control SD	SMD	95%−CI	Weight
Akin 2021	50	4.68	1.44	50	6.74	0.45	−1.92	[−2.39; −1.44]	0.8%
Al−Halabi 2018	33	0.75	0.98	34	0.71	0.86	0.04	[−0.44; 0.52]	0.8%
Ali 2021	11	3.10	1.04	11	7.72	1.30	−3.78	[−5.26; −2.29]	0.4%
Alshatrat 2018	50	10.00	3.88	50	11.05	3.96	−0.27	[−0.66; 0.13]	0.9%
Asl Aminabadi 2012a	58	1.89	0.65	58	3.00	0.81	−1.50	[−1.92; −1.09]	0.9%
Asl Aminabadi 2012b	59	2.05	0.60	59	3.05	0.60	−1.66	[−2.08; −1.24]	0.9%
Asvanund 2015a	23	1.57	2.29	23	3.04	3.08	−0.53	[−1.12; 0.06]	0.8%
Asvanund 2015b	21	2.23	2.29	21	2.46	3.46	−0.08	[−0.68; 0.53]	0.8%
Austin 2020	16	2.20	1.60	16	3.40	1.60	−0.73	[−1.45; −0.01]	0.7%
Aydin 2019	60	3.07	2.86	60	3.23	3.05	−0.05	[−0.41; 0.30]	0.9%
Azurdia 2018	11	1.82	1.60	11	4.10	1.22	−1.54	[−2.52; −0.57]	0.6%
Babaie 2019	32	26.40	7.35	30	36.80	6.02	−1.52	[−2.09; −0.95]	0.8%
Bani Mohammad 2018	40	0.33	0.82	40	4.84	2.57	−2.34	[−2.92; −1.77]	0.8%
Barad 2020	36	2.50	1.75	36	8.00	1.43	−3.40	[−4.14; −2.67]	0.7%
Basak 2020	40	3.50	2.84	40	4.72	3.15	−0.40	[−0.85; 0.04]	0.9%
Basak 2021	30	4.93	3.07	31	6.67	2.17	−0.65	[−1.16; −0.13]	0.8%
Bekelis 2017	64	30.00	11.11	63	30.00	7.41	0.00	[−0.35; 0.35]	0.9%
Bentsen 2001	23	31.57	24.69	23	35.74	23.55	−0.17	[−0.75; 0.41]	0.8%
Bentsen 2003	26	16.90	18.40	26	15.20	15.20	0.10	[−0.44; 0.64]	0.8%
Brown 2020	15	2.60	1.90	15	4.30	2.80	−0.69	[−1.43; 0.05]	0.7%
Buldur 2021a	38	4.00	1.85	38	4.11	2.13	−0.05	[−0.50; 0.40]	0.9%
Buldur 2021b	36	3.52	1.65	34	4.11	1.83	−0.34	[−0.81; 0.14]	0.8%
Butt 2021	55	3.58	3.10	55	3.55	3.30	0.01	[−0.36; 0.38]	0.9%
Caruso 2020	106	0.72	1.32	114	0.89	1.68	−0.11	[−0.38; 0.15]	0.9%
Chan 2007	8	38.13	34.00	8	53.75	33.38	−0.44	[−1.43; 0.56]	0.6%
Chan 2019a	64	3.08	3.29	59	4.20	3.59	−0.32	[−0.68; 0.03]	0.9%
Chan 2019b	63	2.41	3.11	66	4.21	3.76	−0.52	[−0.87; −0.17]	0.9%
Chang 2021	8	2.64	2.21	8	3.53	2.00	−0.40	[−1.39; 0.59]	0.6%
Chen 2020	68	3.35	2.38	68	4.35	2.95	−0.37	[−0.71; −0.03]	0.9%
Clerc 2021	35	0.00	2.70	29	0.00	5.40	0.00	[−0.49; 0.49]	0.8%
Darnall 2020	35	3.20	3.88	39	3.80	3.96	−0.15	[−0.61; 0.31]	0.9%
Das2005	7	1.30	1.80	7	4.10	2.90	−1.09	[−2.24; 0.06]	0.5%
De Silva 2016	67	4.00	2.96	67	5.00	3.70	−0.30	[−0.64; 0.04]	0.9%
Deo 2020	20	3.77	2.66	20	6.00	2.62	−0.83	[−1.48; −0.18]	0.8%
Ding 2019	91	5.60	14.31	91	7.30	15.26	−0.11	[−0.41; 0.18]	0.9%
Dumoulin 2019	20	21.75	20.92	15	25.33	25.25	−0.15	[−0.82; 0.52]	0.7%
Ebrahimi 2017	20	5.35	2.68	20	5.60	1.35	−0.12	[−0.74; 0.50]	0.8%
Eijlers 2019	94	2.00	2.96	97	2.00	1.85	0.00	[−0.28; 0.28]	0.9%
El−Sharkawi 2012	42	1.48	1.52	42	2.76	1.78	−0.77	[−1.21; −0.32]	0.9%
Erdogan 2021	37	2.70	2.80	34	5.20	2.80	−0.88	[−1.37; −0.39]	0.8%
Felemban 2021	25	2.40	2.82	25	2.72	2.99	−0.11	[−0.66; 0.45]	0.8%
Fouks 2021	44	5.00	6.75	38	5.00	5.67	0.00	[−0.43; 0.43]	0.9%
Furman 2009a	38	1.58	1.40	38	3.66	2.00	−1.19	[−1.68; −0.70]	0.8%
Furman 2009b	38	2.39	1.80	38	3.66	2.00	−0.66	[−1.12; −0.20]	0.9%
Gershon 2004	22	13.80	20.20	22	15.40	17.00	−0.08	[−0.68; 0.51]	0.8%
Gold 2006	10	1.80	2.31	10	2.40	1.84	−0.28	[−1.16; 0.61]	0.6%
Gold 2018	70	1.31	1.59	73	1.93	2.22	−0.32	[−0.65; 0.01]	0.9%
Gold 2021	53	1.09	1.82	54	2.19	2.21	−0.54	[−0.93; −0.15]	0.9%
Goldman 2021a	35	2.00	5.40	31	4.00	5.40	−0.37	[−0.85; 0.12]	0.8%
Goldman 2021b	32	1.84	1.94	30	1.47	2.03	0.18	[−0.32; 0.68]	0.8%
Gray 2021a	43	3.11	2.35	43	3.37	2.58	−0.10	[−0.53; 0.32]	0.9%
Gray 2021b	39	2.53	2.37	39	3.62	2.49	−0.44	[−0.89; 0.01]	0.9%
Groninger 2021	52	3.80	2.40	36	5.10	2.30	−0.55	[−0.98; −0.11]	0.9%
Gulsen 2020	8	3.95	1.59	8	4.50	3.04	−0.21	[−1.20; 0.77]	0.6%
Guo 2014	49	2.63	1.27	49	7.64	3.41	−1.93	[−2.41; −1.45]	0.8%
Gür 2020a	54	4.98	1.69	54	6.38	1.86	−0.78	[−1.17; −0.39]	0.9%
Gür 2020b	55	4.96	1.72	54	6.38	1.86	−0.79	[−1.18; −0.40]	0.9%
Gür 2020c	55	5.96	2.05	54	6.38	1.86	−0.21	[−0.59; 0.16]	0.9%
Haisley 2020	26	3.80	3.88	26	3.90	3.96	−0.03	[−0.57; 0.52]	0.8%
Hassannia 2021	20	1.10	0.30	20	2.30	0.65	−2.32	[−3.14; −1.50]	0.7%
Hoffman 2000	12	14.67	15.24	12	36.33	23.73	−1.05	[−1.91; −0.19]	0.6%
Hoffman 2001	7	5.00	38.83	7	30.00	39.57	−0.60	[−1.68; 0.48]	0.5%
Hoxhallari 2019	21	0.14	0.47	20	0.60	1.14	−0.52	[−1.15; 0.10]	0.8%
Hua 2015	33	2.42	1.85	32	4.19	2.12	−0.88	[−1.39; −0.37]	0.8%
Hwang 2014	13	5.08	2.02	13	6.15	2.60	−0.45	[−1.22; 0.33]	0.7%
Inangil 2020	40	1.30	2.15	40	4.95	3.65	−1.21	[−1.69; −0.73]	0.8%
JahaniShoorab 2015	15	16.70	16.50	15	39.30	22.50	−1.11	[−1.89; −0.34]	0.7%
Jeffs 2014	8	28.70	15.70	10	38.40	11.60	−0.68	[−1.65; 0.28]	0.6%
Jin 2016	33	36.90	18.60	33	53.10	16.10	−0.92	[−1.43; −0.41]	0.8%
Jin 2018	20	3.87	0.55	20	4.42	0.79	−0.79	[−1.44; −0.15]	0.8%
Joo 2021	19	3.70	1.40	19	5.50	1.70	−1.13	[−1.82; −0.44]	0.7%
Karaman 2021	30	2.86	1.22	30	6.33	2.24	−1.90	[−2.52; −1.28]	0.8%
Karaveli Çakir 2021	30	2.76	1.25	30	3.76	2.11	−0.57	[−1.09; −0.05]	0.8%
Kipping 2012	20	3.90	3.88	21	5.30	3.96	−0.35	[−0.97; 0.27]	0.8%
Koc Özkan 2019	46	1.97	1.20	43	6.81	2.20	−2.73	[−3.32; −2.15]	0.8%
Koushali 2017	20	5.50	1.30	20	8.40	0.80	−2.63	[−3.50; −1.77]	0.6%
Laghlam 2021	90	5.00	5.40	90	3.00	5.40	0.37	[0.07; 0.66]	0.9%
Le May 2021	20	3.80	3.40	20	4.80	3.10	−0.30	[−0.92; 0.32]	0.8%
Litwin 2021	31	2.60	2.10	27	3.80	2.10	−0.56	[−1.09; −0.04]	0.8%
Liu 2020	30	0.80	1.06	23	2.26	2.38	−0.82	[−1.39; −0.25]	0.8%
Luczak 2021	52	4.30	2.20	51	5.10	2.60	−0.33	[−0.72; 0.06]	0.9%
McSherry 2018	12	5.80	2.90	12	5.70	2.60	0.04	[−0.77; 0.84]	0.7%
Melcer 2021	30	2.50	1.50	30	3.80	1.70	−0.80	[−1.33; −0.27]	0.8%
Mitrakul 2015a	21	0.86	1.49	21	1.62	2.94	−0.32	[−0.93; 0.29]	0.8%
Mitrakul 2015b	21	1.90	2.93	21	1.90	3.32	0.00	[−0.60; 0.60]	0.8%
Mohanasundari 2021	35	3.00	1.35	35	5.00	9.45	−0.29	[−0.76; 0.18]	0.8%
Morales Tejera 2020	22	2.67	1.91	22	3.11	1.47	−0.25	[−0.85; 0.34]	0.8%
Morris 2010	11	3.00	4.44	11	6.00	3.70	−0.71	[−1.57; 0.16]	0.6%
Mukherjee 2020	22	1.50	0.80	21	2.71	1.45	−1.02	[−1.66; −0.38]	0.8%
Niharika 2018a	18	2.56	1.65	18	5.22	2.18	−1.34	[−2.07; −0.61]	0.7%
Niharika 2018b	18	2.33	1.57	18	5.44	2.90	−1.31	[−2.03; −0.58]	0.7%
Nunna 2019	35	2.94	1.76	35	3.06	2.31	−0.06	[−0.53; 0.41]	0.9%
Nusser 2021	17	2.20	1.20	18	3.20	3.00	−0.42	[−1.09; 0.25]	0.7%
Osmanlliu 2020	31	3.00	6.75	31	3.00	6.08	0.00	[−0.50; 0.50]	0.8%
Ozalp Gerceker 2018	40	1.50	3.88	40	5.10	3.96	−0.91	[−1.37; −0.45]	0.9%
Ozalp Gerceker 2020a	45	1.20	2.20	46	4.10	3.50	−0.98	[−1.42; −0.55]	0.9%
Ozalp Gerceker 2020b	45	1.00	1.50	46	4.10	3.50	−1.14	[−1.58; −0.69]	0.9%
Ozalp Gerceker 2021	21	2.40	1.80	21	5.30	1.80	−1.58	[−2.28; −0.88]	0.7%
Powers 2021a	34	3.03	2.30	34	6.37	2.23	−1.46	[−2.00; −0.92]	0.8%
Powers 2021b	35	2.77	2.13	34	6.37	2.23	−1.63	[−2.18; −1.08]	0.8%
Ran 2021	60	1.58	1.08	60	2.86	0.96	−1.24	[−1.64; −0.85]	0.9%
Rousseaux 2021a	15	5.50	3.88	22	4.50	3.96	0.25	[−0.41; 0.91]	0.8%
Rousseaux 2021b	15	4.00	3.88	22	4.50	3.96	−0.12	[−0.78; 0.53]	0.8%
Sander Wint 2002	17	3.00	3.88	13	6.00	3.96	−0.75	[−1.50; 0.00]	0.7%
Sarig Bahat 2015	30	22.10	24.10	30	27.72	21.90	−0.24	[−0.75; 0.27]	0.8%
Sarig Bahat 2017	16	31.10	23.60	14	39.45	22.00	−0.36	[−1.08; 0.37]	0.7%
Schlechter 2020	58	4.00	2.22	57	4.00	4.44	0.00	[−0.37; 0.37]	0.9%
Semerci 2020	35	2.34	3.27	36	5.02	3.35	−0.80	[−1.29; −0.32]	0.8%
Sharifpour 2020	15	40.47	4.45	15	62.80	6.73	−3.81	[−5.07; −2.55]	0.5%
Shetty 2019	60	2.42	1.47	60	5.60	1.22	−2.34	[−2.81; −1.87]	0.9%
Smith 2019	25	60.68	21.10	25	49.76	28.00	0.43	[−0.13; 0.99]	0.8%
Spiegel 2019	61	4.70	3.88	59	5.10	3.96	−0.10	[−0.46; 0.26]	0.9%
Sweta 2019	25	1.28	0.89	25	2.60	1.38	−1.12	[−1.72; −0.52]	0.8%
Tanja−Dijkstra 2018a	20	2.05	2.63	28	2.63	2.32	−0.23	[−0.81; 0.34]	0.8%
Tanja−Dijkstra 2018b	22	0.63	0.93	28	2.63	2.32	−1.07	[−1.67; −0.47]	0.8%
Tennant 2020	61	10.05	16.43	29	14.76	18.48	−0.27	[−0.72; 0.17]	0.9%
Ustuner Top 2021	37	3.82	1.20	40	6.96	2.08	−1.81	[−2.35; −1.28]	0.8%
van Twillert 2007	19	3.00	1.70	19	5.60	1.73	−1.49	[−2.21; −0.76]	0.7%
Walker 2014	22	44.00	16.90	21	43.00	11.90	0.07	[−0.53; 0.67]	0.8%
Walther−Larsen 2019	28	27.00	18.52	31	15.00	18.52	0.64	[0.11; 1.16]	0.8%
Wong 2020	54	1.94	1.73	54	4.00	3.53	−0.74	[−1.13; −0.35]	0.9%
Wong 2021	21	5.50	2.40	19	5.60	1.40	−0.05	[−0.67; 0.57]	0.8%
Xiang 2021a	31	24.90	34.62	29	47.10	39.57	−0.59	[−1.11; −0.07]	0.8%
Xiang 2021b	30	37.00	38.83	29	47.10	39.57	−0.25	[−0.77; 0.26]	0.8%
Yang 2019	24	30.00	27.05	24	30.00	27.04	0.00	[−0.57; 0.57]	0.8%
Yilmaz Yelvar 2017	22	2.52	1.80	22	4.90	3.39	−0.86	[−1.48; −0.24]	0.8%
**Random effects model**	**4306**			**4251**			**−0.66**	**[−0.78; −0.55]**	**100.0%**

Heterogeneity: $I^2 = 85\%$, $\tau^2 = 0.3672$, $p < 0.01$

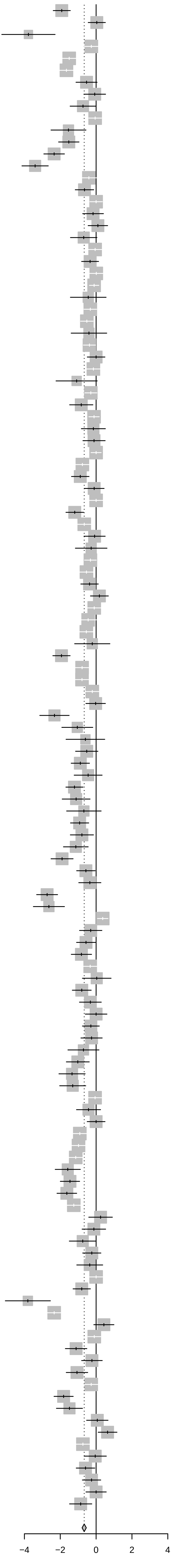

Appendix 6: Subgroup analyses
Appendix 6A: Subgroup analysis Study Design

Study	Experimental Total	Experimental Mean	Experimental SD	Control Total	Control Mean	Control SD	SMD	95%−CI	Weight
**Subgroup = Crossover**									
Alshatrat 2018	50	10.00	3.88	50	11.05	3.96	−0.27	[−0.66; 0.13]	0.8%
Asl Aminabadi 2012a	58	1.89	0.65	58	3.00	0.81	−1.50	[−1.92; −1.09]	0.8%
Asl Aminabadi 2012b	59	2.05	0.60	59	3.05	0.60	−1.66	[−2.08; −1.24]	0.8%
Asvanund 2015a	23	1.57	2.29	23	3.04	3.08	−0.53	[−1.12; 0.06]	0.7%
Asvanund 2015b	21	2.23	2.29	21	2.46	3.46	−0.08	[−0.68; 0.53]	0.7%
Atzori 2018a	15	2.00	1.20	15	3.60	3.00	−0.68	[−1.42; 0.06]	0.7%
Atzori 2018b	5	2.20	1.79	5	3.80	2.59	−0.65	[−1.94; 0.64]	0.4%
Austin 2020	16	2.20	1.60	16	3.40	1.60	−0.73	[−1.45; −0.01]	0.7%
Azurdia 2018	11	1.82	1.60	11	4.10	1.22	−1.54	[−2.52; −0.57]	0.5%
Bentsen 2001	23	31.57	24.69	23	35.74	23.55	−0.17	[−0.75; 0.41]	0.7%
Bentsen 2003	26	16.90	18.40	26	15.20	15.20	0.10	[−0.44; 0.64]	0.8%
Buldur 2021a	38	4.00	1.85	38	4.11	2.13	−0.05	[−0.50; 0.40]	0.8%
Buldur 2021b	36	3.52	1.65	34	4.11	1.83	−0.34	[−0.81; 0.14]	0.8%
Carrougher 2009	39	40.00	30.00	39	55.00	25.00	−0.54	[−0.99; −0.09]	0.8%
Chan 2007	8	38.13	34.00	8	53.75	33.38	−0.44	[−1.43; 0.56]	0.5%
Das2005	7	1.30	1.80	7	4.10	2.90	−1.09	[−2.24; 0.06]	0.5%
El−Sharkawi 2012	42	1.48	1.52	42	2.76	1.78	−0.77	[−1.21; −0.32]	0.8%
Frey 2018	27	5.60	2.00	27	7.15	1.70	−0.82	[−1.38; −0.27]	0.7%
Furman 2009a	38	1.58	1.40	38	3.66	2.00	−1.19	[−1.68; −0.70]	0.8%
Furman 2009b	38	2.39	1.80	38	3.66	2.00	−0.66	[−1.12; −0.20]	0.8%
Gray 2021a	43	3.11	2.35	43	3.37	2.58	−0.10	[−0.53; 0.32]	0.8%
Gray 2021b	39	2.53	2.37	39	3.62	2.49	−0.44	[−0.89; 0.01]	0.8%
Hoffman 2000	12	14.67	15.24	12	36.33	23.73	−1.05	[−1.91; −0.19]	0.6%
Hoffman 2001	7	5.00	38.83	7	30.00	39.57	−0.60	[−1.68; 0.48]	0.5%
Hoffman 2008	11	5.10	2.60	11	7.60	1.90	−1.06	[−1.96; −0.15]	0.6%
Jin 2016	33	36.90	18.60	33	53.10	16.10	−0.92	[−1.43; −0.41]	0.8%
Le May 2021	20	3.80	3.40	20	4.80	3.10	−0.30	[−0.92; 0.32]	0.7%
Maani 2011	12	4.50	1.95	12	6.25	2.49	−0.76	[−1.59; 0.08]	0.6%
McSherry 2018	12	5.80	2.90	12	5.70	2.60	0.04	[−0.77; 0.84]	0.6%
Mitrakul 2015a	21	0.86	1.49	21	1.62	2.94	−0.32	[−0.93; 0.29]	0.7%
Mitrakul 2015b	21	1.90	2.93	21	1.90	3.32	0.00	[−0.60; 0.60]	0.7%
Morris 2010	11	3.00	4.44	11	6.00	3.70	−0.71	[−1.57; 0.16]	0.6%
Niharika 2018a	18	2.56	1.65	18	5.22	2.18	−1.34	[−2.07; −0.61]	0.7%
Niharika 2018b	18	2.33	1.57	18	5.44	2.90	−1.31	[−2.03; −0.58]	0.7%
Schmitt 2011	54	40.00	30.86	54	55.00	29.39	−0.49	[−0.88; −0.11]	0.8%
Soltani 2018	39	52.90	27.80	39	64.40	28.30	−0.41	[−0.85; 0.04]	0.8%
van Twillert 2007	19	3.00	1.70	19	5.60	1.73	−1.49	[−2.21; −0.76]	0.7%
**Random effects model**	**970**			**968**			**−0.65**	**[−0.81; −0.48]**	**25.7%**
Heterogeneity: $I^2 = 66\%$ [52%; 76%], $p < 0.01$									
**Subgroup = Parallel**									
Akin 2021	50	4.68	1.44	50	6.74	0.45	−1.92	[−2.39; −1.44]	0.8%
Al−Halabi 2018	33	0.75	0.98	34	0.71	0.86	0.04	[−0.44; 0.52]	0.8%
Ali 2021	11	3.10	1.04	11	7.72	1.30	−3.78	[−5.26; −2.29]	0.3%
Aydin 2019	60	3.07	2.86	60	3.23	3.05	−0.05	[−0.41; 0.30]	0.8%
Babaie 2019	32	26.40	7.35	30	36.80	6.02	−1.52	[−2.09; −0.95]	0.7%
Bani Mohammad 2018	40	0.33	0.82	40	4.84	2.57	−2.34	[−2.92; −1.77]	0.7%
Barad 2020	36	2.50	1.75	36	8.00	1.43	−3.40	[−4.14; −2.67]	0.7%
Basak 2020	40	3.50	2.84	40	4.72	3.15	−0.40	[−0.85; 0.04]	0.8%
Basak 2021	30	4.93	3.07	31	6.67	2.17	−0.65	[−1.16; −0.13]	0.8%
Bekelis 2017	64	30.00	11.11	63	30.00	7.41	0.00	[−0.35; 0.35]	0.8%
Brown 2020	15	2.60	1.90	15	4.30	2.80	−0.69	[−1.43; 0.05]	0.7%
Butt 2021	55	3.58	3.10	55	3.55	3.30	0.01	[−0.36; 0.38]	0.8%
Canares 2021	19	4.13	3.88	20	5.70	3.96	−0.39	[−1.03; 0.24]	0.7%
Caruso 2020	106	0.72	1.32	114	0.89	1.68	−0.11	[−0.38; 0.15]	0.9%
Chan 2019a	64	3.08	3.29	59	4.20	3.59	−0.32	[−0.68; 0.03]	0.8%
Chan 2019b	63	2.41	3.11	66	4.21	3.76	−0.52	[−0.87; −0.17]	0.8%
Chang 2021	8	2.64	2.21	8	3.53	2.00	−0.40	[−1.39; 0.59]	0.5%
Chen 2020	68	3.35	2.38	68	4.35	2.95	−0.37	[−0.71; −0.03]	0.8%
Clerc 2021	35	0.00	2.70	29	0.00	5.40	0.00	[−0.49; 0.49]	0.8%
Darnall 2020	35	3.20	3.88	39	3.80	3.96	−0.15	[−0.61; 0.31]	0.8%
De Silva 2016	67	4.00	2.96	67	5.00	3.70	−0.30	[−0.64; 0.04]	0.8%
Deo 2020	20	3.77	2.66	20	6.00	2.62	−0.83	[−1.48; −0.18]	0.7%
Ding 2019	91	5.60	14.31	91	7.30	15.26	−0.11	[−0.41; 0.18]	0.9%
Dumoulin 2019	20	21.75	20.92	15	25.33	25.25	−0.15	[−0.82; 0.52]	0.7%
Ebrahimi 2017	20	5.35	2.68	20	5.60	1.35	−0.12	[−0.74; 0.50]	0.7%
Eijlers 2019	94	2.00	2.96	97	2.00	1.85	0.00	[−0.28; 0.28]	0.9%
Erdogan 2021	37	2.70	2.80	34	5.20	2.80	−0.88	[−1.37; −0.39]	0.8%
Felemban 2021	25	2.40	2.82	25	2.72	2.99	−0.11	[−0.66; 0.45]	0.7%
Fouks 2021	44	5.00	6.75	38	5.00	5.67	0.00	[−0.43; 0.43]	0.8%
Gershon 2004	22	13.80	20.20	22	15.40	17.00	−0.08	[−0.68; 0.51]	0.7%
Gold 2006	10	1.80	2.31	10	2.40	1.84	−0.28	[−1.16; 0.61]	0.6%
Gold 2018	70	1.31	1.59	73	1.93	2.22	−0.32	[−0.65; 0.01]	0.8%
Gold 2021	53	1.09	1.82	54	2.19	2.21	−0.54	[−0.93; −0.15]	0.8%
Goldman 2021a	35	2.00	5.40	31	4.00	5.40	−0.37	[−0.85; 0.12]	0.8%
Goldman 2021b	32	1.84	1.94	30	1.47	2.03	0.18	[−0.32; 0.68]	0.8%
Groninger 2021	52	3.80	2.40	36	5.10	2.30	−0.55	[−0.98; −0.11]	0.8%
Gulsen 2020	8	3.95	1.59	8	4.50	3.04	−0.21	[−1.20; 0.77]	0.5%
Guo 2014	49	2.63	1.27	49	7.64	3.41	−1.93	[−2.41; −1.45]	0.8%
Gür 2020a	54	4.98	1.69	54	6.38	1.86	−0.78	[−1.17; −0.39]	0.8%
Gür 2020b	55	4.96	1.72	54	6.38	1.86	−0.79	[−1.18; −0.40]	0.8%
Gür 2020c	55	5.96	2.05	54	6.38	1.86	−0.21	[−0.59; 0.16]	0.8%
Haisley 2020	26	3.80	3.88	26	3.90	3.96	−0.03	[−0.57; 0.52]	0.8%
Hassannia 2021	20	1.10	0.30	20	2.30	0.65	−2.32	[−3.14; −1.50]	0.6%
Hoxhallari 2019	21	0.14	0.47	20	0.60	1.14	−0.52	[−1.15; 0.10]	0.7%
Hua 2015	33	2.42	1.85	32	4.19	2.12	−0.88	[−1.39; −0.37]	0.8%
Hwang 2014	13	5.08	2.02	13	6.15	2.60	−0.45	[−1.22; 0.33]	0.6%
Inangil 2020	40	1.30	2.15	40	4.95	3.65	−1.21	[−1.69; −0.73]	0.8%
JahaniShoorab 2015	15	16.70	16.50	15	39.30	22.50	−1.11	[−1.89; −0.34]	0.6%
Jeffs 2014	8	28.70	15.70	10	38.40	11.60	−0.68	[−1.65; 0.28]	0.5%
Jin 2018	20	3.87	0.55	20	4.42	0.79	−0.79	[−1.44; −0.15]	0.7%
Joo 2021	19	3.70	1.40	19	5.50	1.70	−1.13	[−1.82; −0.44]	0.7%
Karaman 2021	30	2.86	1.22	30	6.33	2.24	−1.90	[−2.52; −1.28]	0.7%
Karaveli Çakir 2021	30	2.76	1.25	30	3.76	2.11	−0.57	[−1.09; −0.05]	0.8%
Kipping 2012	20	3.90	3.88	21	5.30	3.96	−0.35	[−0.97; 0.27]	0.7%
Koc Özkan 2019	46	1.97	1.20	43	6.81	2.20	−2.73	[−3.32; −2.15]	0.7%
Konstantatos 2009	43	7.30	3.88	43	5.30	3.96	0.51	[0.08; 0.94]	0.8%
Koushali 2017	20	5.50	1.30	20	8.40	0.80	−2.63	[−3.50; −1.77]	0.6%
Laghlam 2021	90	5.00	5.40	90	3.00	5.40	0.37	[0.07; 0.66]	0.9%
Litwin 2021	31	2.60	2.10	27	3.80	2.10	−0.56	[−1.09; −0.04]	0.8%
Liu 2020	30	0.80	1.06	23	2.26	2.38	−0.82	[−1.39; −0.25]	0.7%
Luczak 2021	52	4.30	2.20	51	5.10	2.60	−0.33	[−0.72; 0.06]	0.8%
Melcer 2021	30	2.50	1.50	30	3.80	1.70	−0.80	[−1.33; −0.27]	0.8%
Mohanasundari 2021	35	3.00	1.35	35	5.00	9.45	−0.29	[−0.76; 0.18]	0.8%
Momenyan 2021	26	9.01	0.78	26	9.37	0.57	−0.52	[−1.07; 0.03]	0.7%
Morales Tejera 2020	22	2.67	1.91	22	3.11	1.47	−0.25	[−0.85; 0.34]	0.7%
Mukherjee 2020	22	1.50	0.80	21	2.71	1.45	−1.02	[−1.66; −0.38]	0.7%
Nunna 2019	35	2.94	1.76	35	3.06	2.31	−0.06	[−0.53; 0.41]	0.8%
Nusser 2021	17	2.20	1.20	18	3.20	3.00	−0.42	[−1.09; 0.25]	0.7%
Osmanlliu 2020	31	3.00	6.75	31	3.00	6.08	0.00	[−0.50; 0.50]	0.8%
Ozalp Gerceker 2018	40	1.50	3.88	40	5.10	3.96	−0.91	[−1.37; −0.45]	0.8%
Ozalp Gerceker 2020a	45	1.20	2.20	46	4.10	3.50	−0.98	[−1.42; −0.55]	0.8%
Ozalp Gerceker 2020b	45	1.00	1.50	46	4.10	3.50	−1.14	[−1.58; −0.69]	0.8%
Ozalp Gerceker 2021	21	2.40	1.80	21	5.30	1.80	−1.58	[−2.28; −0.88]	0.7%
Powers 2021a	34	3.03	2.30	34	6.37	2.23	−1.46	[−2.00; −0.92]	0.8%
Powers 2021b	35	2.77	2.13	34	6.37	2.23	−1.63	[−2.18; −1.08]	0.7%
Ran 2021	60	1.58	1.08	60	2.86	0.96	−1.24	[−1.64; −0.85]	0.8%
Rousseaux 2021a	15	5.50	3.88	22	4.50	3.96	0.25	[−0.41; 0.91]	0.7%
Rousseaux 2021b	15	4.00	3.88	22	4.50	3.96	−0.12	[−0.78; 0.53]	0.7%
Sander Wint 2002	17	3.00	3.88	13	6.00	3.96	−0.75	[−1.50; 0.00]	0.6%
Sarig Bahat 2015	30	22.10	24.10	30	27.72	21.90	−0.24	[−0.75; 0.27]	0.8%
Sarig Bahat 2017	16	31.10	23.60	14	39.45	22.00	−0.36	[−1.08; 0.37]	0.7%
Schlechter 2020	58	4.00	2.22	57	4.00	4.44	0.00	[−0.37; 0.37]	0.8%
Semerci 2020	35	2.34	3.27	36	5.02	3.35	−0.80	[−1.29; −0.32]	0.8%
Sharifpour 2020	15	40.47	4.45	15	62.80	6.73	−3.81	[−5.07; −2.55]	0.4%
Shetty 2019	60	2.42	1.47	60	5.60	1.22	−2.34	[−2.81; −1.87]	0.8%
Smith 2019	25	60.68	21.10	25	49.76	28.00	0.43	[−0.13; 0.99]	0.7%
Spiegel 2019	61	4.70	3.88	59	5.10	3.96	−0.10	[−0.46; 0.26]	0.8%
Sweta 2019	25	1.28	0.89	25	2.60	1.38	−1.12	[−1.72; −0.52]	0.7%
Tanja−Dijkstra 2018a	20	2.05	2.63	28	2.63	2.32	−0.23	[−0.81; 0.34]	0.7%
Tanja−Dijkstra 2018b	22	0.63	0.93	28	2.63	2.32	−1.07	[−1.67; −0.47]	0.7%
Tennant 2020	61	10.05	16.43	29	14.76	18.48	−0.27	[−0.72; 0.17]	0.8%
Ustuner Top 2021	37	3.82	1.20	40	6.96	2.08	−1.81	[−2.35; −1.28]	0.8%
Walker 2014	22	44.00	16.90	21	43.00	11.90	0.07	[−0.53; 0.67]	0.7%
Walther−Larsen 2019	28	27.00	18.52	31	15.00	18.52	0.64	[0.11; 1.16]	0.8%
Wong 2020	54	1.94	1.73	54	4.00	3.53	−0.74	[−1.13; −0.35]	0.8%
Wong 2021	21	5.50	2.40	19	5.60	1.40	−0.05	[−0.67; 0.57]	0.7%
Xiang 2021a	31	24.90	34.62	29	47.10	39.57	−0.59	[−1.11; −0.07]	0.8%
Xiang 2021b	30	37.00	38.83	29	47.10	39.57	−0.25	[−0.77; 0.26]	0.8%
Yang 2019	24	30.00	27.05	24	30.00	27.04	0.00	[−0.57; 0.57]	0.7%
Yilmaz Yelvar 2017	22	2.52	1.80	22	4.90	3.39	−0.86	[−1.48; −0.24]	0.7%
**Random effects model**	**3626**			**3574**			**−0.65**	**[−0.79; −0.52]**	**74.3%**
Heterogeneity: $I^2 = 87\%$ [84%; 89%], $p < 0.01$									
**Random effects model**	**4596**			**4542**			**−0.65**	**[−0.76; −0.55]**	**100.0%**
Heterogeneity: $I^2 = 84\%$ [82%; 86%], $p < 0.01$									
Between subgroups p = 0.95									

Standardised Mean Difference

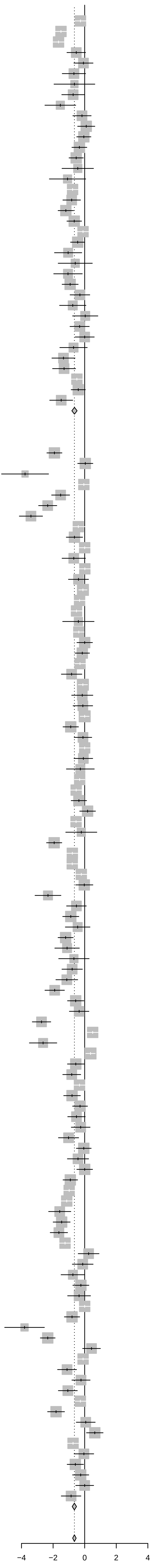

Appendix 6B: Subgroup analysis Type of pain

Study	Experimental Total	Experimental Mean	Experimental SD	Control Total	Control Mean	Control SD	SMD	95%−CI	Weight
**Subgroup = Acute**									
Akin 2021	50	4.68	1.44	50	6.74	0.45	−1.92	[−2.39; −1.44]	0.8%
Bekelis 2017	64	30.00	11.11	63	30.00	7.41	0.00	[−0.35; 0.35]	0.8%
Butt 2021	55	3.58	3.10	55	3.55	3.30	0.01	[−0.36; 0.38]	0.8%
Clerc 2021	35	0.00	2.70	29	0.00	5.40	0.00	[−0.49; 0.49]	0.8%
Eijlers 2019	94	2.00	2.96	97	2.00	1.85	0.00	[−0.28; 0.28]	0.9%
Frey 2018	27	5.60	2.00	27	7.15	1.70	−0.82	[−1.38; −0.27]	0.7%
Groninger 2021	52	3.80	2.40	36	5.10	2.30	−0.55	[−0.98; −0.11]	0.8%
Gür 2020a	54	4.98	1.69	54	6.38	1.86	−0.78	[−1.17; −0.39]	0.8%
Gür 2020b	55	4.96	1.72	54	6.38	1.86	−0.79	[−1.18; −0.40]	0.8%
Gür 2020c	55	5.96	2.05	54	6.38	1.86	−0.21	[−0.59; 0.16]	0.8%
Haisley 2020	26	3.80	3.88	26	3.90	3.96	−0.03	[−0.57; 0.52]	0.8%
Hassannia 2021	20	1.10	0.30	20	2.30	0.65	−2.32	[−3.14; −1.50]	0.6%
Hoxhallari 2019	21	0.14	0.47	20	0.60	1.14	−0.52	[−1.15; 0.10]	0.7%
Jin 2018	20	3.87	0.55	20	4.42	0.79	−0.79	[−1.44; −0.15]	0.7%
Momenyan 2021	26	9.01	0.78	26	9.37	0.57	−0.52	[−1.07; 0.03]	0.7%
Powers 2021a	34	3.03	2.30	34	6.37	2.23	−1.46	[−2.00; −0.92]	0.8%
Powers 2021b	35	2.77	2.13	34	6.37	2.23	−1.63	[−2.18; −1.08]	0.7%
Rousseaux 2021a	15	5.50	3.88	22	4.50	3.96	0.25	[−0.41; 0.91]	0.7%
Rousseaux 2021b	15	4.00	3.88	22	4.50	3.96	−0.12	[−0.78; 0.53]	0.7%
Spiegel 2019	61	4.70	3.88	59	5.10	3.96	−0.10	[−0.46; 0.26]	0.8%
Tennant 2020	61	10.05	16.43	29	14.76	18.48	−0.27	[−0.72; 0.17]	0.8%
Wong 2021	21	5.50	2.40	19	5.60	1.40	−0.05	[−0.67; 0.57]	0.7%
Yang 2019	24	30.00	27.05	24	30.00	27.04	0.00	[−0.57; 0.57]	0.7%
**Random effects model**	**920**			**874**			**−0.53**	**[−0.78; −0.28]**	**17.6%**
Heterogeneity: I^2 = 84% [77%; 89%], p < 0.01									
**Subgroup = Chronic**									
Austin 2020	16	2.20	1.60	16	3.40	1.60	−0.73	[−1.45; −0.01]	0.7%
Azurdia 2018	11	1.82	1.60	11	4.10	1.22	−1.54	[−2.52; −0.57]	0.5%
Bani Mohammad 2018	40	0.33	0.82	40	4.84	2.57	−2.34	[−2.92; −1.77]	0.7%
Darnall 2020	35	3.20	3.88	39	3.80	3.96	−0.15	[−0.61; 0.31]	0.8%
Gulsen 2020	8	3.95	1.59	8	4.50	3.04	−0.21	[−1.20; 0.77]	0.5%
Hwang 2014	13	5.08	2.02	13	6.15	2.60	−0.45	[−1.22; 0.33]	0.6%
Jin 2016	33	36.90	18.60	33	53.10	16.10	−0.92	[−1.43; −0.41]	0.8%
Morales Tejera 2020	22	2.67	1.91	22	3.11	1.47	−0.25	[−0.85; 0.34]	0.7%
Mukherjee 2020	22	1.50	0.80	21	2.71	1.45	−1.02	[−1.66; −0.38]	0.7%
Nusser 2021	17	2.20	1.20	18	3.20	3.00	−0.42	[−1.09; 0.25]	0.7%
Sarig Bahat 2015	30	22.10	24.10	30	27.72	21.90	−0.24	[−0.75; 0.27]	0.8%
Sarig Bahat 2017	16	31.10	23.60	14	39.45	22.00	−0.36	[−1.08; 0.37]	0.7%
Sharifpour 2020	15	40.47	4.45	15	62.80	6.73	−3.81	[−5.07; −2.55]	0.4%
Yilmaz Yelvar 2017	22	2.52	1.80	22	4.90	3.39	−0.86	[−1.48; −0.24]	0.7%
**Random effects model**	**300**			**302**			**−0.88**	**[−1.30; −0.46]**	**9.3%**
Heterogeneity: I^2 = 82% [71%; 89%], p < 0.01									
**Subgroup = Procedural**									
Al−Halabi 2018	33	0.75	0.98	34	0.71	0.86	0.04	[−0.44; 0.52]	0.8%
Ali 2021	11	3.10	1.04	11	7.72	1.30	−3.78	[−5.26; −2.29]	0.3%
Alshatrat 2018	50	10.00	3.88	50	11.05	3.96	−0.27	[−0.66; 0.13]	0.8%
Asl Aminabadi 2012a	58	1.89	0.65	58	3.00	0.81	−1.50	[−1.92; −1.09]	0.8%
Asl Aminabadi 2012b	59	2.05	0.60	59	3.05	0.60	−1.66	[−2.08; −1.24]	0.8%
Asvanund 2015a	23	1.57	2.29	23	3.04	3.08	−0.53	[−1.12; 0.06]	0.7%
Asvanund 2015b	21	2.23	2.29	21	2.46	3.46	−0.08	[−0.68; 0.53]	0.7%
Atzori 2018a	15	2.00	1.20	15	3.60	3.00	−0.68	[−1.42; 0.06]	0.7%
Atzori 2018b	5	2.20	1.79	5	3.80	2.59	−0.65	[−1.94; 0.64]	0.4%
Aydin 2019	60	3.07	2.86	60	3.23	3.05	−0.05	[−0.41; 0.30]	0.8%
Babaie 2019	32	26.40	7.35	30	36.80	6.02	−1.52	[−2.09; −0.95]	0.7%
Barad 2020	36	2.50	1.75	36	8.00	1.43	−3.40	[−4.14; −2.67]	0.7%
Basak 2020	40	3.50	2.84	40	4.72	3.15	−0.40	[−0.85; 0.04]	0.8%
Basak 2021	30	4.93	3.07	31	6.67	2.17	−0.65	[−1.16; −0.13]	0.8%
Bentsen 2001	23	31.57	24.69	23	35.74	23.55	−0.17	[−0.75; 0.41]	0.7%
Bentsen 2003	26	16.90	18.40	26	15.20	15.20	0.10	[−0.44; 0.64]	0.8%
Brown 2020	15	2.60	1.90	15	4.30	2.80	−0.69	[−1.43; 0.05]	0.7%
Buldur 2021a	38	4.00	1.85	38	4.11	2.13	−0.05	[−0.50; 0.40]	0.8%
Buldur 2021b	36	3.52	1.65	34	4.11	1.83	−0.34	[−0.81; 0.14]	0.8%
Canares 2021	19	4.13	3.88	20	5.70	3.96	−0.39	[−1.03; 0.24]	0.7%
Carrougher 2009	39	40.00	30.00	39	55.00	25.00	−0.54	[−0.99; −0.09]	0.8%
Caruso 2020	106	0.72	1.32	114	0.89	1.68	−0.11	[−0.38; 0.15]	0.9%
Chan 2007	8	38.13	34.00	8	53.75	33.38	−0.44	[−1.43; 0.56]	0.5%
Chan 2019a	64	3.08	3.29	59	4.20	3.59	−0.32	[−0.68; 0.03]	0.8%
Chan 2019b	63	2.41	3.11	66	4.21	3.76	−0.52	[−0.87; −0.17]	0.8%
Chang 2021	8	2.64	2.21	8	3.53	2.00	−0.40	[−1.39; 0.59]	0.5%
Chen 2020	68	3.35	2.38	68	4.35	2.95	−0.37	[−0.71; −0.03]	0.8%
Das2005	7	1.30	1.80	7	4.10	2.90	−1.09	[−2.24; 0.06]	0.5%
De Silva 2016	67	4.00	2.96	67	5.00	3.70	−0.30	[−0.64; 0.04]	0.8%
Deo 2020	20	3.77	2.66	20	6.00	2.62	−0.83	[−1.48; −0.18]	0.7%
Ding 2019	91	5.60	14.31	91	7.30	15.26	−0.11	[−0.41; 0.18]	0.9%
Dumoulin 2019	20	21.75	20.92	15	25.33	25.25	−0.15	[−0.82; 0.52]	0.7%
Ebrahimi 2017	20	5.35	2.68	20	5.60	1.35	−0.12	[−0.74; 0.50]	0.7%
El−Sharkawi 2012	42	1.48	1.52	42	2.76	1.78	−0.77	[−1.21; −0.32]	0.8%
Erdogan 2021	37	2.70	2.80	34	5.20	2.80	−0.88	[−1.37; −0.39]	0.8%
Felemban 2021	25	2.40	2.82	25	2.72	2.99	−0.11	[−0.66; 0.45]	0.7%
Fouks 2021	44	5.00	6.75	38	5.00	5.67	0.00	[−0.43; 0.43]	0.8%
Furman 2009a	38	1.58	1.40	38	3.66	2.00	−1.19	[−1.68; −0.70]	0.8%
Furman 2009b	38	2.39	1.80	38	3.66	2.00	−0.66	[−1.12; −0.20]	0.8%
Gershon 2004	22	13.80	20.20	22	15.40	17.00	−0.08	[−0.68; 0.51]	0.7%
Gold 2006	10	1.80	2.31	10	2.40	1.84	−0.28	[−1.16; 0.61]	0.6%
Gold 2018	70	1.31	1.59	73	1.93	2.22	−0.32	[−0.65; 0.01]	0.8%
Gold 2021	53	1.09	1.82	54	2.19	2.21	−0.54	[−0.93; −0.15]	0.8%
Goldman 2021a	35	2.00	5.40	31	4.00	5.40	−0.37	[−0.85; 0.12]	0.8%
Goldman 2021b	32	1.84	1.94	30	1.47	2.03	0.18	[−0.32; 0.68]	0.8%
Gray 2021a	43	3.11	2.35	43	3.37	2.58	−0.10	[−0.53; 0.32]	0.8%
Gray 2021b	39	2.53	2.37	39	3.62	2.49	−0.44	[−0.89; 0.01]	0.8%
Guo 2014	49	2.63	1.27	49	7.64	3.41	−1.93	[−2.41; −1.45]	0.8%
Hoffman 2000	12	14.67	15.24	12	36.33	23.73	−1.05	[−1.91; −0.19]	0.6%
Hoffman 2001	7	5.00	38.83	7	30.00	39.57	−0.60	[−1.68; 0.48]	0.5%
Hoffman 2008	11	5.10	2.60	11	7.60	1.90	−1.06	[−1.96; −0.15]	0.6%
Hua 2015	33	2.42	1.85	32	4.19	2.12	−0.88	[−1.39; −0.37]	0.8%
Inangil 2020	40	1.30	2.15	40	4.95	3.65	−1.21	[−1.69; −0.73]	0.8%
JahaniShoorab 2015	15	16.70	16.50	15	39.30	22.50	−1.11	[−1.89; −0.34]	0.6%
Jeffs 2014	8	28.70	15.70	10	38.40	11.60	−0.68	[−1.65; 0.28]	0.5%
Joo 2021	19	3.70	1.40	19	5.50	1.70	−1.13	[−1.82; −0.44]	0.7%
Karaman 2021	30	2.86	1.22	30	6.33	2.24	−1.90	[−2.52; −1.28]	0.7%
Karaveli Çakir 2021	30	2.76	1.25	30	3.76	2.11	−0.57	[−1.09; −0.05]	0.8%
Kipping 2012	20	3.90	3.88	21	5.30	3.96	−0.35	[−0.97; 0.27]	0.7%
Koc Özkan 2019	46	1.97	1.20	43	6.81	2.20	−2.73	[−3.32; −2.15]	0.7%
Konstantatos 2009	43	7.30	3.88	43	5.30	3.96	0.51	[0.08; 0.94]	0.8%
Koushali 2017	20	5.50	1.30	20	8.40	0.80	−2.63	[−3.50; −1.77]	0.6%
Laghlam 2021	90	5.00	5.40	90	3.00	5.40	0.37	[0.07; 0.66]	0.9%
Le May 2021	20	3.80	3.40	20	4.80	3.10	−0.30	[−0.92; 0.32]	0.7%
Litwin 2021	31	2.60	2.10	27	3.80	2.10	−0.56	[−1.09; −0.04]	0.8%
Liu 2020	30	0.80	1.06	23	2.26	2.38	−0.82	[−1.39; −0.25]	0.7%
Luczak 2021	52	4.30	2.20	51	5.10	2.60	−0.33	[−0.72; 0.06]	0.8%
Maani 2011	12	4.50	1.95	12	6.25	2.49	−0.76	[−1.59; 0.08]	0.6%
McSherry 2018	12	5.80	2.90	12	5.70	2.60	0.04	[−0.77; 0.84]	0.6%
Melcer 2021	30	2.50	1.50	30	3.80	1.70	−0.80	[−1.33; −0.27]	0.8%
Mitrakul 2015a	21	0.86	1.49	21	1.62	2.94	−0.32	[−0.93; 0.29]	0.7%
Mitrakul 2015b	21	1.90	2.93	21	1.90	3.32	0.00	[−0.60; 0.60]	0.7%
Mohanasundari 2021	35	3.00	1.35	35	5.00	9.45	−0.29	[−0.76; 0.18]	0.8%
Morris 2010	11	3.00	4.44	11	6.00	3.70	−0.71	[−1.57; 0.16]	0.6%
Niharika 2018a	18	2.56	1.65	18	5.22	2.18	−1.34	[−2.07; −0.61]	0.7%
Niharika 2018b	18	2.33	1.57	18	5.44	2.90	−1.31	[−2.03; −0.58]	0.7%
Nunna 2019	35	2.94	1.76	35	3.06	2.31	−0.06	[−0.53; 0.41]	0.8%
Osmanlliu 2020	31	3.00	6.75	31	3.00	6.08	0.00	[−0.50; 0.50]	0.8%
Ozalp Gerceker 2018	40	1.50	3.88	40	5.10	3.96	−0.91	[−1.37; −0.45]	0.8%
Ozalp Gerceker 2020a	45	1.20	2.20	46	4.10	3.50	−0.98	[−1.42; −0.55]	0.8%
Ozalp Gerceker 2020b	45	1.00	1.50	46	4.10	3.50	−1.14	[−1.58; −0.69]	0.8%
Ozalp Gerceker 2021	21	2.40	1.80	21	5.30	1.80	−1.58	[−2.28; −0.88]	0.7%
Ran 2021	60	1.58	1.08	60	2.86	0.96	−1.24	[−1.64; −0.85]	0.8%
Sander Wint 2002	17	3.00	3.88	13	6.00	3.96	−0.75	[−1.50; 0.00]	0.6%
Schlechter 2020	58	4.00	2.22	57	4.00	4.44	0.00	[−0.37; 0.37]	0.8%
Schmitt 2011	54	40.00	30.86	54	55.00	29.39	−0.49	[−0.88; −0.11]	0.8%
Semerci 2020	35	2.34	3.27	36	5.02	3.35	−0.80	[−1.29; −0.32]	0.8%
Shetty 2019	60	2.42	1.47	60	5.60	1.22	−2.34	[−2.81; −1.87]	0.8%
Smith 2019	25	60.68	21.10	25	49.76	28.00	0.43	[−0.13; 0.99]	0.7%
Soltani 2018	39	52.90	27.80	39	64.40	28.30	−0.41	[−0.85; 0.04]	0.8%
Sweta 2019	25	1.28	0.89	25	2.60	1.38	−1.12	[−1.72; −0.52]	0.7%
Tanja−Dijkstra 2018a	20	2.05	2.63	28	2.63	2.32	−0.23	[−0.81; 0.34]	0.7%
Tanja−Dijkstra 2018b	22	0.63	0.93	28	2.63	2.32	−1.07	[−1.67; −0.47]	0.7%
Ustuner Top 2021	37	3.82	1.20	40	6.96	2.08	−1.81	[−2.35; −1.28]	0.8%
van Twillert 2007	19	3.00	1.70	19	5.60	1.73	−1.49	[−2.21; −0.76]	0.7%
Walker 2014	22	44.00	16.90	21	43.00	11.90	0.07	[−0.53; 0.67]	0.7%
Walther−Larsen 2019	28	27.00	18.52	31	15.00	18.52	0.64	[0.11; 1.16]	0.8%
Wong 2020	54	1.94	1.73	54	4.00	3.53	−0.74	[−1.13; −0.35]	0.8%
Xiang 2021a	31	24.90	34.62	29	47.10	39.57	−0.59	[−1.11; −0.07]	0.8%
Xiang 2021b	30	37.00	38.83	29	47.10	39.57	−0.25	[−0.77; 0.26]	0.8%
**Random effects model**	**3376**			**3366**			**−0.65**	**[−0.78; −0.52]**	**73.1%**
Heterogeneity: I^2 = 84% [82%; 87%], p < 0.01									
**Random effects model**	**4596**			**4542**			**−0.64**	**[−0.75; −0.53**	**100.0%**
Heterogeneity: I^2 = 84% [82%; 86%], p < 0.01									
Between subgroups p = 0.35									

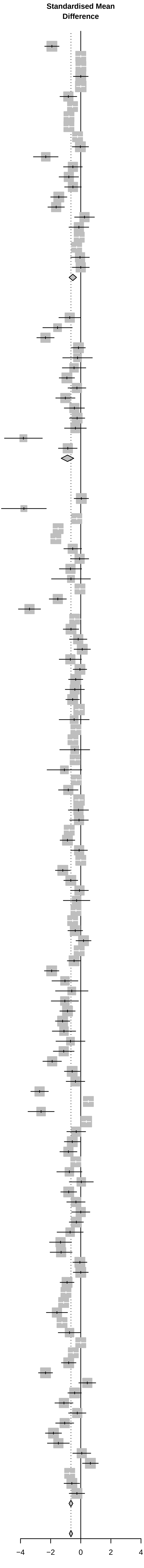

Appendix 7: Meta-regression analyses

Results of univariate meta-regression analyses

Variable		Estimate	se	p-value
Year of publication	Intercept 2000-2010	-0.6688	0.1705	<.0001
p = 0.99	Subgroup 2011-2015	-0.0335	0.2351	0.8867
I2 = 84.2%, R2=0.00%	Subgroup 2016-2020	0.0235	0.1895	0.9013
	Subgroup 2021	0.0364	0.1998	0.8555
Number of participants	Intercept 0-50	-0.7217	0.0931	<.0001
p = 0.25	Subgroup 51-100	0.2044	0.1355	0.1314
I2 = 84.3%, R2=0.00%	Subgroup >100	0.0075	0.1406	0.9575
**Type of controlgroup**	Intercept Standard of Care /No distraction	-0.7580	0.0644	<.0001
**p < 0.01**	Subgroup Distraction + SoC	0.5147	0.1476	0.0005
I2 = 83.2%, R2=5.48%	Subgroup Standard Therapy	0.2176	0.1954	0.2654
Gender *(% of male participants)*	Intercept 0-39%	-0.6253	0.1227	<.0001
p = 0.68	Subgroup 40-60%	-0.0689	0.1449	0.6345
I2 = 84.3%, R2=0.00%	Subgroup 61-100%	0.0491	0.1693	0.7720
**Mean age**	Intercept	-0.8328	0.0975	<.0001
**p = 0.02**	Age	0.0067	0.0029	0.0223
I2 = 83.7%, R2=3.10%				
Age range of participants	Intercept Children (0-12)	-0.7614	0.0915	<.0001
p = 0.28	Subgroup Adolescents (12-21)	0.1106	0.2003	0.5806
I2 = 84.0%, R2=0.35%	Subgroup Adults (22-100)	0.1922	0.1206	0.1109
Type of patients	Intercept Inpatients	-0.5521	-0.0805	<.0001
p = 0.10	Subgroup Outpatients	-0.1875	0.1115	0.0926
I2 = 83.5%, R2=4.00%				
Diagnosis	Intercept Other	-0.5815	0.0879	<.0001
p = 0.76	Subgroup Dental care	-0.0955	0.1582	-.5463
I2 = 84.1%, R2=0.00%	Subgroup Vascular acces	-0.1211	0.1457	0.4060
	Subgroup Wound care	-0.1441	0.1673	0.3892
**Continent**	Intercept Asia	-0.9628	0.0764	<.0001
**p < 0.01**	Subgroup Europe	0.6768	0.1675	<.0001
I2 = 80.6%, R2=20.84%	Subgroup North-America	0.5246	0.1158	<.0001
	Subgroup Oceania	0.7145	0.2058	0.0005
**World bank income class**	Intercept Low/Lower Middle	-0.9952	0.1630	<.0001
**p < 0.01**	Subgroup Upper middle	0.0041	0.1856	0.0220
I2 = 80.4%, R2=22.52%	Subgroup High	0.6141	0.1766	0.0005
Co-analgesia	Intercept No analgesia	-0.6334	0.0994	<.0001
p = 0.23	Subgroup Non opioids	0.0789	0.1388	0.5699
I2 = 84.2%, R2=0.00%	Subgroup Opioids	0.0108	0.1769	0.9511
	Subgroup Unknown	-0.2594	0.1671	0.1206
**VR software**	Intercept 2D video	-0.8773	0.1348	<.0001
**p = 0.04**	Subgroup 3D video	0.0499	0.1852	0.7877
I2 = 83.17%, R2=5.76%	Subgroup 3D VR environment	0.1951	0.1784	0.2741
	Subgroup 3D VR game	0.4050	0.1753	0.0615
	Subgroup VR Therapy	0.4339	0.1886	0.0642
**Interactiveness**	Intercept No	-0.7715	0.0732	<.0001
**p = 0.01**	Subgroup Yes	0.2873	0.1124	0.0106
I2 = 83.5%, R2=4.49%				
Frequency of VR sessions	Intercept More than one session	-0.5533	0.1569	0.0004
p = 0.50	Subgroup One session	-0.1124	0.1683	0.5042
I2 = 84.2%, R2=0.00%				
Duration of VR sessions	Intercept Unknown	-0.5792	0.1407	<.0001
p = 0.87	Subgroup <5 minutes	0.0091	0.2225	0.9675
I2 = 84.5%, R2=0.00%	Subgroup 5-15 minutes	-0.0891	0.1658	0.5910
	Subgroup >15 minutes	-0.1199	0.1759	0.4956
Total time in VR	Intercept Unknown	-0.5971	0.1481	<.0001
p = 0.90	Subgroup <5 minutes	0.0472	0.2196	0.8299
I2 = 84.5%, R2=0.00%	Subgroup 5-15 minutes	-0.0939	0.1772	0.5962
	Subgroup 15-30 minutes	-0.0162	0.1908	0.9324
	Subgroup 31-60 minutes	-0.2361	0.2475	0.3402
	Subgroup >60 minutes	-0.0714	0.2752	0.7953
**Pain score of controlgroup**	intrcpt <4	-0.4395	0.0830	<.0001
**p < 0.01**	Subgroup >4	-0.3759	0.1110	0.0007
I2 = 83.2%, R2=6.28%				

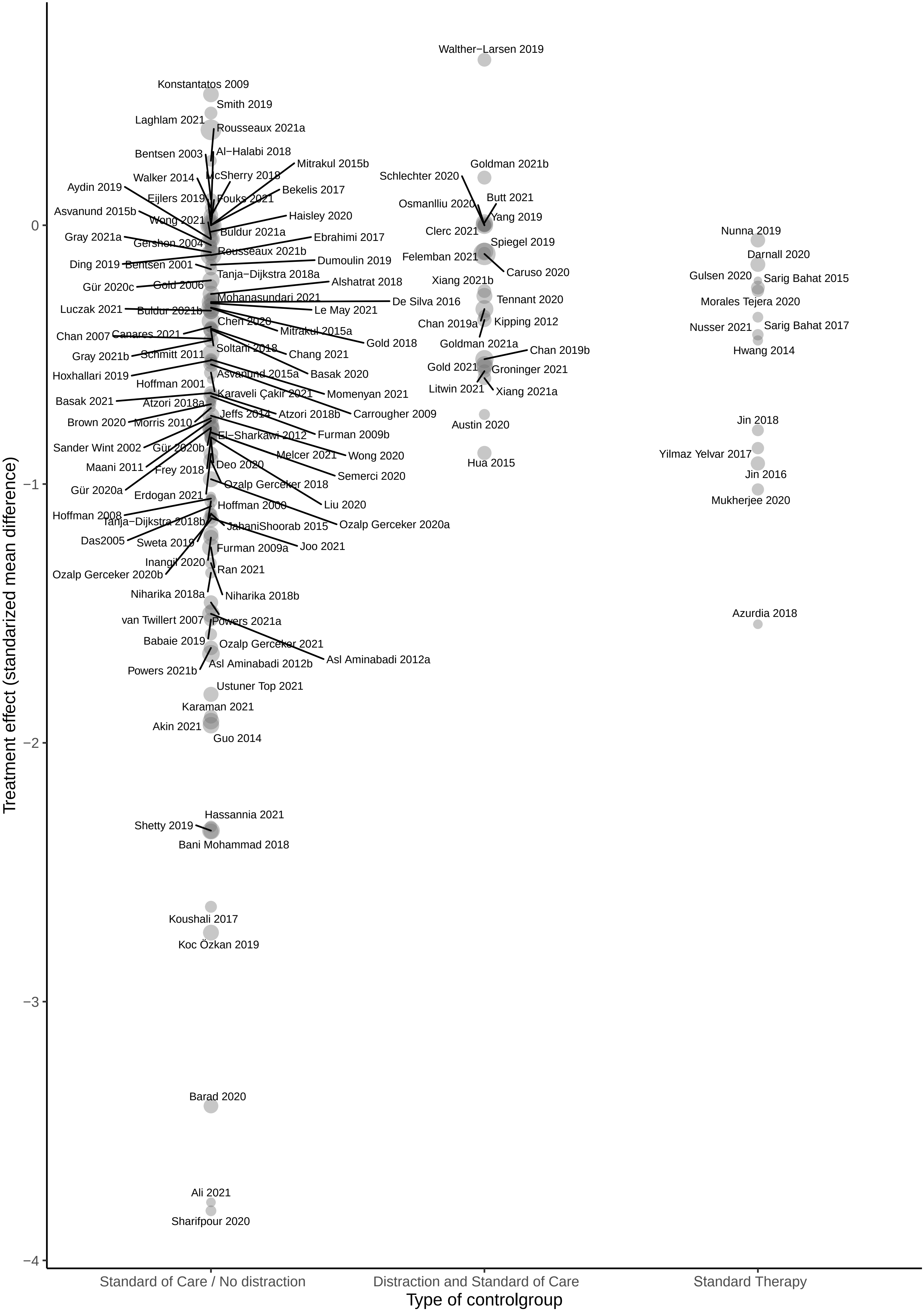
Walther−Larsen 2019
Konstantatos 2009
Smith 2019
Laghlam 2021
Rousseaux 2021a
Bentsen 2003
Al−Halabi 2018
Mitrakul 2015b
Walker 2014
McSherry 2018
Aydin 2019
Bekelis 2017
Eijlers 2019
Fouks 2021
Asvanund 2015b
Haisley 2020
Wong 2021
Buldur 2021a
Gray 2021a
Ebrahimi 2017
Gershon 2004
Rousseaux 2021b
Ding 2019
Bentsen 2001
Dumoulin 2019
Tanja−Dijkstra 2018a
Alshatrat 2018
Gür 2020c
Gold 2006
Mohanasundari 2021
De Silva 2016
Luczak 2021
Buldur 2021b
Le May 2021
Chen 2020
Mitrakul 2015a
Chan 2007
Canares 2021
Gold 2018
Soltani 2018
Gray 2021b
Schmitt 2011
Chang 2021
Hoxhallari 2019
Asvanund 2015a
Basak 2020
Hoffman 2001
Karaveli Çakir 2021
Momenyan 2021
Basak 2021
Atzori 2018a
Jeffs 2014
Atzori 2018b
Carrougher 2009
Brown 2020
Morris 2010
El−Sharkawi 2012
Furman 2009b
Sander Wint 2002
Gür 2020b
Melcer 2021
Wong 2020
Maani 2011
Deo 2020
Frey 2018
Semerci 2020
Gür 2020a
Ozalp Gerceker 2018
Erdogan 2021
Liu 2020
Hoffman 2000
Hoffman 2008
Tanja−Dijkstra 2018b
Ozalp Gerceker 2020a
JahaniShoorab 2015
Das2005
Sweta 2019
Joo 2021
Furman 2009a
Inangil 2020
Ran 2021
Ozalp Gerceker 2020b
Niharika 2018a
Niharika 2018b
van Twillert 2007
Powers 2021a
Babaie 2019
Ozalp Gerceker 2021
Asl Aminabadi 2012a
Asl Aminabadi 2012b
Powers 2021b
Ustuner Top 2021
Karaman 2021
Akin 2021
Guo 2014
Hassannia 2021
Shetty 2019
Bani Mohammad 2018
Koushali 2017
Koc Özkan 2019
Barad 2020
Ali 2021
Sharifpour 2020
Goldman 2021b
Schlechter 2020
Butt 2021
Osmanlliu 2020
Yang 2019
Clerc 2021
Spiegel 2019
Felemban 2021
Caruso 2020
Xiang 2021b
Tennant 2020
Chan 2019a
Kipping 2012
Goldman 2021a
Chan 2019b
Gold 2021
Groninger 2021
Litwin 2021
Xiang 2021a
Austin 2020
Hua 2015
Nunna 2019
Darnall 2020
Gulsen 2020
Sarig Bahat 2015
Morales Tejera 2020
Nusser 2021
Sarig Bahat 2017
Hwang 2014
Jin 2018
Yilmaz Yelvar 2017
Jin 2016
Mukherjee 2020
Azurdia 2018
0
−1
−2
−3
−4
Treatment effect (standarized mean difference)
Standard of Care / No distraction
Distraction and Standard of Care
Standard Therapy
Type of controlgroup

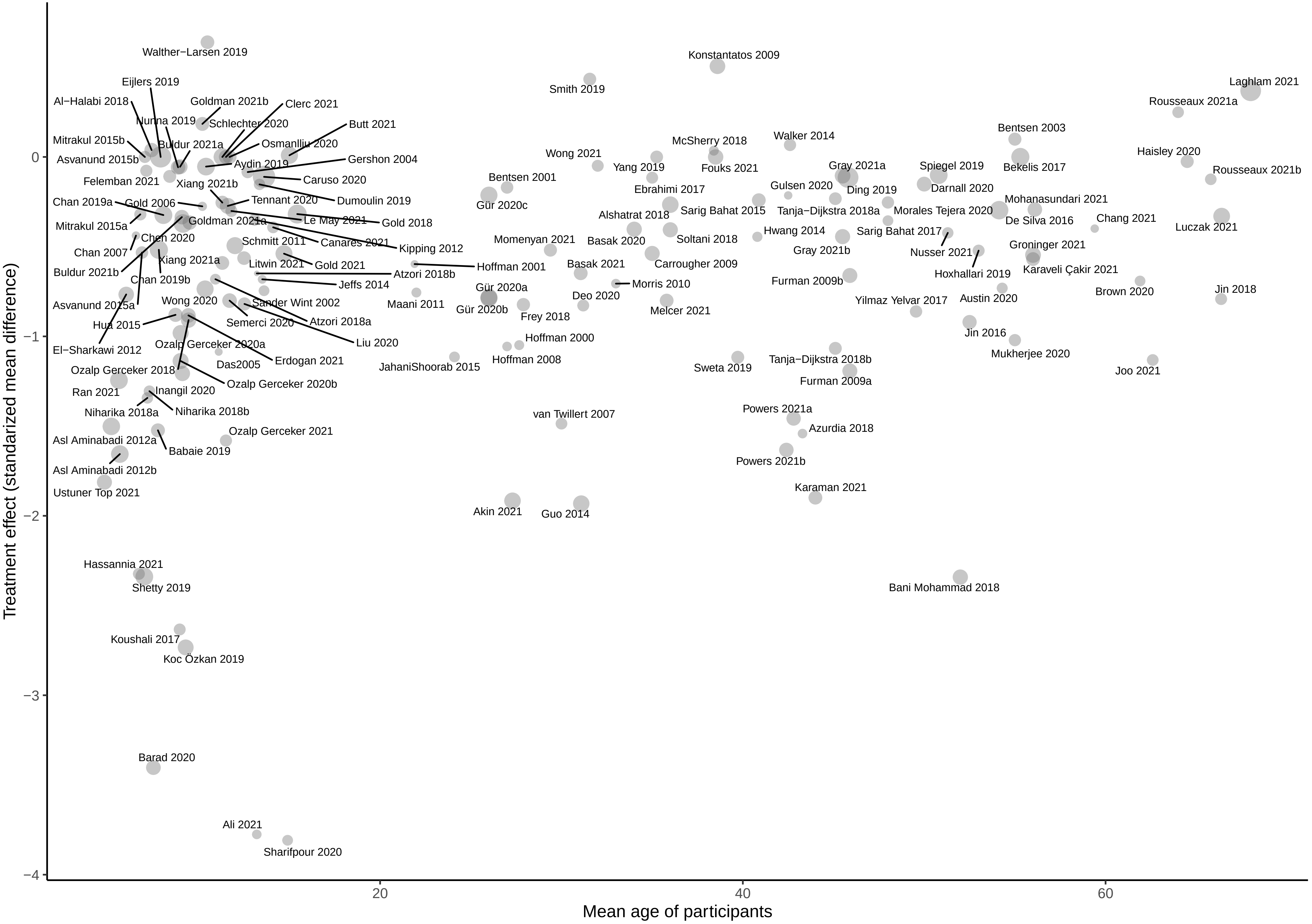

Treatment effect (standarized mean difference)
Mean age of participants
0
−1
−2
−3
−4
20
40
60
Walther−Larsen 2019
Konstantatos 2009
Smith 2019
Laghlam 2021
Rousseaux 2021a
Eijlers 2019
Al−Halabi 2018
Goldman 2021b
Clerc 2021
Nunna 2019
Schlechter 2020
Butt 2021
Mitrakul 2015b
Buldur 2021a
Osmanlliu 2020
Asvanund 2015b
Aydin 2019
Gershon 2004
Felemban 2021
Caruso 2020
Xiang 2021b
Dumoulin 2019
Chan 2019a
Gold 2006
Tennant 2020
Le May 2021
Gold 2018
Mitrakul 2015a
Goldman 2021a
Chen 2020
Schmitt 2011
Canares 2021
Kipping 2012
Chan 2007
Xiang 2021a
Litwin 2021
Gold 2021
Buldur 2021b
Chan 2019b
Atzori 2018b
Jeffs 2014
Asvanund 2015a
Wong 2020
Sander Wint 2002
Semerci 2020
Atzori 2018a
Hua 2015
Liu 2020
El−Sharkawi 2012
Ozalp Gerceker 2020a
Das2005
Erdogan 2021
Ozalp Gerceker 2018
Ozalp Gerceker 2020b
Ran 2021
Inangil 2020
Niharika 2018a
Niharika 2018b
Asl Aminabadi 2012a
Babaie 2019
Ozalp Gerceker 2021
Asl Aminabadi 2012b
Ustuner Top 2021
Hassannia 2021
Shetty 2019
Koushali 2017
Koc Özkan 2019
Barad 2020
Ali 2021
Sharifpour 2020
Wong 2021
Bentsen 2001
Gür 2020c
Momenyan 2021
Hoffman 2001
Maani 2011
Gür 2020a
Gür 2020b
Frey 2018
Hoffman 2000
Hoffman 2008
JahaniShoorab 2015
van Twillert 2007
Akin 2021
Guo 2014
McSherry 2018
Walker 2014
Yang 2019
Fouks 2021
Ebrahimi 2017
Gulsen 2020
Alshatrat 2018
Sarig Bahat 2015
Tanja−Dijkstra 2018a
Basak 2020
Soltani 2018
Hwang 2014
Basak 2021
Carrougher 2009
Morris 2010
Deo 2020
Melcer 2021
Furman 2009b
Sweta 2019
Tanja−Dijkstra 2018b
Furman 2009a
Powers 2021a
Azurdia 2018
Powers 2021b
Karaman 2021
Bani Mohammad 2018
Gray 2021a
Ding 2019
Gray 2021b
Sarig Bahat 2017
Nusser 2021
Morales Tejera 2020
Yilmaz Yelvar 2017
Jin 2016
Bentsen 2003
Spiegel 2019
Darnall 2020
Bekelis 2017
Mohanasundari 2021
De Silva 2016
Groninger 2021
Hoxhallari 2019
Karaveli Çakir 2021
Austin 2020
Mukherjee 2020
Haisley 2020
Rousseaux 2021b
Chang 2021
Luczak 2021
Brown 2020
Jin 2018
Joo 2021

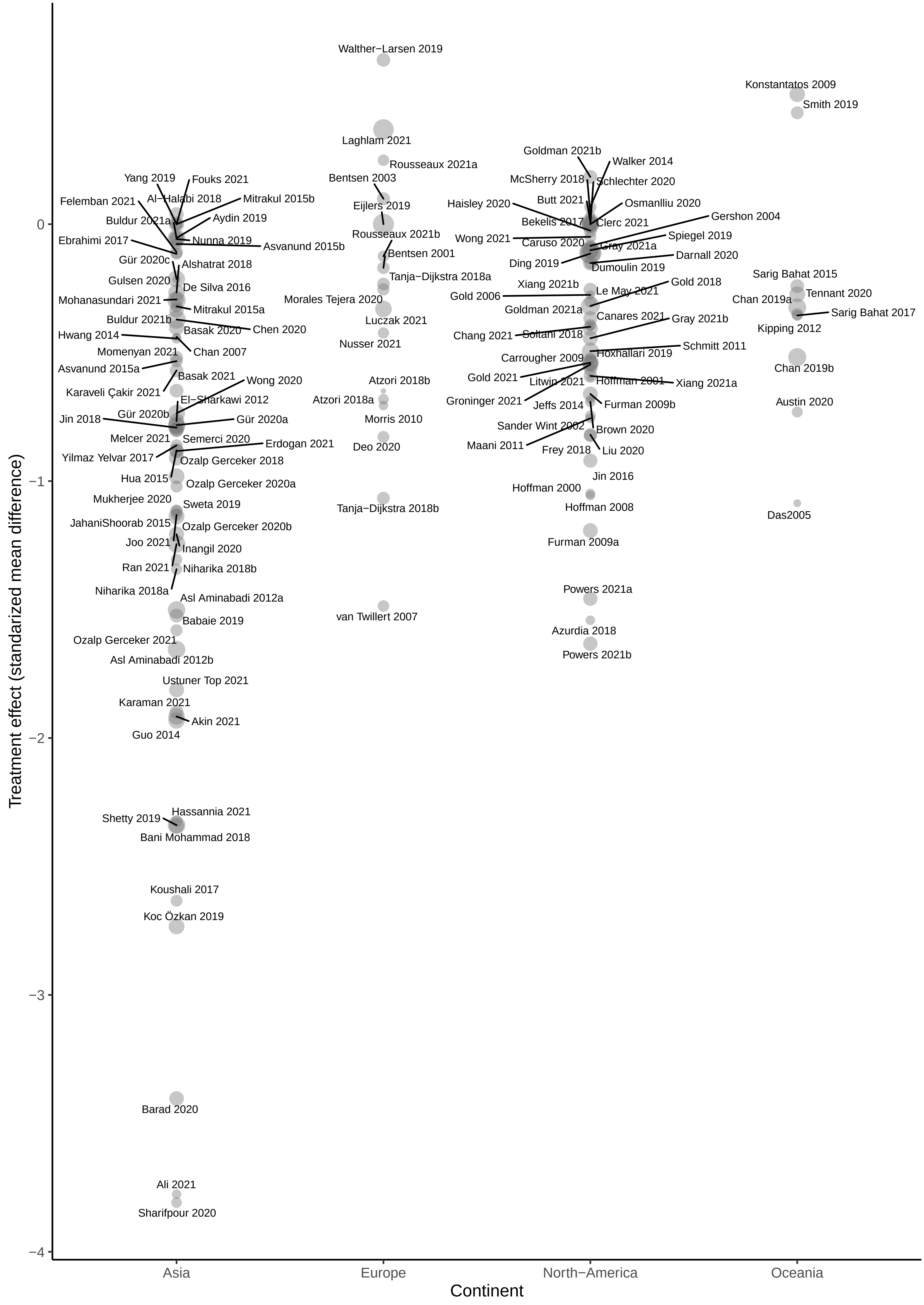
Treatment effect (standarized mean difference)
0
−1
−2
−3
−4
Continent
Asia
Europe
North−America
Oceania
Walther−Larsen 2019
Konstantatos 2009
Smith 2019
Laghlam 2021
Goldman 2021b
Walker 2014
Rousseaux 2021a
McSherry 2018
Schlechter 2020
Yang 2019
Fouks 2021
Bentsen 2003
Butt 2021
Osmanlliu 2020
Felemban 2021
Al−Halabi 2018
Mitrakul 2015b
Haisley 2020
Eijlers 2019
Gershon 2004
Aydin 2019
Bekelis 2017
Clerc 2021
Buldur 2021a
Rousseaux 2021b
Spiegel 2019
Ebrahimi 2017
Nunna 2019
Wong 2021
Caruso 2020
Asvanund 2015b
Gray 2021a
Bentsen 2001
Darnall 2020
Gür 2020c
Alshatrat 2018
Ding 2019
Dumoulin 2019
Tanja−Dijkstra 2018a
Sarig Bahat 2015
Gulsen 2020
Gold 2018
Xiang 2021b
De Silva 2016
Le May 2021
Tennant 2020
Gold 2006
Mohanasundari 2021
Morales Tejera 2020
Chan 2019a
Goldman 2021a
Mitrakul 2015a
Sarig Bahat 2017
Canares 2021
Buldur 2021b
Luczak 2021
Gray 2021b
Basak 2020
Chen 2020
Kipping 2012
Hwang 2014
Chang 2021
Soltani 2018
Nusser 2021
Schmitt 2011
Momenyan 2021
Chan 2007
Hoxhallari 2019
Carrougher 2009
Asvanund 2015a
Chan 2019b
Basak 2021
Gold 2021
Wong 2020
Atzori 2018b
Litwin 2021
Hoffman 2001
Xiang 2021a
Karaveli Çakir 2021
El−Sharkawi 2012
Atzori 2018a
Groninger 2021
Austin 2020
Jeffs 2014
Furman 2009b
Gür 2020b
Jin 2018
Gür 2020a
Morris 2010
Sander Wint 2002
Brown 2020
Melcer 2021
Semerci 2020
Erdogan 2021
Maani 2011
Deo 2020
Frey 2018
Liu 2020
Yilmaz Yelvar 2017
Ozalp Gerceker 2018
Hua 2015
Jin 2016
Ozalp Gerceker 2020a
Hoffman 2000
Mukherjee 2020
Sweta 2019
Tanja−Dijkstra 2018b
Hoffman 2008
Das2005
JahaniShoorab 2015
Ozalp Gerceker 2020b
Joo 2021
Furman 2009a
Inangil 2020
Ran 2021
Niharika 2018b
Niharika 2018a
Powers 2021a
Asl Aminabadi 2012a
van Twillert 2007
Babaie 2019
Azurdia 2018
Ozalp Gerceker 2021
Powers 2021b
Asl Aminabadi 2012b
Ustuner Top 2021
Karaman 2021
Akin 2021
Guo 2014
Hassannia 2021
Shetty 2019
Bani Mohammad 2018
Koushali 2017
Koc Özkan 2019
Barad 2020
Ali 2021
Sharifpour 2020

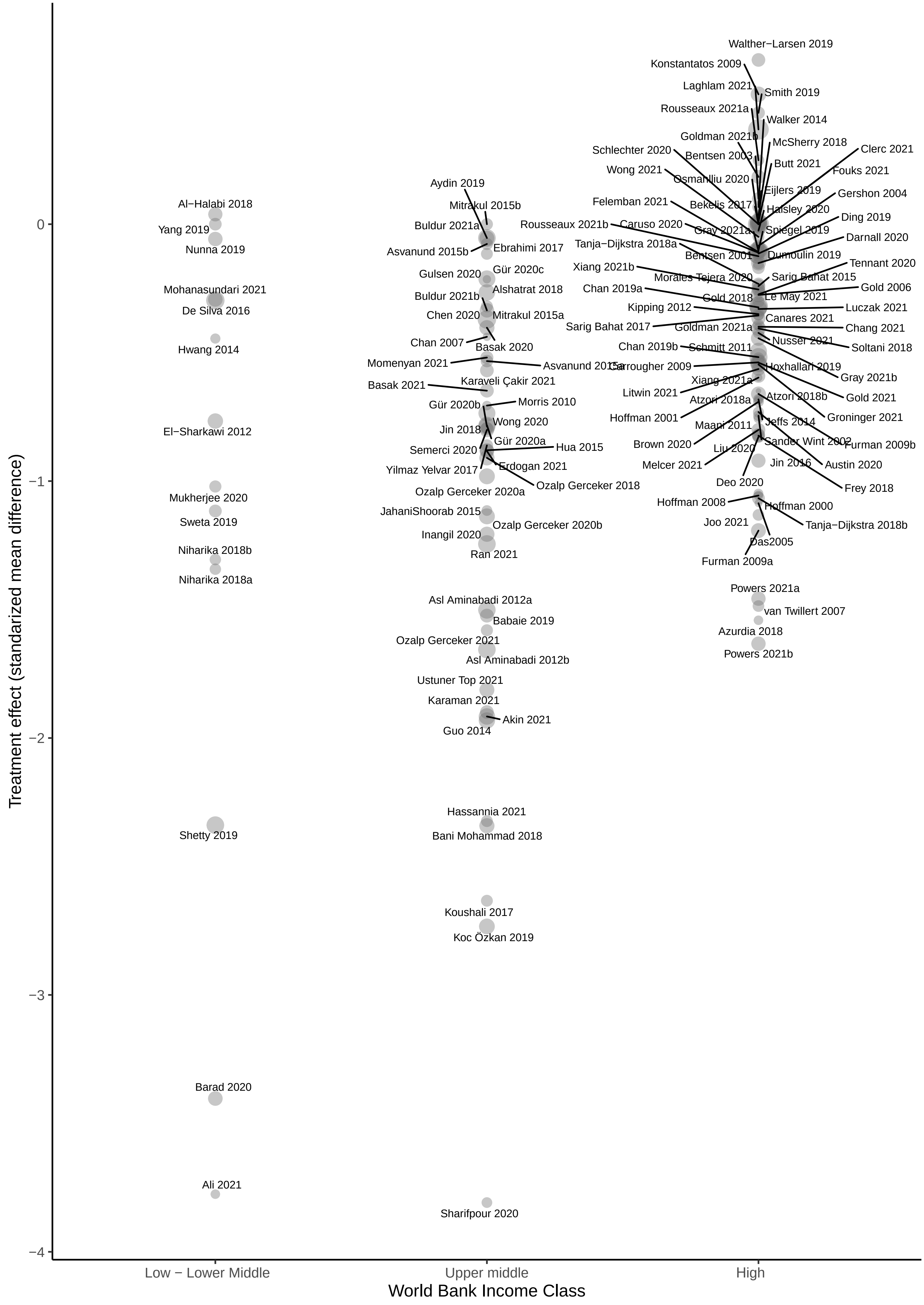
Treatment effect (standarized mean difference)
0
−1
−2
−3
−4
World Bank Income Class
Low − Lower Middle
Upper middle
High
Al−Halabi 2018
Yang 2019
Nunna 2019
Mohanasundari 2021
De Silva 2016
Hwang 2014
El−Sharkawi 2012
Mukherjee 2020
Sweta 2019
Niharika 2018b
Niharika 2018a
Shetty 2019
Barad 2020
Ali 2021
Aydin 2019
Mitrakul 2015b
Buldur 2021a
Asvanund 2015b
Ebrahimi 2017
Gulsen 2020
Gür 2020c
Alshatrat 2018
Buldur 2021b
Chen 2020
Mitrakul 2015a
Chan 2007
Basak 2020
Momenyan 2021
Asvanund 2015a
Karaveli Çakir 2021
Basak 2021
Gür 2020b
Morris 2010
Wong 2020
Jin 2018
Gür 2020a
Hua 2015
Semerci 2020
Erdogan 2021
Yilmaz Yelvar 2017
Ozalp Gerceker 2018
Ozalp Gerceker 2020a
JahaniShoorab 2015
Ozalp Gerceker 2020b
Inangil 2020
Ran 2021
Asl Aminabadi 2012a
Babaie 2019
Ozalp Gerceker 2021
Asl Aminabadi 2012b
Ustuner Top 2021
Karaman 2021
Akin 2021
Guo 2014
Hassannia 2021
Bani Mohammad 2018
Koushali 2017
Koc Özkan 2019
Sharifpour 2020
Walther−Larsen 2019
Konstantatos 2009
Laghlam 2021
Smith 2019
Rousseaux 2021a
Walker 2014
Goldman 2021b
McSherry 2018
Schlechter 2020
Bentsen 2003
Clerc 2021
Butt 2021
Wong 2021
Fouks 2021
Osmanlliu 2020
Eijlers 2019
Gershon 2004
Felemban 2021
Bekelis 2017
Halsley 2020
Ding 2019
Rousseaux 2021b
Caruso 2020
Gray 2021a
Spiegel 2019
Darnall 2020
Tanja−Dijkstra 2018a
Bentsen 2001
Dumoulin 2019
Tennant 2020
Xiang 2021b
Morales Tejera 2020
Sarig Bahat 2015
Chan 2019a
Gold 2006
Gold 2018
Le May 2021
Kipping 2012
Luczak 2021
Canares 2021
Sarig Bahat 2017
Goldman 2021a
Chang 2021
Nusser 2021
Chan 2019b
Schmitt 2011
Soltani 2018
Carrougher 2009
Hoxhallari 2019
Xiang 2021a
Gray 2021b
Litwin 2021
Atzori 2018b
Gold 2021
Atzori 2018a
Hoffman 2001
Jeffs 2014
Groninger 2021
Maani 2011
Sander Wint 2002
Brown 2020
Furman 2009b
Liu 2020
Jin 2016
Austin 2020
Melcer 2021
Deo 2020
Frey 2018
Hoffman 2008
Hoffman 2000
Joo 2021
Tanja−Dijkstra 2018b
Das2005
Furman 2009a
Powers 2021a
van Twillert 2007
Azurdia 2018
Powers 2021b

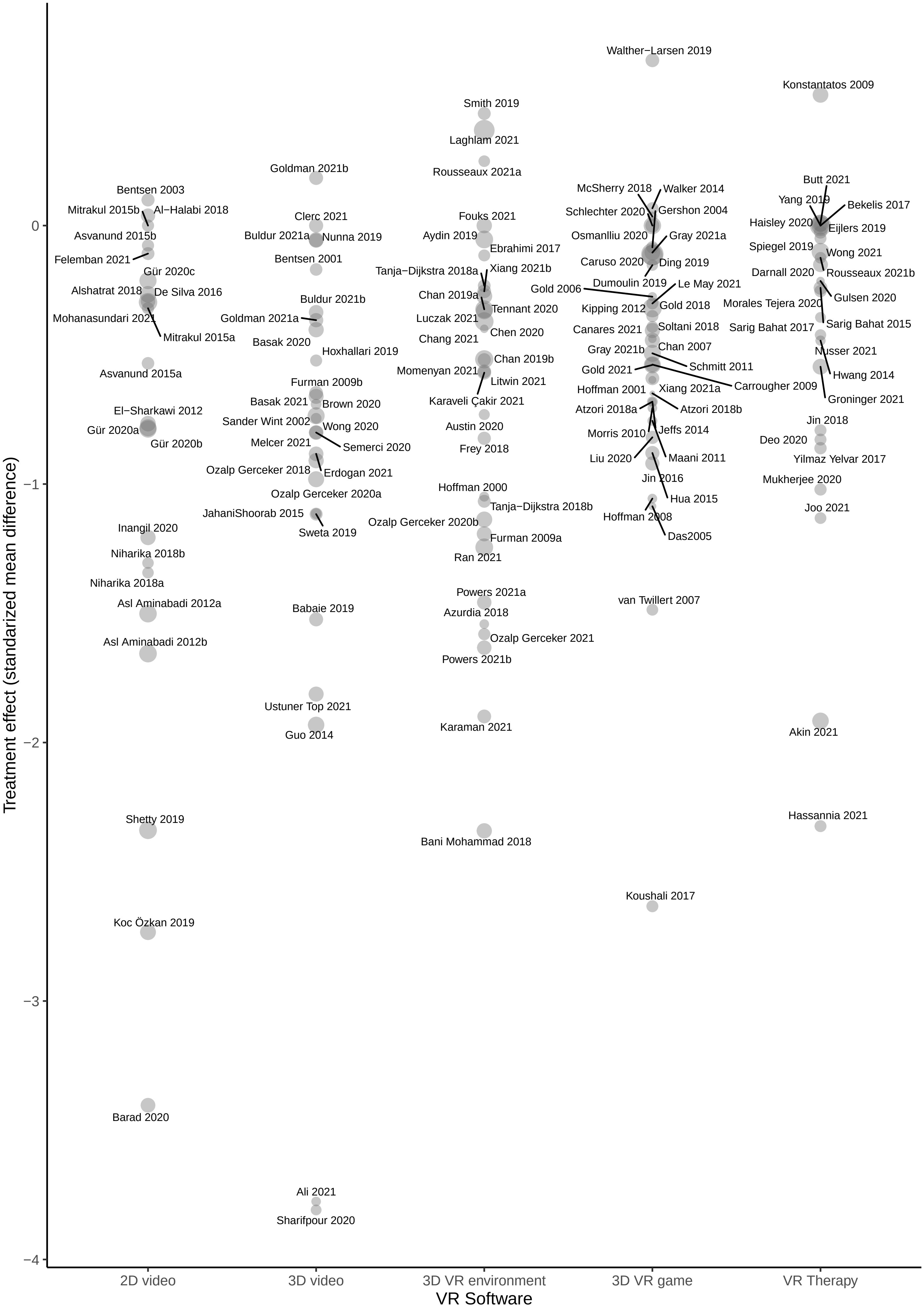
Treatment effect (standarized mean difference)
VR Software
2D video
3D video
3D VR environment
3D VR game
VR Therapy
0
−1
−2
−3
−4
Bentsen 2003
Mitrakul 2015b
Al−Halabi 2018
Asvanund 2015b
Felemban 2021
Gür 2020c
Alshatrat 2018
De Silva 2016
Mohanasundari 2021
Mitrakul 2015a
Asvanund 2015a
El−Sharkawi 2012
Gür 2020a
Gür 2020b
Inangil 2020
Niharika 2018b
Niharika 2018a
Asl Aminabadi 2012a
Asl Aminabadi 2012b
Shetty 2019
Koc Özkan 2019
Barad 2020
Goldman 2021b
Clerc 2021
Buldur 2021a
Nunna 2019
Bentsen 2001
Buldur 2021b
Goldman 2021a
Basak 2020
Hoxhallari 2019
Furman 2009b
Basak 2021
Brown 2020
Sander Wint 2002
Wong 2020
Melcer 2021
Semerci 2020
Ozalp Gerceker 2018
Erdogan 2021
Ozalp Gerceker 2020a
JahaniShoorab 2015
Sweta 2019
Babaie 2019
Ustuner Top 2021
Guo 2014
Ali 2021
Sharifpour 2020
Smith 2019
Laghlam 2021
Rousseaux 2021a
Fouks 2021
Aydin 2019
Ebrahimi 2017
Tanja−Dijkstra 2018a
Xiang 2021b
Chan 2019a
Tennant 2020
Luczak 2021
Chen 2020
Chang 2021
Chan 2019b
Momenyan 2021
Litwin 2021
Karaveli Çakir 2021
Austin 2020
Frey 2018
Hoffman 2000
Tanja−Dijkstra 2018b
Ozalp Gerceker 2020b
Furman 2009a
Ran 2021
Powers 2021a
Azurdia 2018
Ozalp Gerceker 2021
Powers 2021b
Karaman 2021
Bani Mohammad 2018
Walther−Larsen 2019
McSherry 2018
Walker 2014
Schlechter 2020
Gershon 2004
Osmanlliu 2020
Gray 2021a
Caruso 2020
Ding 2019
Dumoulin 2019
Le May 2021
Gold 2006
Kipping 2012
Gold 2018
Canares 2021
Soltani 2018
Gray 2021b
Chan 2007
Schmitt 2011
Gold 2021
Carrougher 2009
Hoffman 2001
Xiang 2021a
Atzori 2018a
Atzori 2018b
Jeffs 2014
Morris 2010
Liu 2020
Maani 2011
Jin 2016
Hua 2015
Hoffman 2008
Das2005
van Twillert 2007
Koushali 2017
Konstantatos 2009
Butt 2021
Yang 2019
Bekelis 2017
Haisley 2020
Eijlers 2019
Spiegel 2019
Wong 2021
Darnall 2020
Rousseaux 2021b
Gulsen 2020
Morales Tejera 2020
Sarig Bahat 2017
Sarig Bahat 2015
Nusser 2021
Hwang 2014
Groninger 2021
Jin 2018
Deo 2020
Yilmaz Yelvar 2017
Mukherjee 2020
Joo 2021
Akin 2021
Hassannia 2021

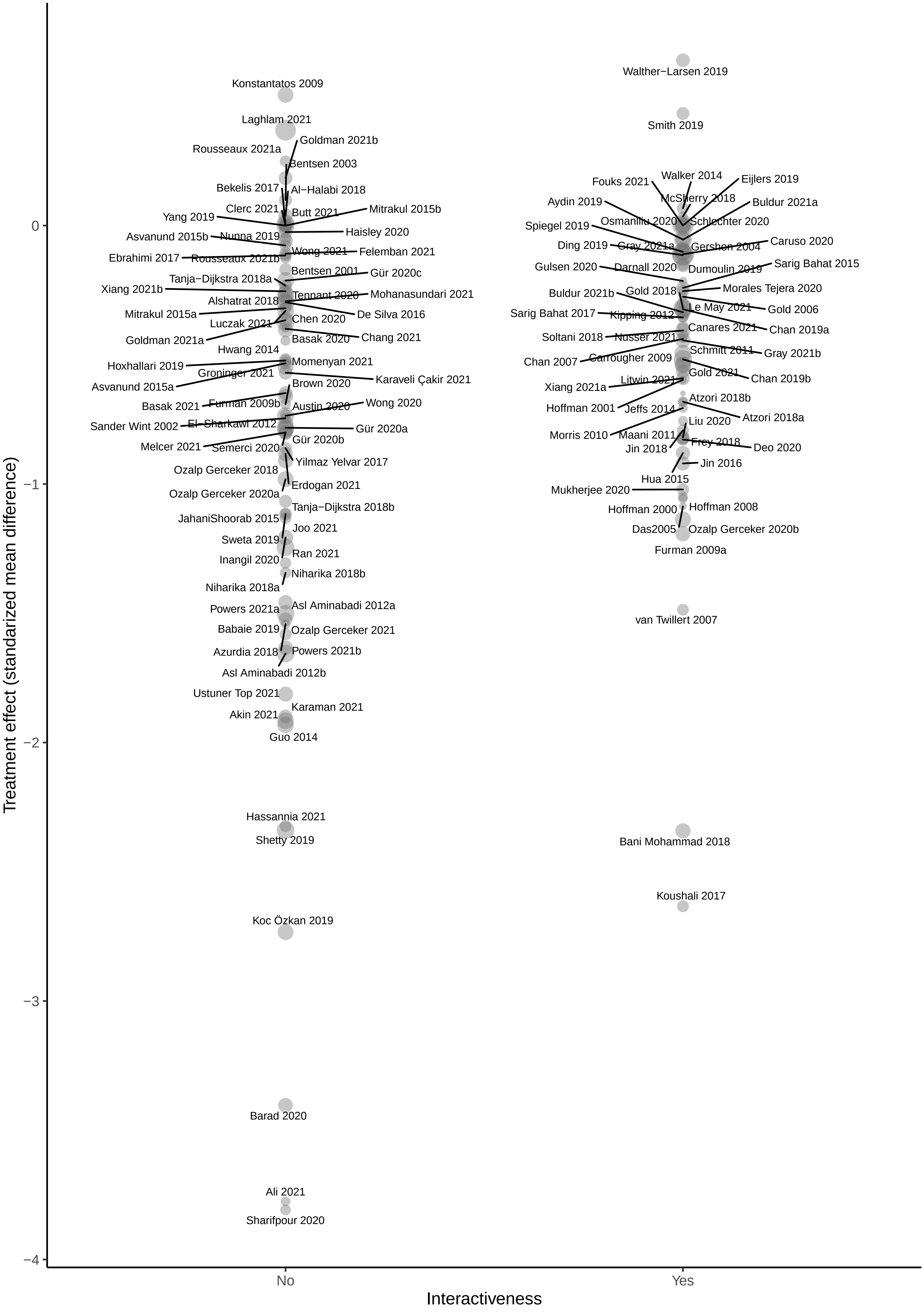

Treatment effect (standarized mean difference)
0
−1
−2
−3
−4
Interactiveness
No
Yes
Konstantatos 2009
Laghlam 2021
Goldman 2021b
Rousseaux 2021a
Bentsen 2003
Bekelis 2017
Al−Halabi 2018
Clerc 2021
Butt 2021
Mitrakul 2015b
Yang 2019
Haisley 2020
Asvanund 2015b
Nunna 2019
Wong 2021
Felemban 2021
Ebrahimi 2017
Rousseaux 2021b
Bentsen 2001
Gür 2020c
Tanja−Dijkstra 2018a
Xiang 2021b
Tennant 2020
Mohanasundari 2021
Alshatrat 2018
Mitrakul 2015a
De Silva 2016
Chen 2020
Luczak 2021
Goldman 2021a
Basak 2020
Chang 2021
Hwang 2014
Hoxhallari 2019
Momenyan 2021
Groninger 2021
Asvanund 2015a
Karaveli Çakir 2021
Brown 2020
Basak 2021
Furman 2009b
Austin 2020
Wong 2020
Sander Wint 2002
El−Sharkawi 2012
Gür 2020a
Melcer 2021
Gür 2020b
Semerci 2020
Yilmaz Yelvar 2017
Ozalp Gerceker 2018
Erdogan 2021
Ozalp Gerceker 2020a
Tanja−Dijkstra 2018b
JahaniShoorab 2015
Joo 2021
Sweta 2019
Ran 2021
Inangil 2020
Niharika 2018b
Niharika 2018a
Powers 2021a
Asl Aminabadi 2012a
Babaie 2019
Ozalp Gerceker 2021
Azurdia 2018
Powers 2021b
Asl Aminabadi 2012b
Ustuner Top 2021
Karaman 2021
Akin 2021
Guo 2014
Hassannia 2021
Shetty 2019
Koc Özkan 2019
Barad 2020
Ali 2021
Sharifpour 2020
Walther−Larsen 2019
Smith 2019
Fouks 2021
Walker 2014
Eijlers 2019
Aydin 2019
McSherry 2018
Buldur 2021a
Osmanlliu 2020
Schlechter 2020
Spiegel 2019
Caruso 2020
Ding 2019
Gray 2021a
Gershon 2004
Gulsen 2020
Darnall 2020
Dumoulin 2019
Sarig Bahat 2015
Morales Tejera 2020
Buldur 2021b
Gold 2018
Le May 2021
Gold 2006
Sarig Bahat 2017
Kipping 2012
Chan 2019a
Canares 2021
Soltani 2018
Nusser 2021
Schmitt 2011
Gray 2021b
Chan 2007
Carrougher 2009
Gold 2021
Chan 2019b
Litwin 2021
Xiang 2021a
Atzori 2018b
Hoffman 2001
Jeffs 2014
Atzori 2018a
Liu 2020
Morris 2010
Maani 2011
Frey 2018
Deo 2020
Jin 2018
Jin 2016
Hua 2015
Mukherjee 2020
Hoffman 2008
Hoffman 2000
Das2005
Ozalp Gerceker 2020b
Furman 2009a
van Twillert 2007
Bani Mohammad 2018
Koushali 2017

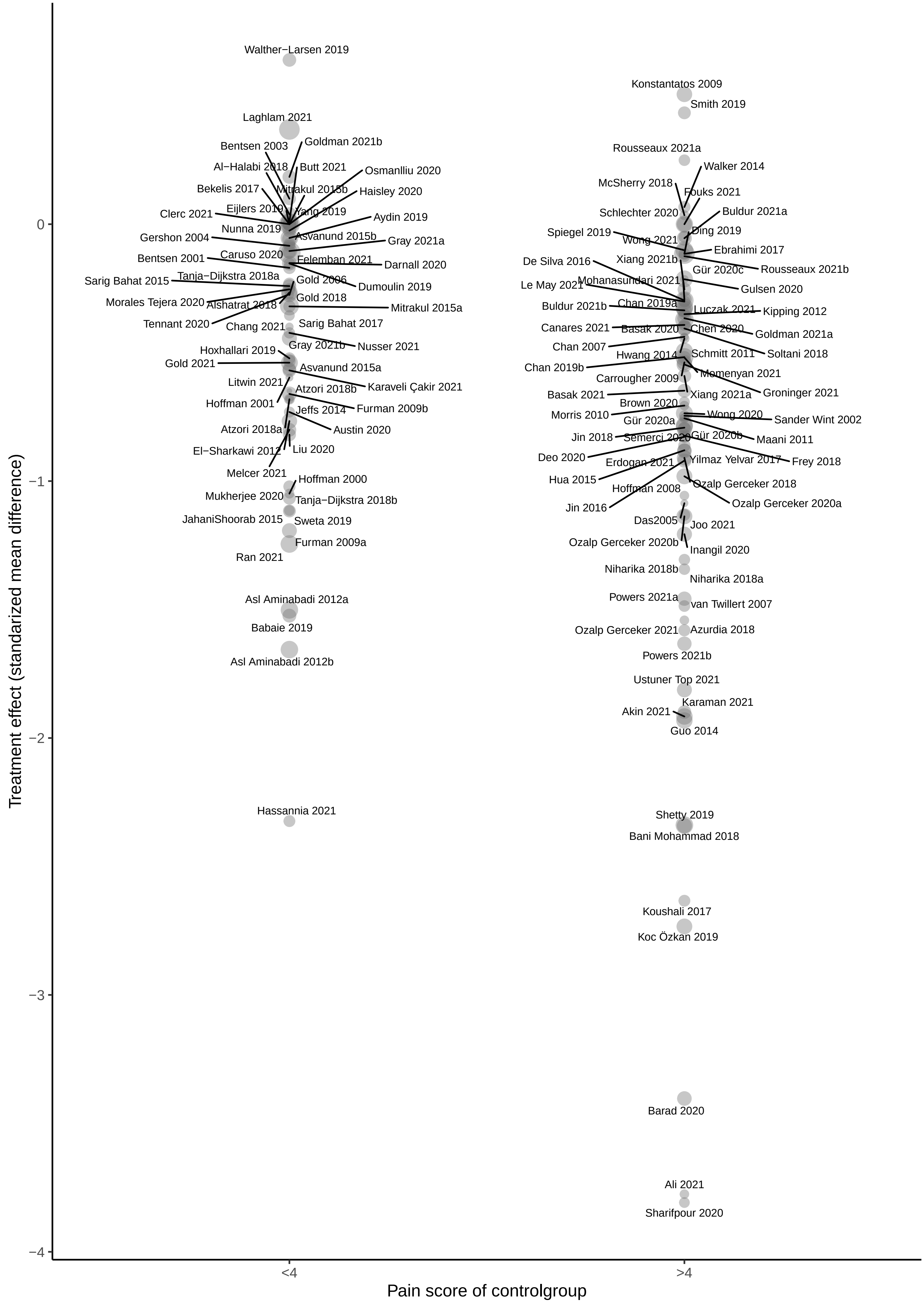

Treatment effect (standarized mean difference)
Pain score of controlgroup
0
−1
−2
−3
−4
<4
>4
Walther−Larsen 2019
Laghlam 2021
Bentsen 2003
Goldman 2021b
Al−Halabi 2018
Butt 2021
Osmanlliu 2020
Bekelis 2017
Mitrakul 2015b
Haisley 2020
Eijlers 2019
Yang 2019
Clerc 2021
Aydin 2019
Nunna 2019
Asvanund 2015b
Gershon 2004
Gray 2021a
Caruso 2020
Bentsen 2001
Felemban 2021
Darnall 2020
Tanja−Dijkstra 2018a
Sarig Bahat 2015
Gold 2006
Dumoulin 2019
Gold 2018
Morales Tejera 2020
Alshatrat 2018
Mitrakul 2015a
Tennant 2020
Chang 2021
Sarig Bahat 2017
Gray 2021b
Nusser 2021
Hoxhallari 2019
Gold 2021
Asvanund 2015a
Litwin 2021
Karaveli Çakir 2021
Atzori 2018b
Hoffman 2001
Furman 2009b
Jeffs 2014
Atzori 2018a
Austin 2020
El−Sharkawi 2012
Liu 2020
Melcer 2021
Hoffman 2000
Mukherjee 2020
Tanja−Dijkstra 2018b
JahaniShoorab 2015
Sweta 2019
Furman 2009a
Ran 2021
Asl Aminabadi 2012a
Babaie 2019
Asl Aminabadi 2012b
Hassannia 2021
Konstantatos 2009
Smith 2019
Rousseaux 2021a
Walker 2014
McSherry 2018
Fouks 2021
Schlechter 2020
Buldur 2021a
Spiegel 2019
Ding 2019
Wong 2021
Ebrahimi 2017
De Silva 2016
Xiang 2021b
Rousseaux 2021b
Gür 2020c
Mohanasundari 2021
Le May 2021
Gulsen 2020
Buldur 2021b
Chan 2019a
Luczak 2021
Kipping 2012
Canares 2021
Basak 2020
Chen 2020
Goldman 2021a
Chan 2007
Schmitt 2011
Soltani 2018
Hwang 2014
Chan 2019b
Momenyan 2021
Carrougher 2009
Groninger 2021
Basak 2021
Xiang 2021a
Brown 2020
Wong 2020
Morris 2010
Sander Wint 2002
Gür 2020a
Jin 2018
Semerci 2020
Gür 2020b
Maani 2011
Deo 2020
Yilmaz Yelvar 2017
Frey 2018
Erdogan 2021
Hua 2015
Ozalp Gercker 2018
Hoffman 2008
Ozalp Gercker 2020a
Jin 2016
Das2005
Joo 2021
Ozalp Gercker 2020b
Inangil 2020
Niharika 2018b
Niharika 2018a
Powers 2021a
van Twillert 2007
Ozalp Gercker 2021
Azurdia 2018
Powers 2021b
Ustuner Top 2021
Karaman 2021
Akin 2021
Guo 2014
Shetty 2019
Bani Mohammad 2018
Koushali 2017
Koc Özkan 2019
Barad 2020
Ali 2021
Sharifpour 2020
